# Macrocyclic Tetramers—Structural Investigation of Peptide-Peptoid Hybrids

**DOI:** 10.3390/molecules26154548

**Published:** 2021-07-28

**Authors:** Claudine Nicole Herlan, Anna Sonnefeld, Thomas Gloge, Julian Brückel, Luisa Chiara Schlee, Claudia Muhle-Goll, Martin Nieger, Stefan Bräse

**Affiliations:** 1 Institute of Organic Chemistry, Karlsruhe Institute of Technology, Fritz-Haber-Weg 6, 76131 Karlsruhe, Germany; claudine.herlan@kit.edu (C.N.H.); julian.brueckel@student.kit.edu (J.B.); luisa.schlee@student.kit.edu (L.C.S.); 2 Institute for Biological Interfaces 4, Karlsruhe Institute of Technology, Hermann-von-Helmholtz-Platz 1, 76344 Eggenstein-Leopoldshafen, Germany; uieuk@student.kit.edu (A.S.); thomas.gloge@kit.edu (T.G.); claudia.muhle-goll@kit.edu (C.M.-G.); 3 Department of Chemistry, University of Helsinki, P.O. Box 55 (A.I. Virtasen aukio 1), FIN-00014 Helsinki, Finland; martin.nieger@helsinki.fi; 4 Institute of Biological and Chemical Systems—Functional Molecular Systems, Karlsruhe Institute of Technology, Hermann-von-Helmholtz-Platz 1, 76344 Eggenstein-Leopoldshafen, Germany

**Keywords:** macrocycles, multiconformational equilibrium, peptidomimetics, peptoids, spatial structure, tetramers

## Abstract

Outstanding affinity and specificity are the main characteristics of peptides, rendering them interesting compounds for basic and medicinal research. However, their biological applicability is limited due to fast proteolytic degradation. The use of mimetic peptoids overcomes this disadvantage, though they lack stereochemical information at the *α*-carbon. Hybrids composed of amino acids and peptoid monomers combine the unique properties of both parent classes. Rigidification of the backbone increases the affinity towards various targets. However, only little is known about the spatial structure of such constrained hybrids. The determination of the three-dimensional structure is a key step for the identification of new targets as well as the rational design of bioactive compounds. Herein, we report the synthesis and the structural elucidation of novel tetrameric macrocycles. Measurements were taken in solid and solution states with the help of X-ray scattering and NMR spectroscopy. The investigations made will help to find diverse applications for this new, promising compound class.

## 1. Introduction

Peptides, a structurally and functionally diverse class of macromolecules, are involved in all parts of life. Their unique properties render them highly promising compounds for biochemical and medicinal research [1,2]. However, peptides come along with some drawbacks limiting their applicability as selective therapeutics: fast proteolytic degradation resulting in low bioavailability and improvable physicochemical properties [3,4].

Cyclization has been shown to increase proteolytic resistance and even the binding affinity and specificity of linear peptides [5,6]. Spatially fixed arrangements of functional moieties arouse outstanding bioactivities, especially in small cyclic peptides [7,8,9,10,11,12]. Nowadays several cyclic tetrapeptides that modify eukaryotic gene expression [13,14,15,16,17,18,19,20], that interact with cellular receptors [11,21,22,23,24,25] or that display antimicrobial activity [12,26,27,28,29,30] are known.

**Figure 1:**
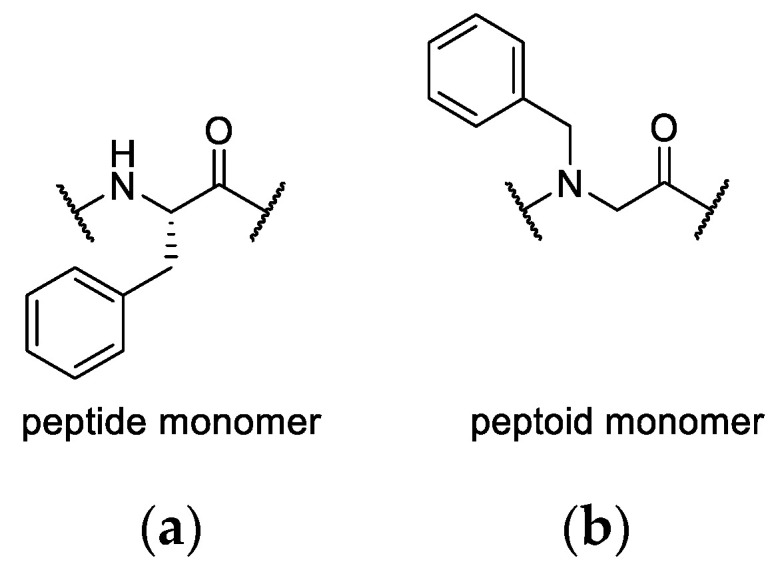
l-Phenylalanine is an example of an amino acid as the monomer of a peptide (**a**) and its respective peptoid monomer (**b**).

Another approach to improve the bioavailability of linear peptides while maintaining their unique characteristics has been modifying the individual building blocks [31,32]. The formal shift of the side chain from the *α*-carbon to the nitrogen atom results in peptoids, which mimic the structure of their parent compounds but lack pivotal motifs affecting the spatial arrangement (Figure 1) [33,34,35,36].

*N*-alkylation in peptoids prevents the formation of backbone hydrogen bonds which are crucial for stabilizing secondary structures in related peptides. The absence of the hydrogen bond donor results in enhanced conformational flexibility that comes along with increased *cis*/*trans*-amide isomerism [37,38,39]. Peptoids of different sizes have been cyclized to constrain their conformational flexibility [40,41,42,43,44,45,46,47]. Structural studies of these macrocycles revealed defined geometries entailing distinct *cis*-*trans* sequences depending on the size and the type of side chains [37,38,40]. 

Besides peptoid macrocycles, several studies on cyclic, *N*-methylated peptides have been reported [48,49,50,51,52,53]. However, little is known about the spatial structure of macrocycles that are built up of natural amino acids and peptoid monomers. These hybrid structures combine the unique selectivity and affinity of peptides with the outstanding metabolic stability of peptoids. To date, only a few representatives of this compound class, which holds great promise for future biochemical and medical research, are known [54,55,56,57,58,59]. Understanding the spatial structure of the peptide-peptoid hybrids allows for the search for potential targets and enables rational drug design. 

Herein, we report the synthesis and structural elucidation of tetramers with different ratios of amino acids to peptoid monomers. Macrocycles made up of four monomers were built by head-to-tail cyclization to constrain their conformational flexibility. Crystallographic data and NMR studies were used to determine the three-dimensional (3D) structures of the resulting peptide-peptoid hybrids in solid and solution states. This structural investigation can be a stepping stone for further research on this promising compound class.

## 2. Results and Discussion

**Figure 2:**
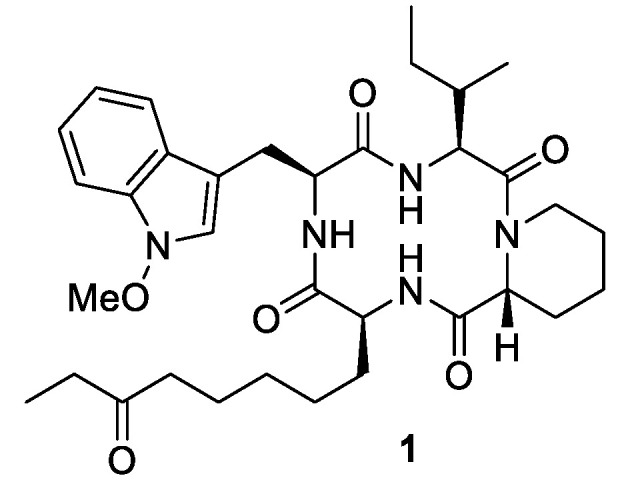
The cyclic tetrapeptide apicidin (**1**).

Initially, we aimed to synthesize a congener library of the cyclic tetrapeptide apicidin (**1**, Figure 2) [60]. The natural fungal metabolite is known for its ability to inhibit histone deacetylases (HDAC) and thus to modify the gene expression in eukaryotic cells [15,61,62,63]. 

Our aim was the design of apicidin derivatives capable of the inhibition of the Wnt/*β*-catenin signaling pathway. Thus, our structures lacked the characteristic l-2-amino-8-oxodecanoic acid (Aoda), which is critical for the HDAC activity of apicidin (**1**) [15]. While analyzing the novel structures, we have observed that replacing individual amino acids with peptoid monomers has an interesting influence on the spatial structure of the macrocycles. Herein, we report our findings based on selecting apicidin congeners with different peptide to peptoid ratios.

Although various chemically and structurally diverse side chains were incorporated into the library of apicidin derivatives, all congeners had an aromatic amino acid and the cyclic, *N*-alkylated amino acid proline in common. Proline was chosen due to its similarity to the building block of the lead structure apicidin (1), namely pipecolic acid, and its decreased energy barrier for *cis*-*trans*-isomerism [64,65,66].

**Scheme 1:**
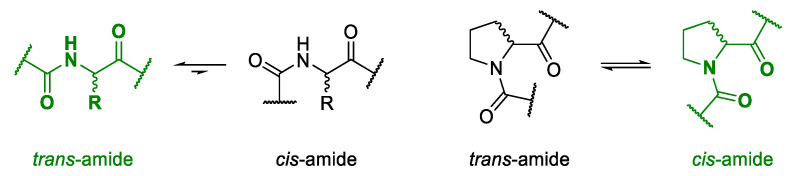
The *cis-trans*-isomerism of peptide bonds.

Peptide bonds are constrained in their free rotatability due to their strong π-character. The energy distribution favors two distinct dihedral angles representing *cis*- and *trans*-amide bonds (Scheme 1) [67,68].

Due to the steric hindrance of their side chains, most amino acids form *trans*-conformations with high energy barriers for *cis*-*trans*-isomerism [67,68]. The unusual structure of proline results in an equimolar distribution of both the *cis-* and the *trans*-conformation when incorporated into a polypeptide [64,65,66]. In nature, isomers of proline are known as loop inducers due to *cis*-bond formation [69,70]. As it is assumed that backbone *cis*-conformations can facilitate the ring closure of tense cyclic tetramers [8,71,72], proline was the building block of choice for the design of different macrocycles.

### 2.1. Synthesis of Macrocyclic Tetramers 

**Scheme 2:**
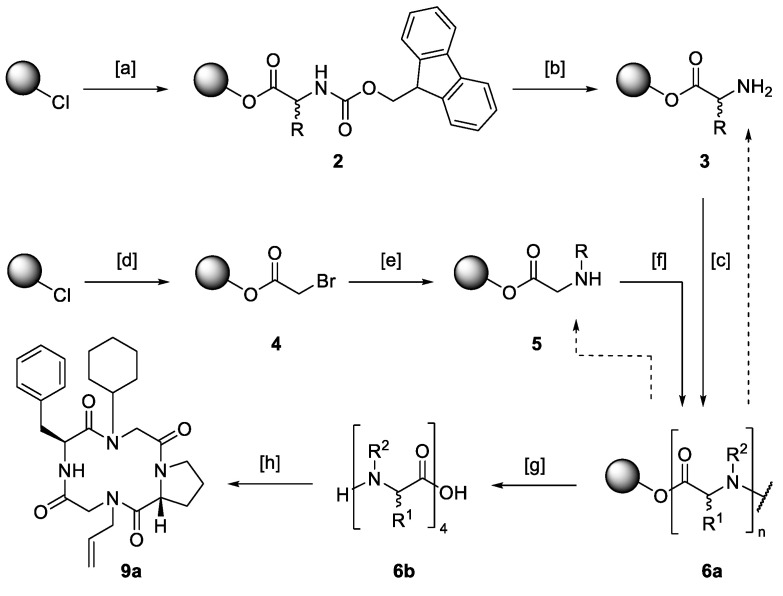
Synthetic protocol of cyclic tetramers. [a]: Fmoc-protected amino acid, *N*,*N*′-diisopropylethylamine (DIPEA), *N*-methyl-2-pyrrolidone (NMP), 21 °C, 16 h; [b]: piperidine, dimethylformamide (DMF), 21 °C, 3 × 5 min; [c]: Fmoc-protected amino acid, *N*,*N*′-diisopropylcarbodiimide (DIC), hydroxybenzotriazole, NMP, 21 °C, 4 h; [d]: bromoacetic acid, DIPEA, DMF, 21 °C, 1 h; [e]: amine, DMF, 21 °C, 1–16 h; [f]: 1. bromoacetic acid, DIC, DMF, 21 °C, 30 min; 2. amine, DMF, 21 °C, 1–16 h; [g]: hexafluoroisopropanol, methylene chloride, 21 °C, 16 h; [h]: [dimethylamino(triazolo[4,5-*b*]pyridin-3-yloxy)methylidene]-di-methylazanium hexafluorophosphate (HATU), DIPEA, DMF, 21 °C, 30 h.

Hybrid structures consisting of amino acids and peptoid monomers were built upon solid support. The synthetic protocol involved the well-known solid-phase peptide synthesis described by Merrifield [73] as well as the submonomer method for the assembly of peptoids published by Zuckermann [33] (Scheme 2). 

The attachment of the *C*-terminal amino acid (→ **2**) or bromoacetic acid as the first submonomer of a peptoid building block (→ **4**) to a 2-chlorotrityl chloride polystyrene resin was performed under basic conditions. In the case of amino acids, the Fmoc-protection group was cleaved using a mixture of 20% piperidine in DMF, resulting in the free primary amine **3**. To build up peptoids, bromoacetic acid was substituted by any desired amine (→ **5**). Depending on the sequence, free amines were either coupled to an amino acid or bromoacetic acid. Diisopropylcarbodiimide was used as a coupling agent in both cases. To avoid racemization, hydroxybenzotriazole was added for the attachment of amino acids.

Acetylation and substitution and amino acid coupling and deprotection were carried out until the desired linear precursor **6** was constructed. Cleavage was performed under mildly acidic conditions releasing a linear tetramer capable of a head-to-tail cyclization. The ring closure was carried out following a protocol by Aldrich [74] with the help of the potent coupling reagent [dimethylamino(triazolo[4,5-*b*]pyridin-3-yloxy)methylidene]-di-methylazanium hexafluoro-phosphate (HATU). This iminium salt is known for its potency in energetically unfavorable couplings, cyclizing constrained tetrapeptides [8,28,75]. To avoid favored side reactions like cyclodimerizations [72,76,77], a 5.00 mM solution of the respective linear precursor was added dropwise to a 2.40 mm solution of HATU.

Reactive moieties of side chains were masked with protecting groups. Deprotection was performed immediately after the cyclization step. After ten or eleven reaction steps, respectively, the synthetic protocol yielded cyclic tetramers, which required only a single purification step at the end of the reaction sequence. Purification was carried out via preparative reversed-phase high performance liquid chromatography (HPLC), and product formation was confirmed via matrix assisted laser desorption ionization-time of flight (MALDI-TOF) mass spectrometry.

In an initial library, several macrocyclic tetrapeptides of general structure **7** were synthesized. To match the model structure apicidin (**1**), proline was incorporated in its d-configuration. The remaining amino acids were applied in their l-configuration. To avoid diketopiperazine formation [78,79], d-proline was incorporated as the third building block during the modular solid-phase synthesis. Approaches with proline as *N*-terminal building block yielded low amounts of the desired macrocycles (data not shown). It was assumed that the low nucleophilicity of the secondary amine prevented cyclization.

For this reason, the sequence of the linear precursors was changed in such a way that a primary amine was in the *N*-terminal position (R^1^). Cyclization of these precursors then led to moderate yields of the corresponding macrocycles. Nine derivatives with structural similarity were selected to represent the library of macrocyclic tetramers (Table 1).

The *N*-terminus of the linear precursors (position R^1^) consisted of branched aliphatic or aromatic amino acids. In position R^2^, different alkyl side chains were incorporated. The *C*-terminus (position R^3^) was built by either l-phenylalanine or l-tryptophan. Ring closure was carried out by amidation of the *N*-terminal amine (R^1^) with the carboxyl function of the *C*-terminal, aromatic amino acid (R^3^). The use of different building blocks did not influence the overall yield of the reaction. Even additional deprotection steps (compounds **7b** and **7g**) had no clear effect on the yields of the macrocyclic tetrapeptides. On average, the cyclic tetramers were isolated in 42% ± 13 overall yield.

In a second library, individual amino acids were replaced by a peptoid monomer. Peptoids are peptidomimetics that promise high metabolic stability and outstanding biological activity [33,34,35]. Compared to peptides, the side chain is formally shifted from the *α*-carbon to the backbone nitrogen atom. This comes with high conformational flexibility as the amide nitrogen loses its capability to serve as a hydrogen bond donor. Moreover, the modification of the amide nitrogen lowers the energy barrier of *cis/trans* isomerization [36,37,80,81]. However, a beneficial effect of this enhanced flexibility on the cyclization reaction was not observed. The nine macrocyclic hybrids representing a library composed of tetramers with three amino acids and one peptoid monomer were isolated in 31% ± 14 overall yields (Table 2).

To resemble the model structure apicidin (**1**), the nine macrocycles **8a–i** have an aromatic amino acid at the *C*-terminal end (R^5^) and an adjacent linear alkyl side chain in common (R^3^ or R^4^). The peptoid monomer was inserted at the *C*- or *N*-terminal position of d-proline (R^1^ or R^3^). In the latter case, cyclization was performed on the secondary amine of a peptoid building block, causing a lower yield on average (19% ± 8, **8a–c**). Incorporating a peptoid monomer in the middle of the sequence resulted in overall yields similar to those obtained for cyclic tetrapeptides (36% ± 13, **8d–i**).

Further peptoid building blocks were incorporated into the macrocycles to enhance structural diversity, resulting in the general structure **9**. Table 3 shows a selection of nine structurally similar apicidin congeners with both aromatic and aliphatic side chains (Table 3).

The macrocyclic hybrids **9a–i** are composed of two peptoid monomers (R^1^ and R^2^) and two amino acids (d-proline and R^3^) located in alternating order on opposite sides of the backbone ring system. Aromatic and linear, and cyclic aliphatic peptoid monomers built the *N*-terminus of the linear precursors (R^1^). The individual building blocks did not influence the overall yields, similar to the yields obtained for hybrids 8a–c that were also cyclized on a secondary amine (24% ± 11).

### 2.2. Multiconformational Equilibrium Detected by NMR

Often multiple signal sets are detected in the nuclear magnetic resonance (NMR) spectra of macrocycles depending on the dielectric properties of the solvent [11,82,83,84]. This could be due to different conformers present or conformational equilibrium [38,85]. Influencing factors are i.e., side chains, solvent effects, and temperature [71,72]. For the model structure apicidin (**1**), as an example, it is known that multiple conformations stem from *cis-trans* isomerism of the pipecolic acid building block [83,86].

HPLC purification of the compounds resulted in sharp peaks indicating that one predominant isomer was synthesized [87,88,89]. However, NMR-spectra of the cyclic tetramers corroborated the formation of several conformers for almost every macrocycle (supplemental, Table S6). This was most prominent for cyclic tetrapeptides **7a–i**, which tended to assemble in multiconformational equilibria due to multiple degrees of freedom.

The same applied to the macrocycles with one peptoid monomer. Macrocycles **8a–i** revealed multiple signal sets in solution, indicating different conformers’ formation (supplemental, Table S6). Nuclear Overhauser and exchange spectroscopy (NOESY) spectra of macrocycle **8f**, for example, led to the identification of five separate conformers which interconverted on the NMR timescale. Surprisingly, the complexity of the spectra of the hybrids **8a–d** was significantly reduced compared to spectra of structures **8e–i**. The peptoid monomer was inserted at the *N*-terminus in the former ones, resulting in one dominant structure next to another isomer in approximately 5:1. Therefore, incorporating a peptoid building block in this position could stabilize distinct isomers, decisive for biological applications.

**Figure 3:**
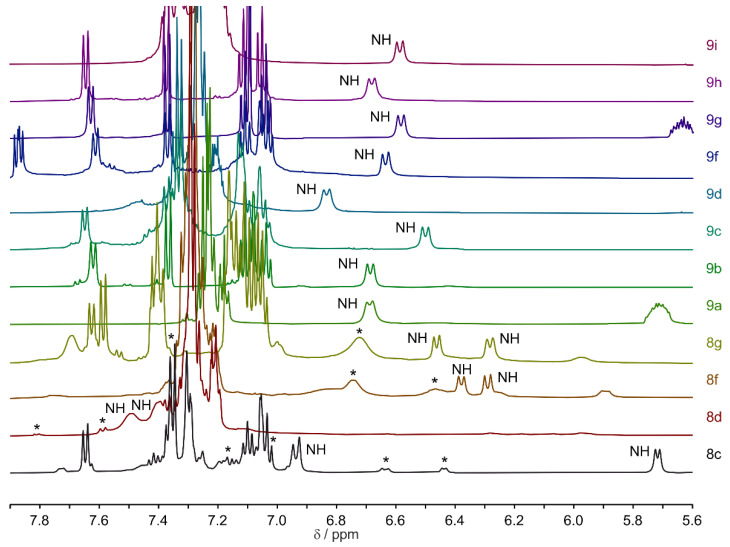
Excerpts from ^1^H NMR spectra of selected macrocycles from series **8** and **9**. An asterisk (*) indicates the NH signals belonging to a signal set of lower intensity. The spectra were normalized to have similar NH intensity.

For macrocycles **9a–i**, one dominant signal set was mostly observed (supplemental, Table S6). To illustrate this, Figure 3 displays the NH regions of selected macrocycles from series **8** and **9,** which were soluble in pure acetonitrile. In the NH region of series **9** macrocycles, only one peptide bond amide signal is visible. For macrocycles **8c**, **8d**, **8f**, and **8g**, one main signal set was accompanied by a second or third signal set of lesser intensity. Macrocycles from series **7** are not shown here, as the molecules were primarily soluble in dimethyl sulfoxide (DMSO) (see supplemental, Table S6).

### 2.3. Spatial Structure in Solid-State

**Figure 4:**
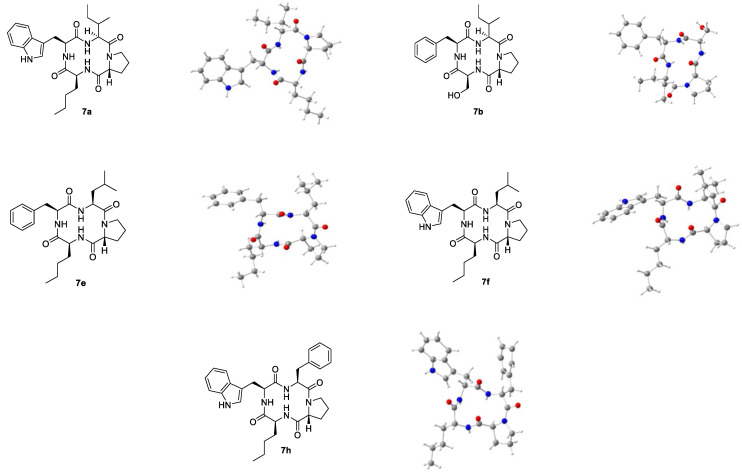
Molecular structure of the cyclic tetrapeptides **7a** and **7b** and one of the independent crystallographic molecules of macrocycles **7e**, **7f** and **7h**.

X-ray diffraction is a highly reliable method to determine the spatial structure of molecules in the solid-state [90,91]. Crystallization of the macrocyclic hybrids was attempted via evaporation of acetonitrile, isopropanol, and methanol. Most macrocycles aggregated into amorphous powders during this process, some became viscous oils, and others produced polycrystalline needle-shaped structures. However, some single crystals were obtained from multiple attempts for each of the five similar cyclic tetrapeptides **7a**, **7b**, **7e**, **7f** and **7h** (Figure 4).

Macrocycle **7b** represents the only tetrapeptide with a polar building block crystallized in sufficient quality for X-ray diffraction. In contrast to the other structures, **7b** is equipped with l-serine instead of the alkyl side chain l-norleucine. Thus, the macrocycle **7b** is the only member of the apicidin tetrapeptide library without l-norleucine that crystallized upon vapor diffusion.

For both tetrapeptides **7a** and **7b** containing an *N*-terminal l-isoleucyl residue, the structure of one isomer was determined via X-ray diffraction. As for the model structure apicidin (**1**) [84], at least three independent structures (I, II, III etc.) each were obtained for macrocycles **7e**, **7f** and **7h**. Their dihedral angles differ slightly from each other but show the same *cis-trans* arrangement (Table 4).

To elucidate the backbone conformation, dihedral angles of the individual macrocycles were measured. The dihedral angle ω describes the torsion angle of the axis between the *α*- and the amide carbon atom of one amino acid and the axis between the amide nitrogen and the *α*-carbon atom of the following building block. Due to the partial double-bond character of the peptide bond, this angle is forced into two distinct values: ω = 0° or ω = ±180°. Sterical hindrance can lead to a deviation of the dihedral angles from their ideal values, but an angle close to ω = 0° indicates a *cis*-conformation while ω = ±180° indicates a *trans*-peptide bond [92].

All conformers of the five macrocycles **7a**, **7b**, **7e**, **7f,** and **7h** showed a *cis*-conformation between the nitrogen atom of their respective d-prolyl residue and the amide carbon of the adjacent amino acid (ω_C_). The *trans-trans-cis-trans* sequence of the backbone has also been reported for the model structure apicidin (**1**) [83] and similar cyclotetrapeptides [93]. The largest deviations from the ideal dihedral angle were measured between the nitrogen atoms of the large aromatic side chains l-phenylalanine or l-tryptophan and the amide carbon of the following building blocks (ω_A_).

The measurements of the configurations of the *α*-carbon atoms showed the expected stereochemistry: the *α*-carbon of every d-proline building block was (*R*)-, the ones of the remaining amino acids were (*S*)-configurated. Furthermore, the macrocycles resembled each other in the location of their side chains: while the aliphatic ring of d-proline pointed above the ring level, the remaining side chains were located below.

Crystallization preparations of hybrids containing one peptoid monomer provided single crystals of two compounds: **8e** and **8f** (Table 5). Both revealed strong structural similarities to the cyclic tetrapeptides **7a–i**. Moreover, the macrocycles **8e** and **8f** are equivalent to each other in large parts of their structure but differ in their peptoid-based alkyl side chain length.

The dihedral angles of the peptide-peptoid hybrids resemble the ones measured for cyclic tetrapeptides. Again, a *cis*-conformation was measured between the nitrogen atom of d-proline and the amide carbon of the following building block. As for tetrapeptides of general structure **7**, three residues were located on the same side of the ring plane while the alkyl ring of d-proline pointed towards the opposite direction.

We could not successfully crystallize any cyclic hybrid of compounds with two peptoid units (series **9**). Thus, we decided to use NMR data for the structure elucidation of the exemplarily chosen macrocycle **9a**.

### 2.4. Spatial Structure in Solution State

Structural information on **9a** was obtained by recording NOESY spectra. Internuclear distances were calculated from NOE cross-peak intensities (see supplemental, Table S2 for details). Using the internuclear distance data from NOESY spectra and dihedral angle information from *J*-coupling constants, a 3D model for **9a** was constructed with the molecular modeling software Avogadro [94]. This model was further structurally optimized utilizing a discrete Fourier transform (DFT) approach using the quantum chemical calculation software Turbomole [95].

However, nuclear Overhauser effect (NOE) data of small molecules is often not sufficient to unambiguously select for one conformation, especially in structural backbone dynamics. Additional structural information regarding **9a** was obtained by extraction of one- and two-bond residual dipolar couplings (RDCs) in a uniaxially stretched polyethylene glycol (PEG) gel [96]. This was achieved by recording clean in-phase heteronuclear single quantum coherence (CLIP-HSQC) [97] and P.E.HSQC [98] spectra of the molecule in an isotropic environment and under anisotropic conditions in a uniaxially stretched polyethylene glycol (PEG) gel [96]. The RDCs were then used to validate the NOE-derived structure. To assess whether the RDCs agree with the constructed model, they were analyzed using single value decomposition (SVD) in the MSpin-RDC software [99]. An SVD omitting the RDC data of the more mobile sidechains yielded acceptable results. The back-calculated and experimental RDCs were in good agreement, with 7 out of 8 RDCs fulfilled within the experimental error (supplemental, Table S3). A full back-calculation including sidechain RDCs can be found in the supporting information (supplemental, Table S3). Although the deviation between experimental and back-calculated values was higher in this case, all RDC values were reasonably well reproduced. The constructed model is therefore largely in agreement with the experimental NOE, *J*-coupling, and RDC data and can be seen to represent the dominant solution state structure of **9a**.

The 3D model of **9a** indicated interesting structural differences compared to the macrocycles with three or four amino acids (Table 6).

The model of **9a** displays an alternating *cis*-*trans*-configuration of the cyclic backbone and an overall oblong ring shape. Thereby, the torsion angles ω_A_ and ω_C_ indicate a *trans*-peptide bond between the amino functions of both amino acids and the carbonyl moieties of the subsequent peptoid monomers. In contrast to previous structures, no *cis*-bond was measured between the nitrogen atom of proline and the amide carbon of the following building block (ω_C_). Instead, two *cis*-bonds were detected between the nitrogen atoms of the peptoid monomers and the subsequent carbonyl carbon atoms (ω_B_ and ω_D_). Likewise, the dihedral angle ω_A_ next to the sterically demanding side chains of l-phenylalanine was no longer distorted from ideal values (ω_A_ = 179.3°). Previous studies on small cyclic peptoids have shown that the *cis*-*trans*-*cis*-*trans* arrangement represents the lowest energy conformation and forms during the crystallization process of different cyclic tetrapeptoids [100,101,102]. Our data indicate that this characteristic backbone arrangement is also favored in cyclic hybrids of general structure **9**. Thus, with an increase in the peptide-peptoid ratio, the backbone configuration of apicidin derivatives can be easily modified.

Besides backbone configuration, the side chains of **9a** differed from previous derivatives: the side chains were located alternately above and below the ring plane. This characteristic orientation is also known for pure peptoid macrocycles of different ring sizes [41,100,101] and various *N*-alkylated tetrapeptides [103,104,105,106,107,108].

Our data indicate that the increase of the peptoid-to-peptide ratio leads to significant structural changes of the entire macrocycle, which must be considered when developing potential inhibitors of the Wnt/*β*-catenin pathway.

## 3. Materials and Methods

Solvents and reagents were purchased from commercial sources (ABCR, Alfa Aesar, Carbolution Chemicals, Chempur, Fisher Chemical, Merck Millipore^®^, Carl Roth and VWR™) and used without further purification. Detailed information on the synthesis of peptoids, reagents, methods and instruments is given in the supplemental information. 

**General procedure for the synthesis of cyclic peptoids:** In a fritted syringe, a 2-chlorotrityl-chloride resin (125 mg, 200 µmol, 1.60 mmol/mg loading density, 100–200 mesh, 1.00 equiv.) was swollen in methylene chloride (DCM) for at least 30 min at 21 °C. After filtration, either a freshly prepared solution of bromoacetic acid (8.00 equiv.) and *N,N*′-diisopropylethylamine (DIPEA, 8.00 equiv.) in *N*,*N*′-dimethyl-formamide (DMF) or a Fmoc-protected amino acid (4.00 equiv.) and DIPEA (4.00 equiv.) in *N*-methylpyrrolidone (NMP) was added and shaken for 1 h or rather 16 h at 21 °C. The resin was extensively washed with DMF, methanol, and DCM. In the former case, a solution of the corresponding amine (8.00 equiv.) in DMF was added to the resin and shaken for 1 h at 21 °C. In the latter case, a solution of 20% piperidine in DMF was repeatedly added. Following extensive washing, either a solution of bromoacetic acid (8.00 equiv.) and *N*,*N*′-diisopropyl-carbodiimide (DIC, 8.00 equiv.) in DMF or a Fmoc-protected amino acid (4.00 equiv.), 1-hydroxybenzotriazole (HOBt, 4.00 equiv.) and DIC (4.00 equiv.) in NMP were added and shaken for 30 min or 4 h at 21 °C. Substitution or rather Fmoc-deprotection and acetylation or rather amino acid coupling were alternated repeatedly until the desired tetramer was built. For cleavage, a solution of 33% hexafluoroisopropanol in DCM was added, and the mixture was shaken overnight. The solvent was removed under an air stream.

For the cyclization, a solution of the respective linear tetramer was added dropwise to a solution of [dimethylamino(triazolo[4,5-b]pyridin-3-yloxy)-methylidene]-dimethylazanium hexafluoro-phosphate (HATU, 1.50 equiv.) and DIPEA (8.00 equiv.) in DMF. The mixture was stirred overnight at 21 °C, and the solvent was removed under reduced pressure. The residue was purified via preparative reversed-phase HPLC (Puriflash™ 4125, Interchim, Montluçon, France).

**Crystal structure determination:** The single-crystal X-ray diffraction studies were carried out on a Bruker D8 Venture diffractometer (Bruker Corporation, Billerica, MA, USA) with a PhotonII detector at 123(2) K, 173(2) K, or 298(2) K using Cu-Kα radiation (*λ* = 1.54178 Å). Dual space methods (SHELXT) [109] were used for structure solution, and refinement was carried out using SHELXL (full-matrix least-squares on *F^2^*) [110]. Hydrogen atoms were localized by difference electron density determination and refined using a riding model (H(N, O) free). Semi-empirical absorption corrections were applied. For **7a** and **8e,** extinction corrections were applied. The absolute configuration was determined for all structures refinement of Parsons’ x-parameter [111]. For disorder, restraints, constraints, and SQUEEZE, see the corresponding cif-files for details. CCDC 2059042 (**7a**), 2059043 (**7f**), 2059044 (**7e**), 2059045 (**8f**), 2059047 (**7h**), 2059048 (**8e**), and 2059049 (**7b**) contain the supplementary crystallographic data for this paper. These data can be obtained free of charge from The Cambridge Crystallographic Data Centre via www.ccdc.cam.ac.uk/data_request/cif (Cambridge, UK, (accessed on 14 May 2021)). More details on the single-crystal X-ray diffraction studies can be found in the supplementary information.

**NMR measurements:** NMR spectra were recorded at 25 °C on an Avance 300 (Bruker BioSpin, Rheinstetten, Germany) and a Bruker Avance 500 spectrometer. Additional NMR spectra of peptide-peptoid hybrid **9a** were recorded at 30 °C on a 600 MHz Avance III spectrometer with a TCI cryo-probe head (Bruker BioSpin, Rheinstetten, Germany). More details on the NMR measurements can be found in the supplemental information.

## 4. Conclusions

The three different classes of tetrameric cyclic peptide-peptoid hybrids presented here will pave the way to further research on this promising class of compounds. All macrocycles were designed to resemble the fungal metabolite apicidin (**1**) but without the characteristic Aoda side chain, critical for its literature known HDAC inhibitor activity [15]. The cyclic tetramers are accessible in moderate yields by combining different solid-phase techniques followed by ring closure in solution.

Several studies had previously shown that cyclic tetramers might adopt multiple conformations in solution, especially interchanging *cis* and *trans* peptide bonds [7,11,70]. The active conformation of biologically potent molecules in solution may be selected in reality from various interconverting conformers. The stability of the single conformers depends on intra- as well as intermolecular interactions [72]. The conversion rate between these conformers is quite high, making it difficult to identify every isomer formed [71,72].

Our X-ray and NMR measurements revealed the formation of different isomers in solid and liquid states for the cyclic tetramers presented. The amount of conformational variability depended on the number of incorporated peptoid units. Solution state NMR spectroscopy indicated different conformers for all compounds that exchanged partially within the NMR time scale. Especially for macrocycles with no or one peptoid monomer, multiple signal sets were detected. The incorporation of two peptoid monomers led to the stabilization of one dominant isomer.

Crystallographically detected conformers differed only in details concerning the backbone structure of the cyclic ring. Tetrapeptide conformers varied slightly in their dihedral angles but showed the same *cis*-*trans* sequence. The incorporation of one peptoid monomer did not change this *cis*-*trans* arrangement. The insertion of two peptoid monomers significantly affected the overall conformation. Instead of one, two *cis*-bonds were detected in the resulting macrocycles, indicating that the amount of peptoid monomers influences the spatial structure of peptide-peptoid hybrids.

With the structural information now in hand, biological targets can be identified, and, thanks to the modular approach, highly specific hybrids can be easily synthesized. These new molecules will find application in biochemical and medical research and help elucidate and sustain life’s complexity. We will continue our work on the activity of our macrocycles towards the Wnt/*β*-catenin signaling pathway. So far, we have found some hybrids with inhibition constants in the range of the model structure apicidin (**1**, data not shown). The structural investigations reported herein will help us design different potent inhibitors of the Wnt/*β*-catenin pathway, a signaling cascade involved in embryogenesis and homeostasis, and different diseases such as cancer or neurodegenerative disorders [112,113,114,115,116,117,118].

## Figures and Tables

**Table 1:** Cyclic tetrapeptides of general structure **7** and their respective yields over ten or eleven reaction steps.

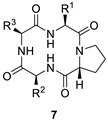				
Macrocycle	R^1^	R^2^	R^3^	Yield
**7a**	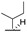	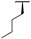	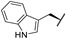	22%
**7b**	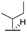	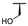	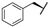	46% ^[a]^
**7c**	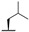	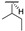	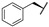	57%
**7d**	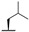	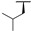	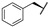	44%
**7e**	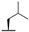	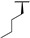	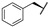	56%
**7f**	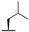	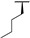	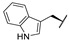	36%
**7g**	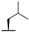	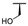	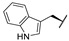	22% ^[a]^
**7h**	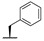	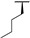	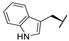	38%
**7i**	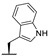	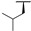	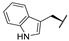	54%

^[a]^ Yield over eleven reaction steps.

**Table 2:** Cyclic tetramers of general structure **8** and their respective yields over ten reaction steps.

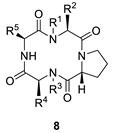						
Macrocycle	R^1^	R^2^	R^3^	R^4^	R^5^	Yield
**8a**	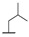	H	H	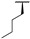	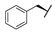	28%
**8b**	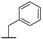	H	H	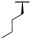	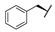	8.4%
**8c**	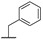	H	H	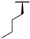	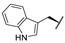	21%
**8d**	H	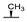	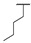	H	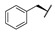	28%
**8e**	H	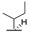	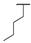	H	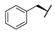	29%
**8f**	H	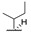	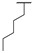	H	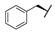	52%
**8g**	H	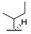	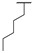	H	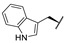	52%
**8h**	H	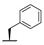	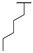	H	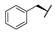	39%
**8i**	H	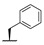	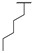	H	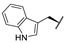	18%

**Table 3:** Cyclic tetramers of general structure **9** and their respective yields over ten reaction steps.

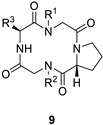				
Macrocycle	R^1^	R^2^	R^3^	Yield
**9a**	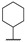	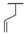	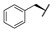	50%
**9b**	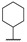	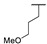	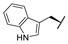	14%
**9c**	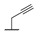	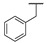	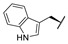	18%
**9d**	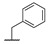	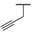	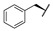	15%
**9e**	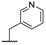	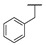	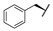	15%
**9f**	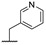	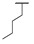	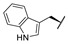	28%
**9g**	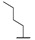	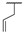	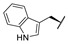	34%
**9h**	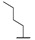	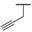	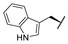	20%
**9i**	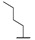	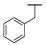	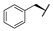	19%

**Table 4:** Dihedral angles of the independent structures of macrocycles **7a**, **7b**, **7e**, **7f** and **7h** determined via X-ray diffraction measurements.

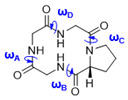					
Macrocycle	Structure	ω_A_	ω_B_	ω_C_	ω_D_
**7a**	I	155.7°	171.1°	5.2°	−167.6°
**7b**	I	159.0°	171.5°	6.5°	−172.7°
**7e**	I	157.3°	170.1°	12.1°	−173.3°
	II	158.1°	169.5°	6.5°	−172.4°
	III	158.5°	171.8°	13.1°	−175.6°
**7f**	I	160.4°	167.9°	−3.7°	−172.6°
	II	160.8°	167.4°	13.6°	−174.4°
	III	163.3°	166.8°	9.8°	−174.8
	IV	165.5°	163.7°	14.6°	−177.6°
	V	165.3°	163.9°	10.9°	−177.0°
**7h**	I	157.9°	169.0°	5.0°	−170.6°
	II	157.1°	169.6°	−6.9°	−168.0°
	III	153.1°	169.6°	13.5°	−172.7°
	IV	155.3°	171.0°	3.1°	−165.6°

**Table 5:** Molecular structures of the cyclic hybrids **8e** and **8f** and their dihedral angles determined via X-ray diffraction measurements.

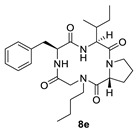		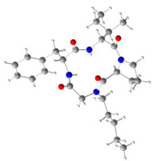		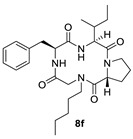		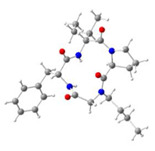	
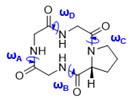							
Macrocycle	ω_A_		ω_B_		ω_C_		ω_D_
**8e**	155.2°		169.4°		12.2°		−173.0°
**8f**	155.3°		170.7°		13.5°		−172.4°

**Table 6:** Dihedral angles and molecular solution-state structure of the macrocycle **9a** determined via NMR measurements.

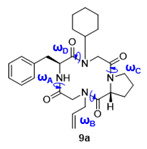			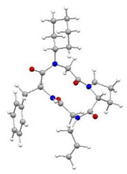		
Macrocycle	ω_A_	ω_B_		ω_C_	ω_D_
9a	−179.3°	14.8°		−177.1°	−11.2°

## Data Availability

The data presented in this study are openly available in the Chemotion repository: www.chemotion-repository.net (accessed on 14 May 2021).

## Supplementary Materials

The following are available online: synthetic procedure, crystallographic, and NMR data.

## Sample Availability

The molecules are deposited at the Compound Platform of the KIT. Selected molecules are available on request.
