# Supplementary material for: Macrocyclic Tetramers—Structural Investigation of Peptide-Peptoid Hybrids
Source: Molecules. 2021 Jul 28;26(15):4548. doi: 10.3390/molecules26154548 (PMC8348019; doi:10.3390/molecules26154548)
Supplement: Supplementary file 1 [file molecules-26-04548-s001.zip › molecules-1311356-supplementary.pdf]

Macrocyclic tetramers – structural investigation of peptide-peptoid hybrids

Claudine N. Herlan, Anna Sonnefeld, Thomas Gloge, Julian Brückel, Luisa C. Schlee, Claudia Muhle-Goll, Martin Nieger and Stefan Bräse

SUPPORTING INFORMATION

General experimental details

High-performance liquid chromatography (HPLC)

Reversed-phase analytical HPLC was performed on an Agilent Series 1100 equipped with a G1322A degasser, a G1311A pump, a G1313A autosampler, a G1316A oven, and a G1315B diode array detector (DAD). Alternatively, the HPLC system Thermofisher Ultimate 3000_1, equipped with an LPG-3400SD pump, a WPS-3000 autosampler, a TCC-3000 oven, and a DAD-3000 diode array detector was used. As a stationary phase, a VDSpher® C18-M-SE column (5.00 µm, 250 × 4.00 mm, VDS Optilab) was applied. As the mobile phase, a linear gradient of 5–95% acetonitrile in double-distilled water with 0.1% trifluoroacetic acid (TFA) with a flow rate of 1 mL/min over 30 min (unless stated otherwise) was used. Purity was calculated by integration of the detected signals at 218 nm.

Reversed-phase preparative HPLC was performed using the INTERCHIM HPLC system Puriflash™ 4125, equipped with the software InterSoft® V5.1.08. As a stationary phase, a VDSpher® C_{18}-M-SE precolumn (10 µm, 40 x 16 mm, VDS Optilab), followed by a VDSpher® C_{18}-M-SE column (10 µm, 250 x 20 mm, VDS Optilab) was used. As a mobile phase, a gradient of acetonitrile in double-distilled water with a 0.1% TFA with a 15 mL/min flow rate was used. The method (gradient, duration) was adjusted to each sample. Detection was carried out with a UV diode array detector in a spectral range of λ = 200–600 nm. Fractions absorbing at 218 nm, 256 nm or 280 nm were isolated.

Matrix Assisted Laser Desorption Ionization Time of Flight Mass Spectrometry (MALDI-TOF MS)

MALDI-TOF mass spectra were measured on a SHIMADZU BIOTECH Axima Confidence (model TO-6071R00) spectrometer, equipped with the software Shimadzu Biotech Launchpad™ (version 2.9.3.20110624). For desorption, a nitrogen laser (λ = 337 nm) was used. The samples were spotted on a SHIMADZU Kratos analytical standard stainless steel target (DE1580TA) with 386 spots. As matrix, a commercially available 1:1 mixture of 2,5-dihydroxybenzoic acid (DHB) and α-cyano-4-hydroxycinnamic acid (CHCA) (Universal MALDI matrix from SIGMA-ALDRICH®) was used as a saturated solution in 50% acetonitrile in double-distilled water was used. For every spectrum, the samples were shot around 100 times with a frequency of 50 Hz. The protonated molecule ion is expressed as $[M+H]^+$, pseudo-molecule ions as $[M+Na]^+$ and $[M+K]^+$, respectively.

Nuclear Magnetic Resonance Spectroscopy (NMR)

NMR spectra were recorded at 25 °C on a BRUKER Avance 300 (300 MHz (^{1}H), 75 MHz (^{13}C)) and a BRUKER Avance DRX 500 (500 MHz (^{1}H), 125 MHz (^{13}C)) spectrometer. Additional NMR spectra of peptide-peptoid hybrid **9a** were recorded at 30 °C on a 600 MHz Avance III spectrometer with a TCI cryo-probehead (Bruker BioSpin, Germany). Deuterated solvents were purchased from EURISOTOP. Chemical shifts (δ) are expressed in parts per million (ppm). All spectra are referenced to the signals of the residual protons of the solvents acetonitrile-d_3 (1.94 ppm (^{1}H), 118.3 ppm (^{13}C)), chloroform-d_1 (7.26 ppm (^{1}H), 77.2 ppm (^{13}C)), dimethylsulfoxide-d_6 (2.50 ppm (^{1}H), 39.5 ppm (^{13}C)) or methanol-d_4 (3.31 ppm (^{1}H), 49.0 ppm (^{13}C)) as an internal standard. The spectra were analyzed according to first order. Coupling constants (J) are given in Hertz (Hz). Multiplicities of signals are described as follows: s = singlet, d = doublet, t = triplet, q = quartet, quin = quintet, m = multiplet. Abbreviations for signal assignments include H_{Ar} = aromatic proton, C_{Ar} = aromatic carbon.

^{1}H and ^{13}C signals for peptide-peptoid hybrids **9a** were assigned with the help of multiplicity-edited ^{1}H-^{13}C-HSQC (Heteronuclear Single Quantum Coherence), HMBC (Heteronuclear Multiple Bond Correlation), and ^{1}H-^{1}H TOCSY (Total Correlation Spectroscopy), COSY (Correlation Spectroscopy) and NOESY (Nuclear Overhauser Enhancement Spectroscopy) experiments. Internuclear distances and residual dipolar couplings (RDCs) for **9a** were determined by evaluation of NOESY, CLIP-HSQC (Clean Inphase HSQC) [1] and P.E.HSQC [2] experiments. Baseline correction was carried out for all listed spectra. ^{1}H and ^{13}C signals for the remaining macrocycles were assigned with the help of ^{1}H-^{13}C-HSQC and ^{1}H-^{1}H-COSY experiments. Phase resolved ^{1}H-^{13}C-HSQC was used for the assignment of ^{13}C signals. Primary as well as tertiary carbon atoms are indicated by a "+", secondary ones are marked with a "–", whilst quaternary carbons are indicated by "C_q".

Table S1: Acquisition and processing parameters for the spectra recorded of **9a**. The pulse sequence for each experiment (exp.) is given. TD(F2) and TD(F1) are the number of points recorded in the respective dimension. SI(F2) and SI(F1) are the number of points the spectra were processed with. The window functions (WF) are given, as well as whether linear forward prediction (LPfr) was used.

exp.	pulse sequence	TD(F2)	TD(F1)	SI(F2)	SI(F1)	WF(F2)	WF(F1)	LPfr
**edHSQC**	hsqcedetgpsisp2.4	3k	2k	8k	4k	QSINE	QSINE	-
**HMBC**	hmbcetgpndsp	16k	1k	32k	4k	QSINE	QSINE	-
**^{1}H-^{1}H TOCSY**	dipsigpphzs	16k	1k	32k	2k	QSINE	QSINE	-
**COSY**	cosygpmfqf	16k	1k	16k	2k	TRAF	QSINE	-
**NOESY**	noesygpphzs	16k	1k	32k	2k	QSINE	QSINE	-
**CLIP-HSQC**	patz_gk_clipHSQC.SP	16k	1k	65k	4k	TRAF	QSINE	F2, F1
**P.E.HSQC**	patz_pehsqcSP_0.1	4k	2k	65k	8k	TRAF	QSINE	F2, F1

Crystal Structure Determination

The single-crystal X-ray diffraction studies were carried out on a Bruker D8 Venture diffractometer with a PhotonII detector at 123(2) K, 173(2) K or 298(2) K using Cu-Kα radiation (λ = 1.54178 Å). Dual space methods (SHELXT) [3] were used for structure solution and refinement was carried out using SHELXL (full-matrix least-squares on F^2) [4]. Hydrogen atoms were localized by difference electron density determination and refined using a riding model (H(N, O) free). Semi-empirical absorption corrections were applied. For **7a** and **8e** extinction corrections were applied. The absolute configuration was determined for all structures by refinement of Parsons' x-parameter [5]. For disorder, restraints, constraints and SQUEEZE see the corresponding cif-files for details.

7a: colourless crystals, $C_{28}H_{39}N_5O_4 \cdot C_2H_3N$, M_r = 550.69, crystal size 0.20 × 0.16 × 0.08 mm, hexagonal, space group *P6₅* (No. 170), a = 13.9920(3) Å, c = 26.7236(6) Å, V = 4530.9(2) Å^3, Z = 6, ρ = 1.211 Mg/m^{-3}, μ(Cu-K$_\alpha$) = 0.66 mm^{-1}, F(000) = 1776, T = 123(2) K, $2\theta_{max}$ = 144.6°, 86018 reflections, of which 5895 were independent (R_{int} = 0.026), 375 parameters, 14 restraints, R_1 = 0.029 (for 5876 I > 2σ(I)), wR_2 = 0.076 (all data), S = 1.09, largest diff. peak / hole = 0.21 / -0.26 e Å^{-3}, x = 0.01(3).

7b: colourless crystals, $C_{23}H_{32}N_4O_5 \cdot CH_4O$, M_r = 476.57, crystal size 0.18 × 0.06 × 0.03 mm, hexagonal, space group $P6_5$ (No. 170), a = 12.5370(3) Å, c = 27.1513(8) Å, V = 3695.8(2) Å^3, Z = 6, ρ = 1.285 Mg/m^{-3}, μ(Cu-K$_\alpha$) = 0.76 mm^{-1}, F(000) = 1536, T = 173(2) K, $2\theta_{max}$ = 144.4°, 47030 reflections, of which 4848 were independent (R_{int} = 0.034), 323 parameters, 11 restraints, R_1 = 0.041 (for 4772 I > 2σ(I)), wR_2 = 0.111 (all data), S = 1.08, largest diff. peak / hole = 0.98 (in solvent methanol) / -0.52 e Å^{-3}, x = -0.06(5).

7e: colourless crystals, $C_{26}H_{38}N_4O_4 \cdot 4/9\ H_2O$, M_r = 478.61, crystal size 0.18 × 0.06 × 0.03 mm, hexagonal, space group $P6_5$ (No. 170), a = 22.3350(4) Å, c = 27.1084(6) Å, V = 11711,3(5) Å^3, Z = 18, ρ = 1.222 Mg/m^{-3}, μ(Cu-K$_\alpha$) = 0.68 mm^{-1}, F(000) = 4652, T = 123(2) K, $2\theta_{max}$ = 144.4°, 145454 reflections, of which 15391 were independent (R_{int} = 0.061), 966 parameters, 778 restraints (general RIGU restraint), R_1 = 0.032 (for 14921 I > 2σ(I)), wR_2 = 0.077 (all data), S = 1.05, largest diff. peak / hole = 0.20 / -0.20 e Å^{-3}, x = 0.05(7). Refined as a 2-component twin (BASF = 0.227(1)). One water molecule (O1D) is a diffuse disordered water atom (s.o.f. = 1/3, with 2 water molecules in the unit cell).

7f: colourless crystals, $C_{28}H_{39}N_5O_4$, M_r = 509.64, crystal size 0.36 × 0.20 × 0.08 mm, orthorhombic, space group $P2_12_12_1$ (No. 19), a = 1§4.0190(3) Å, b = 23.5365(6) Å, c = 42.2193(10) Å, V = 13930.6(6) Å^3, Z = 20, ρ = 1.215 Mg/m^{-3}, μ(Cu-K$_\alpha$) = 0.66 mm^{-1}, F(000) = 5480, T = 123(2) K, $2\theta_{max}$ = 144.4°, 143273 reflections, of which 27390 were independent (R_{int} = 0.030), 1721 parameters, 85 restraints, R_1 = 0.030 (for 26747 I > 2σ(I)), wR_2 = 0.078 (all data), S = 1.03, largest diff. peak / hole = 0.40 / -0.29 e Å^{-3}, x = -0.05(2). In one molecule one n-butyl moiety is disordered.

7h: colourless crystals, $C_{31}H_{37}N_5O_4 \cdot H_2O$, M_r = 561.67, crystal size 0.40 × 0.20 × 0.10 mm, triclinic, space group *P1* (No. 1), a = 12.3164(5) Å, b = 15.1887(7) Å, c = 19.3159(8) Å, α = 68.750(2)°, β = 86.120(2)°, γ = 66.605(2)°, V = 3078.6(2) Å^3, Z = 4, ρ = 1.212 Mg/m^{-3}, μ(Cu-K$_\alpha$) = 0.68 mm^{-1}, F(000) = 1200, T = 298(2) K, $2\theta_{max}$ = 145.2°, 82609 reflections, of which 23152 were independent (R_{int} = 0.027), 1475 parameters, 2861 restraints, R_1 = 0.041 (for 22324 I > 2σ(I)), wR_2 = 0.116 (all data), S = 1.04, largest diff. peak / hole = 0.42 / -0.25 e Å^{-3}, x = -0.02(3). In the 4th molecule the *n*-butyl and the pyrrolidine moieties are disordered. Refinement with the listed atoms show residual electron density due to one disordered water solvent molecule (in the 2nd void), which could not be refined with split atoms. Therefore the option "SQUEEZE" of the program package PLATON [6, 7] was used to create a hkl file taking into account the residual electron density in the void areas. Therefore, the atoms list and unit card do not agree.

8e: colourless crystals, $C_{26}H_{38}N_4O_4 \cdot 2\ H_2O$, M_r = 506.63, crystal size 0.18 × 0.06 × 0.03 mm, orthorhombic, space group $P2_12_12_1$ (No. 19), a = 6.2879(2) Å, b = 17.9952(5) Å, c = 24.3575(6) Å, V = 2756.10(13) Å^3, Z = 4, ρ = 1.221 Mg/m^{-3}, μ(Cu-K$_\alpha$) = 0.71 mm^{-1}, F(000) = 1096, T = 173(2) K, $2\theta_{max}$ = 144.6°, 26303 reflections, of which 5433 were independent (R_{int} = 0.027), 344 parameters, 8 restraints, R_1 = 0.027 (for 5293 I > 2σ(I)), wR_2 = 0.066 (all data), S = 1.06, largest diff. peak / hole = 0.18 / -0.14 e Å^{-3}, x = 0.01(5).

8f: colourless crystals, $C_{27}H_{40}N_4O_2 \cdot 2\,H_2O$, M_r = 520.66, crystal size 0.20 × 0.06 × 0.02 mm, orthorhombic, space group $P2_12_12_1$ (No. 19), a = 6.3195(2) Å, b = 17.9453(5) Å, c = 24.9421(7) Å, V = 2828.57(14) Å^3, Z = 4, ρ = 1.223 Mg/m^{-3}, μ(Cu-K$_\alpha$) = 0.70 mm^{-1}, F(000) = 1128, T = 123(2) K, $2\theta_{max}$ = 144.4°, 25979 reflections, of which 5560 were independent (R_{int} = 0.037), 352 parameters, 8 restraints, R_1 = 0.032 (for 5342 I > 2σ(I)), wR_2 = 0.080 (all data), S = 1.05, largest diff. peak / hole = 0.24 / -0.18 e Å^{-3}, x = -0.02(7).

CCDC 2059042 (**7a**), 2059043 (**7f**), 2059044 (**7e**), 2059045 (**8f**), 2059047 (**7h**), 2059048 (**8e**), and 2059049 (**7b**) contain the supplementary crystallographic data for this paper. These data can be obtained free of charge from The Cambridge Crystallographic Data Centre *via* www.ccdc.cam.ac.uk/data_request/cif.

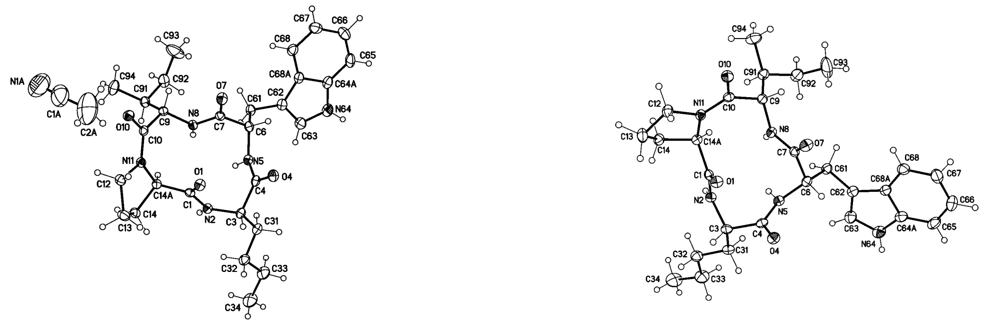

Figure S1: Molecular structure of macrocycle **7a**. Displacement parameters are drawn at 50% probability level. Right: solvent omitted for clarity.

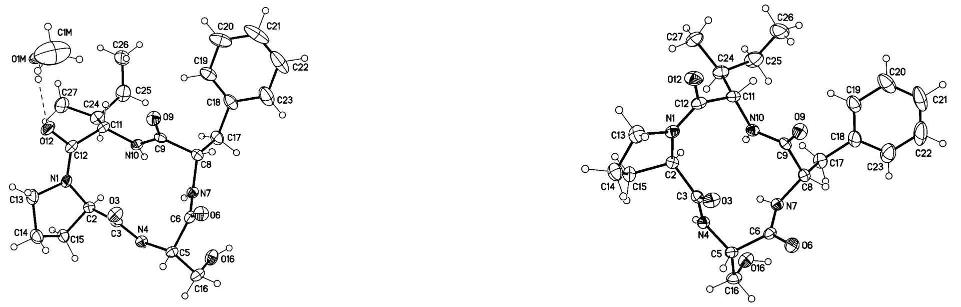

Figure S2: Molecular structure of macrocycle **7b**. Displacement parameters are drawn at 50% probability level. Right: solvent omitted for clarity.

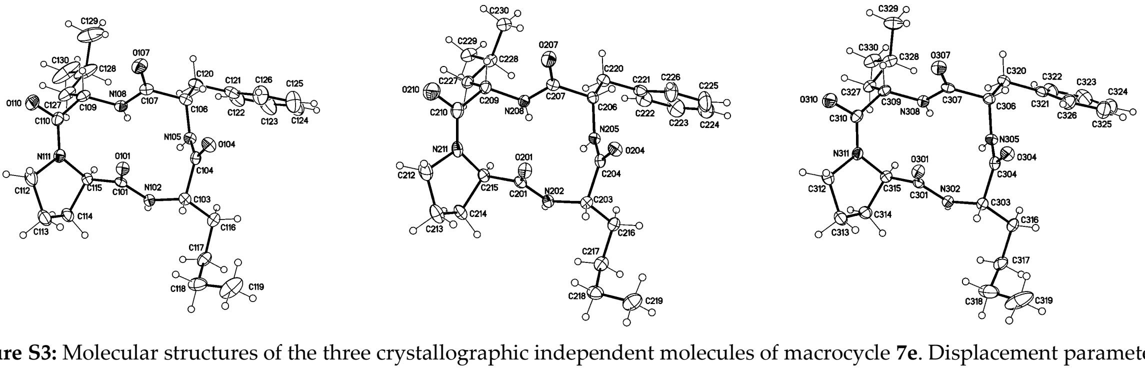

Figure S3: Molecular structures of the three crystallographic independent molecules of macrocycle **7e**. Displacement parameters are drawn at 50% probability level.

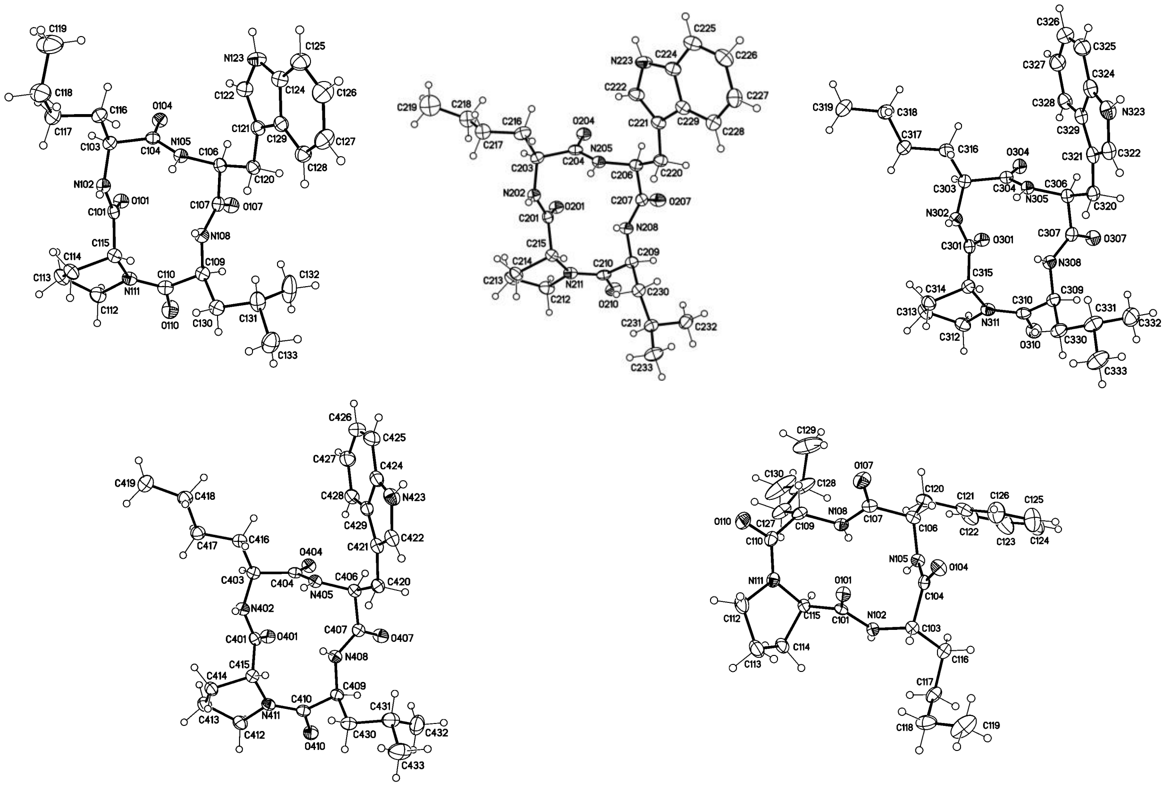

Figure S4: Molecular structures of the five crystallographic independent molecules of macrocycle **7f**. Displacement parameters are drawn at 50% probability level. Top center structure: minor disordered parts omitted for clarity.

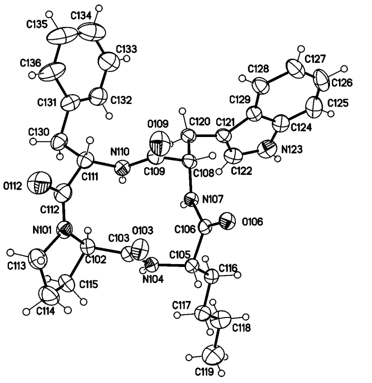

Figure S5: Molecular structure of one of the four crystallographic independent molecules of macrocycle **7h**. Displacement parameters are drawn at 30% probability level.

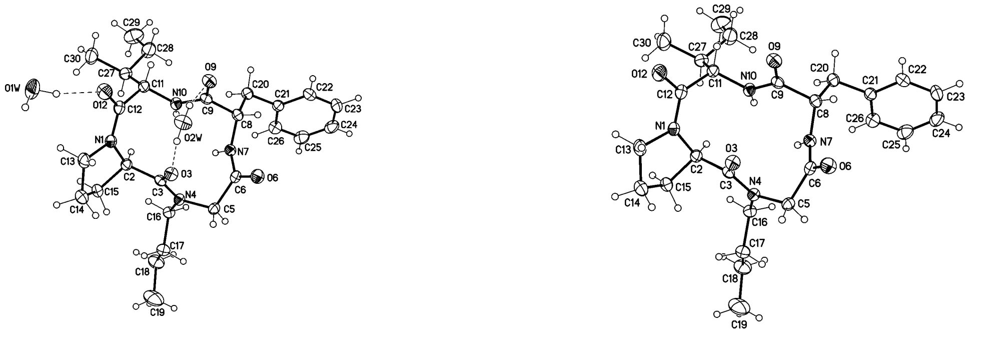

Figure S6: Molecular structure of macrocycle **8e**. Displacement parameters are drawn at 50% probability level. Right: solvent omitted for clarity.

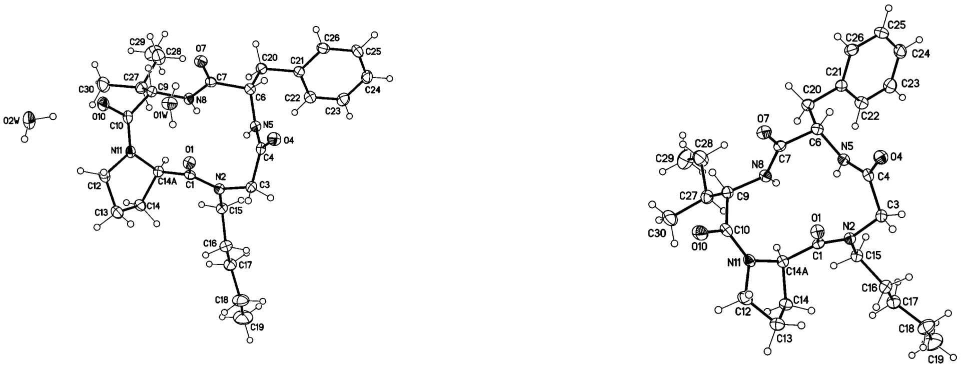

Figure S7: Molecular structure of macrocycle **8f**. Displacement parameters are drawn at 50% probability level. Right: solvent omitted for clarity.

General experimental procedures

Solvents and reagents were purchased from commercial sources and used without further purification. Water was deionized using a Elix® Essential 15 water purification system (Millipore®) and purified further with a Milli-Q Biocel system (Q-Gard® 1, Quantum® X, Millipore®) for chromatographical applications. Abbreviations are as follows: acetonitrile (ACN), ^{tert}butyloxycarbonyl (Boc), *N,N'*-diisopropylcarbodiimide (DIC), *N,N'*-diisopropylethylamine (DIPEA), dimethylformamide (DMF), fluorenylmethoxycarbonyl (Fmoc), [Dimethylamino(triazolo[4,5-b]pyridin-3-yloxy)methylidene]-dimethylazanium hexafluorophosphate (HATU), 1,1,1,3,3,3-hexafluoroisopropyl alcohol (HFIP), 1-hydroxybenzotriazole (HOBt), methylene chloride (DCM), *N*-methylpyrrolidone (NMP), trifluoroacetic acid (TFA).

Reagents and products were weighted on a SARTORIUS CP224S (d = 0.1 mg) or a RADWAG AS 220.X2 (d = 0.1 mg) scale.

Solvents were removed under reduced pressure on a rotary evaporator at 40–60 °C heating bath temperature. Aqueous solutions were deep-frozen and lyophilized under reduced pressure on a LDC-1 (Alpha 2–4) dry freezer (CHRIST).

Solid-phase synthesis [8, 9]

Solid-phase synthesis of linear precursors was performed in accordance with the published solid phase peptide synthesis [8] and the submonomer technique [9] in 6 mL plastic-fritted syringes (MULTISYNTHEC GmbH), closed with a plastic cap. As solid support, a 2-chlorotrityl chloride resin (CARBOLUTION, 1.60 mmol/g loading density, 100–200 mesh,) was used. Reaction steps were performed on a KS501 digital circular shaker (160–200 rpm, IKA-LABORTECHNIK) at 21 °C. Yields were calculated according to the resin loading value.

General Procedure (GP1) for the immobilization of an amino acid [8]

In a fritted syringe, the 2-chlorotrityl chloride resin (125 mg, 200 μmol, 1.60 mmol/mg loading density, 1.00 equiv.) was swollen in 2.50 mL of DCM for at least 30 min. After filtration, the respective Fmoc-protected amino acid (800 μmol, 4.00 equiv.) and DIPEA (139 μL, 103 mg, 800 μmol, 4.00 equiv.) were dissolved in 2 mL of NMP and added to the resin. The reaction mixture was incubated overnight at 21 °C. The solvent was removed by filtration and the resin was washed with 2 mL of DMF, methanol, DCM and DMF each.

Afterwards, the resin was incubated with 2.50 mL of a 20% solution of piperidine in DMF for 5 min. The solvent was removed and the procedure was repeated twice. The resin was washed as described.

General Procedure (GP2) for the immobilization of a peptoid monomer [9]

In a fritted syringe, the 2-chlorotrityl chloride resin (125 mg, 200 μmol, 1.60 mmol/mg loading density, 1.00 equiv.) was swollen in 2.50 mL of DCM for at least 30 min. After filtration, a 1 M solution of bromoacetic acid (222 mg, 1.60 mmol, 8.00 equiv.) and DIPEA (278 μL, 206 mg, 1.60 mmol, 8.00 equiv.) in DMF was added to the resin. The reaction mixture was incubated for 1 h at 21 °C. The solvent was removed my filtration and the resin was washed with 2 mL of DMF, methanol, DCM and DMF each.

Subsequently, a solution of the respective amine (1.60 mmol, 8.00 equiv.) in 2 mL of DMF was added and the mixture was shaken for 1 h at 21 °C. The solvent was removed and the resin was washed as described.

General Procedure (GP3) for the coupling of an amino acid [8]

The respective Fmoc-protected amino acid (800 μmol, 4.00 equiv.) and HOBt (123 mg, 800 μmol, 4.00 equiv.) were dissolved in 2 mL of NMP. DIC (125 μL, 101 mg, 800 μmol, 4.00 equiv.) was added and the resin was immediately incubated with the resulting solution for 4 h at 21 °C. The solvent was removed by filtration and the resin was washed as described.

Afterwards, the resin was incubated with 2.50 mL of a 20% solution of piperidine in DMF for 5 min. The solvent was removed and the procedure was repeated twice. The resin was washed as described.

General Procedure (GP4) for the coupling of a peptoid monomer [9]

DIC (250 µL, 202 mg, 1.60 mmol, 8.00 equiv.) was added to an 1 M solution of bromoacetic acid (222 mg, 1.60 mmol, 8.00 equiv.) in DMF. The solution was immediately added to the resin and shaken for 30 min at 21 °C. The solvent was removed by filtration and the resin was washed as described.

Subsequently, a solution of the respective amine (1.60 mmol, 8.00 equiv.) in 2 mL of DMF was added and the mixture was shaken for 1 h at 21 °C. The solvent was removed and the resin was washed as described.

General Procedure (GP5) for the cleavage of linear precursors

The resin was extensively washed with DCM. 2.50 mL of a solution of 33% HFIP in DCM was added and incubated twice for 1 h or rather overnight at 21 °C. The cleavage cocktail was collected and the resin was flushed with DCM. The solvent was removed under air stream. The residue was dissolved in acetonitrile and water, deep-frozen and lyophilized to give the crude linear precursor.

General Procedure (GP6) for the cyclization of linear precursors [10]

[Dimethylamino(triazolo[4,5-b]pyridin-3-yloxy)methylidene]dimethylazanium hexafluorophosphate (HATU, 28.5 mg, 75.0 µmol, 0.750 equiv.) was dissolved in 30 mL of DMF. In parallel, the linear precursor (100 µmol, 1,00 equiv.) was dissolved in 20 mL of DMF and DIPEA (103 mg, 139 µL, 800 µmol, 8.00 equiv) was added. 10 mL of this mixture were added to the HATU solution over a period of 6 h at 21 °C. Afterwards, additional 0.750 equiv. of HATU (28.5 mg, 75.0 µmol) were added to the reaction mixture in one portion. The remaining 10 mL of the linear precursor mixture were added over another period of 6 h. The reaction was stirred for 12 h at 21 °C and the solvent was removed under reduced pressure.

General Procedure (GP7) for the cleavage of Boc- and ^{tert}butyl-protecting groups

The residue was dissolved in 5 mL of a 95% solution of TFA in DCM and stirred for 1 h at 21 °C. The solvent was removed under air stream.

***cyclo*-(L-Ile-D-Pro-L-Nle-L-Trp) (7a)**

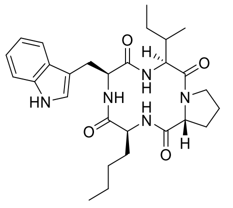

Fmoc-L-tryptophan (341 mg, 800 µmol, 4.00 equiv.) was immobilized in accordance with **GP1**. Subsequently, Fmoc-L-norleucine (283 mg, 800 µmol, 4.00 equiv.), Fmoc-D-proline (270 mg, 800 µmol, 4.00 equiv.) and Fmoc-L-isoleucine (283 mg, 800 µmol, 4.00 equiv.) were coupled following **GP3**. Cleavage was performed according to **GP5**. The crude linear precursor (53.0 mg, 100 µmol, 1.00 equiv.) was cyclized following **GP6**. After purification *via* preparative reversed phase HPLC (5–95% acetonitrile in water with 0.1% TFA in 45 min), the macrocycle was isolated as colorless solid (11.2 mg, 22.0 µmol, 22% over 10 steps).

^{1}**H-NMR** (500 MHz, chloroform-d_1, ppm): δ = 8.03 (s, 1H, trp-N*H*), 7.60 (d, *J* = 7.9 Hz, 1H, trp-C*H*), 7.43 (d, *J* = 7.9 Hz, 1H, ile-N$_\alpha$*H*), 7.35 (d, *J* = 8.2 Hz, 1H, trp-C*H*), 7.15–7.09 (m, 3H, 2 × trp-C*H*, nle-N$_\alpha$*H*), 7.04 (s, 1H, trp-CH), 6.46–6.41 (m, 1H, trp-N$_\alpha$*H*), 4.76 (dd, *J* = 7.9, 1.9 Hz, 1H, pro-C$_\alpha$*H*), 4.54 (t, *J* = 10.6 Hz, 1H, ile-C$_\alpha$*H*), 4.20–4.12 (m, 1H, nle-C$_\alpha$*H*), 4.05–3.98 (m, 1H, trp-C$_\alpha$*H*), 3.94–3.86 (m, 1H, pro-C$_\delta$*H*H), 3.82 (dd, *J* = 14.8, 9.7 Hz, 1H, trp-C*H*H), 3.59 (dd, *J* = 14.7, 7.2 Hz, 1H, trp-CH*H*), 3.54–3.46 (m, 1H, pro-C$_\delta$H*H*), 2.41–2.33 (m, 1H, pro-C$_\beta$*H*H), 2.31–2.21 (m, 1H, pro-C$_\beta$H*H*), 2.08–1.99 (m, 1H, ile-C*H*), 1.97–1.88 (m, 1H, pro-C$_\gamma$*H*H), 1.88–1.70 (m, 2H, nle-C$_\beta$*H*H, pro-C$_\gamma$H*H*), 1.63–1.53 (m, 2H, nle-C$_\beta$H*H*, nle-C$_\gamma$*H*H), 1.33–1.25 (m, 2H, ile-C*H*$_2$), 1.23–1.11 (m, 3H, nle-C$_\gamma$H*H*, nle-C$_\delta$*H*$_2$), 0.93–0.83 (m, 9H, 2 × ile-C*H*$_3$, nle-C*H*$_3$). – ^{13}**C-NMR** (125 MHz, chloroform-d_1, ppm): δ = 175.1 (C$_q$, *C*ON), 172.6 (C$_q$, 2 × *C*ON), 171.8 (C$_q$, *C*ON), 136.3 (C$_q$, trp-C$_{ar}$), 127.1 (C$_q$, trp-C$_{ar}$), 123.0 (+,trp-CH), 122.4 (+, trp-CH), 119.8 (+, trp-CH), 118.7 (+, trp-CH), 111.4 (+, trp-CH), 111.2 (C$_q$, trp-C$_{ar}$), 61.9 (+, trp-*C*$_\alpha$H), 58.1 (+, 2C, pro-*C*$_\alpha$H, ile-C$_\alpha$H), 54.4 (+, nle-C$_\alpha$H), 47.2 (–, pro-C$_\delta$H$_2$), 34.0 (+, ile-CH), 28.9 (–, nle-C$_\beta$H$_2$), 27.8 (–, nle-C$_\delta$H$_2$), 25.4 (–, trp-CH$_2$), 25.1 (–, 2C, pro-*C*$_\beta$H$_2$, pro-*C*$_\gamma$H$_2$), 24.9 (–, nle-*C*$_\gamma$H$_2$), 22.5 (–, ile-CH$_2$), 15.9 (+, nle-CH$_3$), 14.0 (+, ile-CH$_3$), 10.8 (+, ile-CH$_3$). – **Analytical HPLC** (5–95% acetonitrile + 0.1% TFA in 30 min, detection at 218 nm): t$_{Ret}$ = 10.9 min (>99%). – **MS** (m/z, MALDI-TOF): 510 [M+H]$^+$, 532 [M+Na]$^+$, 548 [M+K]$^+$.

***cyclo*-(L-Ile-D-Pro-L-Ser-L-Phe) (7b)**

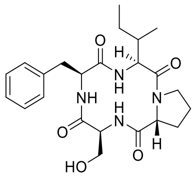

Fmoc-L-phenylalanine (310 mg, 800 µmol, 4.00 equiv.) was immobilized in accordance with **GP1**. Subsequently, *O*-^{tert}Butyl-Fmoc-L-serine (307 mg, 800 µmol, 4.00 equiv.), Fmoc-D-proline (270 mg, 800 µmol, 4.00 equiv.) and Fmoc-L-isoleucine (283 mg, 800 µmol, 4.00 equiv.) were coupled following **GP3**. Cleavage was performed according to **GP5**. The crude linear precursor (56.2 mg, 100 µmol, 1.00 equiv.) was cyclized following **GP6** and deprotected in accordance with **GP7**. After purification *via* preparative reversed phase HPLC (5–95% acetonitrile in water with 0.1% TFA in 45 min), the macrocycle was isolated as colorless solid (25.0 mg, 56.2 µmol, 46% over 11 steps).

The NMR spectra contain multiple signal sets. Chemical shifts of the most prominent isomers are given. Isomer "(1)" is represented in about twice the amount of isomer "(2)". ^{1}**H-NMR** (500 MHz, DMSO-d_6, ppm): δ = 7.88–7.79 (m, 2H, ser(1)-N$_\alpha$*H*, ile(2)-N$_\alpha$*H*), 7.57 (d, *J* = 9.1 Hz, 1H, phe(2)-N$_\alpha$*H*), 7.46–7.39 (m, 2H, ile(1)-N$_\alpha$*H*, phe(1)-N$_\alpha$*H*), 7.33–7.16 (m, 5H, 5 × phe(1)-C*H*, 5 × phe(2)-C*H*, ser(2)-N$_\alpha$*H*), 4.79 (d, *J* = 7.4 Hz, 1H, pro(2)-C$_\alpha$*H*), 4.74 (d, *J* = 7.7 Hz, 1H, pro(1)-C$_\alpha$*H*), 4.42–

4.34 (m, 1H, phe(1)-$C_{\alpha}H$), 4.33–4.15 (m, 3H, ile(2)-$C_{\alpha}H$, phe(2)-$C_{\alpha}H$, ser(2)-$C_{\alpha}H$), 4.13–4.06 (m, 1H, ile(1)-$C_{\alpha}H$), 3.91–3.84 (m, 1H, ser(1)-$C_{\alpha}H$), 3.63–3.59 (m, 3H, ser(1)-CH_2, ser(2)-CH_2), 3.53–3.44 (m, 2H, pro(2)-$C_{\delta}H_2$), 3.41–3.32 (m, 2H, pro(1)-$C_{\delta}H_2$), 3.17 (dd, *J* = 13.6, 8.6 Hz, 1H, phe(2)-C*H*H), 3.04 (dd, *J* = 13.6, 8.6 Hz, 1H, phe(2)-CH*H*), 2.92–2.82 (m, 2H, phe(1)-CH_2), 2.24–2.12 (m 2H, ile(2)-C*H*H, pro(1)-C_{β}*H*H), 2.10–1.96 (m, 3H, ile(2)-C*H*, pro(1)-C_{β}H*H*, pro(2)-C_{β}*H*H), 1.93–1.75 (m, 5H, ile(1)-C*H*, pro(2)-C_{β}H*H*, pro(1)-C_{γ}*H*H, pro(2)-$C_{\gamma}H_2$), 1.73–1.64 (m, 1H, ile(2)-CH*H*), 1.61–1.46 (m, 1H, ile(1)-C*H*H, pro(1)-C_{γ}H*H*), 1.23–1.13 (m, 1H, ile(1)-CH*H*), 0.89–0.79 (m, 12H, 2 × ile(1)-CH_3, 2 × ile(2)-CH_3). – [13]**C-NMR** (125 MHz, DMSO-d_6, ppm): δ = 173.2 (C_q, (2)-CON), 172.9 (C_q, (2)-CON), 172.6 (C_q, (1)-CON), 172.4 (C_q, (2)-CON), 171.3 (C_q, 2 × (1)-CON), 171.0 (C_q, (2)-CON), 168.4 (C_q, (1)-CON), 137.5 (C_q, phe(1)-C_{ar}), 137.4 (C_q, phe(2)-C_{ar}), 129.0 (+, 2 × phe(2)-CH), 128.9 (+, 2 × phe(1)-CH), 128.2 (+, 2 × phe(1)-CH, 2 × phe(2)-CH), 126.5 (+, phe(2)-CH), 126.4 (+, phe(1)-CH), 59.9 (+, ile(1)-C_{α}H), 59.8 (+, ser(1)-C_{α}H), 59.4 (–, ser(2)-CH_2), 59.0 (–, ser(1)-CH_2), 58.3 (+, pro(1)-C_{α}H), 58.1 (+, ile(2)-C_{α}H), 57.2 (+, pro(2)-C_{α}H), 56.6 (+, phe(1)-C_{α}H), 56.4 (+, ser(2)-C_{α}H), 56.3 (+, phe(2)-C_{α}H), 47.4 (–, pro(1)-$C_{\delta}H_2$), 46.0 (–, pro(2)-$C_{\delta}H_2$), 37.6 (–, phe(1)-CH_2), 35.0 (–, phe(2)-CH_2), 34.6 (+, ile(1)-CH), 33.8 (+, ile(2)-CH), 32.0 (–, pro(1)-$C_{\beta}H_2$, pro(2)-$C_{\beta}H_2$), 25.2 (–, ile(1)-CH_2), 24.7 (–, ile(2)-CH_2), 24.3 (–, pro(2)-$C_{\gamma}H_2$), 20.1 (–, pro(1)-$C_{\gamma}H_2$), 15.6 (+, ile(1)-CH_3), 15.3 (+, ile(2)-CH_3), 10.4 (+, ile(2)-CH_3), 9.60 (+, ile(1)-CH_3). – **Analytical HPLC** (5–95% acetonitrile + 0.1% TFA in 30 min, detection at 218 nm): t_{Ret} = 10.3 min (98%). – **MS** (m/z, MALDI-TOF): 445 $[M+H]^+$, 467 $[M+Na]^+$, 483 $[M+K]^+$.

***cyclo*-(L-Leu-D-Pro-L-Ile-L-Phe) (7c)**

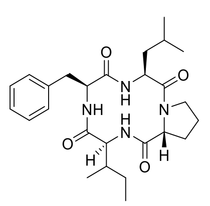

Fmoc-L-phenylalanine (310 mg, 800 μmol, 4.00 equiv.) was immobilized in accordance with **GP1**. Subsequently, Fmoc-L-isoleucine (283 mg, 800 μmol, 4.00 equiv.), Fmoc-D-proline (270 mg, 800 μmol, 4.00 equiv.) and Fmoc-L-leucine (283 mg, 800 μmol, 4.00 equiv.) were coupled following **GP3**. Cleavage was performed according to **GP5**. The crude linear precursor (49.0 mg, 100 μmol, 1.00 equiv.) was cyclized following **GP6**. After purification *via* preparative reversed phase HPLC (5–95% acetonitrile in water with 0.1% TFA in 45 min), the macrocycle was isolated as colorless solid (29.3 mg, 62.3 μmol, 57% over 10 steps).

The NMR spectra contain multiple signal sets. Chemical shifts of the most prominent isomers are given. Isomers "(1)" and "(2)" are represented in a similar amount. Isomer "(3)" represents a minor conformation. [1]**H-NMR** (500 MHz, DMSO-d_6, ppm): δ = 8.05 (d, *J* = 9.8 Hz, 1H, ile(2)-$N_{\alpha}H$), 7.91 (d, *J* = 8.8 Hz, 1H, phe(3)-$N_{\alpha}H$), 7.78 (d, *J* = 6.1 Hz, 1H, ile(1)-$N_{\alpha}H$), 7.62 (d, *J* = 10.3 Hz, 1H, leu(3)-$N_{\alpha}H$), 7.60 (d, *J* = 10.5 Hz, 1H, phe(2)-$N_{\alpha}H$), 7.46 (d, *J* = 10.6 Hz, 1H, phe(1)-$N_{\alpha}H$), 7.39 (d, *J* = 8.3 Hz, 1H, leu(1)-$N_{\alpha}H$), 7.33 (d, *J* = 11.0 Hz, 1H, leu(2)-$N_{\alpha}H$), 7.31–7.23 (m, 6H, 2 × phe(1)-C*H*, 2 × phe(2)-C*H*, 2 × phe(3)-C*H*), 7.22–7.13 (m, 9H, 3 × phe(1)-C*H*, 3 × phe(2)-C*H*, 3 × phe(3)-C*H*), 6.90 (d, *J* = 10.2 Hz, 1H, ile(3)-$N_{\alpha}H$), 4.81 (d, *J* = 7.4 Hz, 1H, pro(3)-$C_{\alpha}H$), 4.74 (q, *J* = 8.0 Hz, 1H, leu(3)-$C_{\alpha}H$), 4.61 (d, *J* = 7.9 Hz, 1H, pro(1)-$C_{\alpha}H$), 4.48–4.41 (m, 2H, leu(1)-$C_{\alpha}H$, leu(2)-$C_{\alpha}H$), 4.36 (td, *J* = 11.0, 4.8 Hz, 1H, phe(1)-$C_{\alpha}H$), 4.30 (dt, *J* = 11.4, 5.9 Hz, 1H, pro(2)-$C_{\alpha}H$), 4.17 (td, *J* = 1.0, 2.9 Hz, 1H, phe(2)-$C_{\alpha}H$), 4.13–4.07 (m, 1H, phe(3)-$C_{\alpha}H$), 3.87 (t, *J* = 11.0 Hz, 1H, ile(3)-$C_{\alpha}H$), 3.82 (t, *J* = 10.0 Hz, 1H, ile(2)-$C_{\alpha}H$), 3.53 (dd, *J* = 8.9, 5.9 Hz, 1H, ile(1)-$C_{\alpha}H$), 3.43 (dd, *J* = 13.9, 2.7 Hz, 1H, phe(2)-C*H*H), 3.39–3.29 (m, 6H, pro(1)-$C_{\delta}H_2$, pro(2)-$C_{\delta}H_2$, pro(3)-$C_{\delta}H_2$), 3.09–3.04 (m, 2H, phe(3)-CH_2), 2.92 (dd, *J* = 13.9, 5.1 Hz, 1H, phe(1)-C*H*H), 2.77 (dd, *J* = 13.9, 10.8 Hz, 1H, phe(1)-CH*H*), 2.60 (dd, *J* = 13.8, 11.5 Hz, 1H, phe(2)-CH*H*), 2.25–2.12 (m, 2H, pro(1)-C_{β}*H*H, pro(3)-C_{β}*H*H), 2.11–2.03 (m, 3H, pro(1)-C_{β}H*H*, pro(2)-C_{β}*H*H, pro(3)-C_{β}H*H*), 1.81–1.75 (m, 7H, leu(2)-C*H*H, pro(2)-C_{β}H*H*, pro(1)-C_{γ}*H*H, pro(2)-$C_{\gamma}H_2$, pro(3)-$C_{\gamma}H_2$), 1.69–1.60 (m, 8H, leu(1)-C*H*, leu(2)-C*H*, leu(3)-C*H*, ile(2)-C*H*, ile(3)-C*H*, leu(1)-C*H*H, leu(3)-CH_2), 1.59–1.53 (m, 5H, ile(1)-C*H*, ile(1)-C*H*H, ile(3)-CH_2, pro(1)-C_{γ}H*H*), 1.47–1.38 (m, 2H, leu(1)-CH*H*, leu(2)-CH*H*), 1.34–1.27 (m, 2H, ile(2)-CH_2), 1.14–1.07 (m, 1H, ile(1)-CH*H*), 0.97 (d, *J* = 6.4 Hz, 6H, ile(1)-CH_3, leu(2)-CH_3), 0.94 (d, *J* = 6.1 Hz, 3H, ile(3)-CH_3), 0.89–0.80 (m, 12H, 2 × ile(2)-CH_3, ile(3)-CH_3, leu(1)-CH_3, leu(3)-CH_3), 0.74–0.68 (m, 6H, ile(1)-CH_3, leu(2)-CH_3), 0.62 (d, *J* = 6.6 Hz, 3H, leu(3)-CH_3), 0.32 (d, *J* = 6.5 Hz, 3H, leu(1)-CH_3). – [13]**C-NMR** (125 MHz, DMSO-d_6, ppm): δ = 172.9 (C_q, (3)-CON), 172.4 (C_q, (1)-CON, (2)-CON), 172.3 (C_q, (2)-CON), 172.2 (C_q, (2)-CON), 172.1 (C_q, (3)-CON), 171.4 (C_q, (3)-CON), 171.4 (C_q, (1)-CON), 170.1 (C_q, (1)-CON), 169.6 (C_q, (2)-CON), 169.6 (C_q, (3)-CON), 169.6 (C_q, (1)-CON), 138.7 (C_q, phe(2)-C_{ar}), 137.5 (C_q, phe(1)-C_{ar}), 137.4 (C_q, phe(3)-C_{ar}), 129.0 (+, 2 × phe(3)-CH), 128.9 (+, 2 × phe(2)-CH), 128.8 (+, 2 × phe(1)-CH), 128.3 (+, 2 × phe(2)-CH), 128.2 (+, 2 × phe(3)-CH), 128.1 (+, 2 × phe(1)-CH), 126.5 (+, phe(3)-CH), 126.4 (+, phe(1)-CH), 126.2 (+, phe(2)-CH),62.6 (+, ile(1)-C_{α}H), 60.5 (+, ile(3)-C_{α}H), 58.5 (+, pro(1)-C_{α}H), 58.3 (+, phe(1)-C_{α}H), 58.2 (+, phe(2)-C_{α}H), 57.8 (+, leu(2)-C_{α}H), 57.3 (+, pro(3)-C_{α}H), 56.1 (+, phe(3)-C_{α}H), 55.3 (+, pro(2)-C_{α}H), 53.7 (+, leu(1)-C_{α}H), 51.4 (+, ile(2)-C_{α}H), 49.4 (+, leu(3)-C_{α}H), 48.0 (–, pro(2)-$C_{\delta}H_2$), 47.9 (–, pro(3)-$C_{\delta}H_2$), 47.7 (–, pro(1)-$C_{\delta}H_2$), 40.1 (–, leu(1)-CH_2), 38.4 (–, leu(3)-CH_2), 37.5 (–, leu(2)-CH_2), 37.1 (–, phe(1)-CH_2), 36.1 (–, phe(2)-CH_2), 34.9 (–, phe(3)-CH_2), 34.5 (+, ile(1)-CH), 33.6 (+, ile(2)-CH, ile(3)-CH), 32.5 (–, pro(1)-$C_{\beta}H_2$, pro(3)-$C_{\beta}H_2$), 32.2 (–, pro(2)-$C_{\beta}H_2$), 25.9 (–, ile(1)-CH_2, ile(3)-CH_2), 24.6 (+, leu(3)-CH), 24.4 (–, ile(2)-CH_2), 24.3 (+, leu(1)-CH), 24.0 (+, leu(2)-CH), 22.8 (+, ile(1)-CH_3), 22.7 (+, leu(2)-CH_3), 22.2 (+, ile(3)-CH_3), 21.2 (+, leu(1)-CH_3), 21.0 (+, ile(2)-CH_3, leu(3)-CH_3), 20.2 (–, pro(1)-$C_{\gamma}H_2$, pro(2)-$C_{\gamma}H_2$, pro(3)-$C_{\gamma}H_2$), 15.6 (+, leu(1)-CH_3), 14.9, (+, leu(2)-CH_3, leu(3)-CH_3), 10.9 (+, ile(1)-CH_3), 10.6 (+, ile(2)-CH_3), 10.0 (+,ile(3)-CH_3). – **Analytical HPLC** (5–95% acetonitrile + 0.1% TFA in 30 min, detection at 218 nm): t_{Ret} = 12.5 min (>99%). – **MS** (m/z, MALDI-TOF): 471 $[M+H]^+$, 493 $[M+Na]^+$, 509 $[M+K]^+$.

***cyclo*-(L-Leu-D-Pro-L-Ile-L-Phe) (7d)**

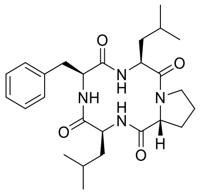

Fmoc-L-phenylalanine (310 mg, 800 µmol, 4.00 equiv.) was immobilized in accordance with **GP1**. Subsequently, Fmoc-L-leucine (283 mg, 800 µmol, 4.00 equiv.), Fmoc-D-proline (270 mg, 800 µmol, 4.00 equiv.) and Fmoc-L-leucine (283 mg, 800 µmol, 4.00 equiv.) were coupled following **GP3**. Cleavage was performed according to **GP5**. The crude linear precursor (49.0 mg, 100 µmol, 1.00 equiv.) was cyclized following **GP6**. After purification *via* preparative reversed phase HPLC (5–95% acetonitrile in water with 0.1% TFA in 45 min), the macrocycle was isolated as colorless solid (22.8 mg, 48.5 µmol, 44% over 10 steps).

The NMR spectra contain multiple signal sets. Chemical shifts of the most prominent isomers are given. Isomers "(1)" and "(2)" are represented in a similar amount. Isomer "(3)" represents a minor conformation. **^{1}H-NMR** (500 MHz, DMSO-d_6, ppm): δ = 8.10 (d, *J* = 9.8 Hz, 1H, phe(2)-N$_\alpha$*H*), 7.87–7.78 (m, 2H, leu(1)-N$_\alpha$*H*, phe(3)-N$_\alpha$*H*), 7.63 (d, *J* = 10.0 Hz, 1H, leu(3)-N$_\alpha$*H*), 7.57 (d, *J* = 10.3 Hz, 1H, leu(1)-N$_\alpha$*H*), 7.46 (d, *J* = 10.6 Hz, 1H, phe(1)-N$_\alpha$*H*), 7.40 (d, *J* = 8.2 Hz, 1H, leu(2)-N$_\alpha$*H*), 7.31–7.14 (m, 16H, leu(2)-N$_\alpha$*H*, 5 × phe(1)-C*H*, 5 × phe(2)-C*H*, 5 × phe(3)-C*H*), 6.92 (d, *J* = 10.1 Hz, 1H, leu(3)-N$_\alpha$*H*), 4.77 (d, *J* = 7.4 Hz, 1H, pro(3)-C$_\alpha$*H*), 4.72 (q, *J* = 8.9 Hz, 1H, leu(3)-C$_\alpha$*H*), 4.59 (d, *J* = 7.8 Hz, 1H, pro(1)-C$_\alpha$*H*), 4.46–4.40 (m, 1H, leu(2)-C$_\alpha$*H*), 4.39 (d, *J* = 8.7 Hz, 1H, pro(2)-C$_\alpha$*H*), 4.36–4.25 (m, 3H, phe(1)-C$_\alpha$*H*, leu(2)-C$_\alpha$*H*, leu(3)-C$_\alpha$*H*), 4.25–4.16 (m, 2H, leu(1)-C$_\alpha$*H*, phe(2)-C$_\alpha$*H*), 4.15–4.10 (m, 1H, phe(3)-C$_\alpha$*H*), 3.79–3.71 (m, 1H, leu(1)-C$_\alpha$*H*), 3.57–3.48 (m, 2H, pro(3)-C$_\delta$*H*$_2$), 3.43 (dd, *J* = 13.9, 2.7 Hz, 1H, phe(2)-C*H*H), 3.41–3.28 (m, 4H, pro(1)-C$_\delta$*H*$_2$, pro(2)-C$_\delta$*H*$_2$), 3.07–3.02 (m, 2H, phe(3)-C*H*$_2$), 2.91 (dd, *J* = 13.9, 5.1 Hz, 1H, phe(1)-C*H*H), 2.78 (dd, *J* = 13.9, 10.8 Hz, 1H, phe(1)-CH*H*), 2.66–2.58 (m, 1H, phe(2)-CH*H*), 2.24–2.11 (m, 3H, pro(1)-C$_\beta$*H*H, pro(3)-C$_\beta$*H*$_2$), 2.10–2.00 (m, 2H, pro(1)-C$_\beta$H*H*, pro(2)-C$_\beta$*H*H), 1.86–1.71 (m, 6H, pro(1)-C$_\gamma$*H*H, pro(2)-C$_\beta$H*H*, pro(2)-C$_\gamma$*H*$_2$, pro(3)-C$_\gamma$*H*$_2$), 1.68–1.34 (m, 15H, 2 × leu(1)-C*H*, 2 × leu(2)-C*H*, 2 × leu(3)-C*H*, 2 × leu(1)-C*H*$_2$, leu(2)-C*H*$_2$, leu(3)-C*H*$_2$, pro(1)-C$_\gamma$H*H*), 1.27–1.22 (m, 2H, leu(3)-C*H*$_2$), 1.19 (dt, *J* = 13.3, 6.7 Hz, 1H, leu(2)-C*H*H), 1.08 (dt, *J* = 13.7, 6.9 Hz, 1H, leu(2)-CH*H*), 0.97 (d, *J* = 6.4 Hz, 3H, leu(1)-C*H*$_3$), 0.95–0.80 (m, 21H, 3 ×leu(1)-C*H*$_3$, leu(2)-C*H*$_3$, 3 ×leu(3)-C*H*$_3$), 0.79 (d, *J* = 6.2 Hz, 3H, leu(2)-C*H*$_3$), 0.71 (d, *J* = 6.5 Hz, 3H, leu(3)-C*H*$_3$), 0.67 (d, *J* = 6.6 Hz, 3H, leu(2)-C*H*$_3$), 0.58 (d, *J* = 6.6 Hz, 3H, leu(2)-C*H*$_3$). – **^{13}C-NMR** (125 MHz, DMSO-d_6, ppm): δ = 173.3 (C$_q$, (1)-CON), 172.9 (C$_q$, (3)-CON), 172.8 (C$_q$, (2)-CON), 172.1 (C$_q$, (3)-CON), 172.0 (C$_q$, (1)-CON), 171.9 (C$_q$, (3)-CON), 171.7 (C$_q$, (2)-CON), 171.4 (C$_q$, (1)-CON), 171.3 (C$_q$, (3)-CON), 170.2 (C$_q$, (2)-CON), 169.9 (C$_q$, (2)-CON), 169.6 (C$_q$, (1)-CON), 138.8 (C$_q$, phe(2)-C$_{ar}$), 137.5 (C$_q$, phe(1)-C$_{ar}$), 137.5 (C$_q$, phe(3)-C$_{ar}$), 128.9 (+, 2 × phe(3)-CH), 128.8 (+, 2 × phe(1)-CH, 2 × phe(2)-CH), 124.1 (+, 2 × phe(2)-CH), 128.2 (+, 2 × phe(3)-CH), 128.1 (+, 2 × phe(1)-CH), 126.5 (+, phe(3)-CH), 126.4 (+, phe(1)-CH), 126.3 (+, phe(2)-CH), 58.5 (+, pro(1)-C$_\alpha$H), 58.4 (+, phe(2)-C$_\alpha$H), 58.0 (+, phe(3)-C$_\alpha$H), 57.9 (+, pro(2)-C$_\alpha$H), 57.2 (+, pro(3)-C$_\alpha$H), 56.3 (+, leu(1)-C$_\alpha$H), 56.0 (+, phe(1)-C$_\alpha$H), 55.4 (+, leu(2)-C$_\alpha$H), 53.7 (+, leu(2)-C$_\alpha$H, leu(3)-C$_\alpha$H), 49.4 (+, leu(3)-C$_\alpha$H), 48.0 (–, pro(2)-C$_\delta$H$_2$), 47.6 (–, pro(1)-C$_\delta$H$_2$), 45.7 (–, pro(3)-C$_\delta$H$_2$), 44.8 (+, leu(1)-C$_\alpha$H), 39.4 (–, leu(1)-CH$_2$), 39.6 (–, leu(3)-CH$_2$), 38.8 (–, leu(1)-CH$_2$), 38.1 (–, leu(3)-CH$_2$), 38.4 (–, leu(3)-CH$_2$), 37.4 (–, leu(2)-CH$_2$), 37.2 (–, phe(1)-CH$_2$), 36.3 (–, phe(2)-CH$_2$), 35.9 (–, phe(3)-CH$_2$), 32.2 (–, pro(1)-C$_\beta$H$_2$), 32.1 (–, pro(3)-C$_\beta$H$_2$), 32.0 (–, pro(2)-C$_\beta$H$_2$), 24.5 (+, leu(3)-CH), 24.4 (+, leu(2)-CH), 24.3 (+, leu(1)-CH, leu(2)-CH), 24.1 (+, leu(1)-CH), 23.8 (+, leu(3)-CH), 22.9 (+, leu(1)-CH$_3$, leu(3)-CH$_3$), 22.8 (+, leu(2)-CH$_3$, leu(3)-CH$_3$), 22.5 (+, leu(1)-CH$_3$), 22.3 (+, leu(2)-CH$_3$), 22.1 (+, leu(2)-CH$_3$, leu(3)-CH$_3$), 22.0 (+, leu(1)-CH$_3$), 21.2 (+, leu(2)-CH$_3$, leu(3)-CH$_3$), 20.9 (–, pro(1)-C$_\gamma$H$_2$), 20.8 (+, leu(1)-CH$_3$), 20.1 (–, pro(2)-C$_\gamma$H$_2$, pro(3)-C$_\gamma$H$_2$). – **Analytical HPLC** (5–95% acetonitrile + 0.1% TFA in 30 min, detection at 218 nm): t_{Ret} = 12.3 min (>99%). – **MS** (m/z, MALDI-TOF): 471 [M+H]$^+$, 493 [M+Na]$^+$, 509 [M+K]$^+$.

***cyclo*-(L-Leu-D-Pro-L-Nle-L-Phe) (7e)**

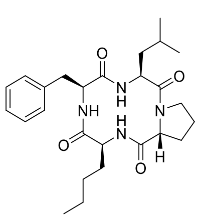

Fmoc-L-phenylalanine (310 mg, 800 µmol, 4.00 equiv.) was immobilized in accordance with **GP1**. Subsequently, Fmoc-L-norleucine (283 mg, 800 µmol, 4.00 equiv.), Fmoc-D-proline (270 mg, 800 µmol, 4.00 equiv.) and Fmoc-L-leucine (283 mg, 800 µmol, 4.00 equiv.) were coupled following **GP3**. Cleavage was performed according to **GP5**. The crude linear precursor (49.0 mg, 100 µmol, 1.00 equiv.) was cyclized following **GP6**. After purification *via* preparative reversed phase HPLC (5–95% acetonitrile in water with 0.1% TFA in 45 min), the macrocycle was isolated as colorless solid (26.3 mg, 56.0 µmol, 56% over 10 steps).

^{1}H-NMR (500 MHz, acetonitrile-d_3 / D_2O, ppm): δ = 7.32–7.11 (m, 8H, leu-N$_\alpha$*H*, nle-N$_\alpha$*H*, 5 × phe-C*H*, phe-N$_\alpha$*H*), 4.56 (d, *J* = 8.2 Hz, 1H, pro-C$_\alpha$*H*), 4.49–4.36 (m, 2H, phe-C$_\alpha$*H*, nle-C$_\alpha$*H*), 3.76–3.67 (m, 1H, leu-C$_\alpha$*H*), 3.49–3.41 (m, 1H, pro-C$_\delta$*H*H), 3.39–3.30 (m, 1H, pro-C$_\delta$H*H*), 2.97 (dd, *J* = 14.5, 4.9 Hz, 1H, phe-C*H*H), 2.81–2.70 (m, 1H, phe-CH*H*), 2.22–2.11 (m, 1H, pro-C$_\beta$*H*H), 2.11–2.01 (m, 1H, pro-C$_\beta$H*H*), 1.85–1.38 (m, 7H, leu-C*H*, leu-C*H*$_2$, nle-C$_\beta$*H*$_2$, pro-C$_\gamma$*H*$_2$), 1.26–1.07 (m, 3H, nle-C$_\gamma$*H*$_2$, nle-C$_\delta$*H*H), 0.99–0.73 (m, 10H, 2 × leu-C*H*$_3$, nle-C$_\delta$H*H*, nle-C*H*$_3$). – **^{13}C-NMR** (125 MHz, acetonitrile-d_3 / D_2O, ppm): δ = 175.8 (C$_q$, CON), 172.9 (C$_q$, 2 × CON), 171.5 (C$_q$, CON), 136.7 (C$_q$, phe-C$_{ar}$), 129.0 (+, 2 × phe-CH), 128.3 (+, 2 × phe-CH), 126.7 (+, phe-CH), 59.1 (+, pro-C$_\alpha$H), 58.3 (+, leu-C$_\alpha$H), 56.7 (+, phe-C$_\alpha$H), 54.4 (+, nle-C$_\alpha$H), 48.2 (–, pro-C$_\delta$H$_2$), 39.1 (–, leu-CH$_2$), 37.2 (–, phe-CH$_2$), 32.1 (–, pro-C$_\beta$H$_2$), 29.0 (–, nle-C$_\beta$H$_2$), 27.1 (–, nle-C$_\delta$H$_2$), 24.4 (+, leu-CH), 22.1 (–, nle-C$_\gamma$H$_2$), 21.7 (+, leu-CH$_3$), 21.6 (+, leu-CH$_3$), 20.2 (–, pro-C$_\gamma$H$_2$), 13.0 (+, nle-CH$_3$). – **Analytical HPLC** (5–95% acetonitrile + 0.1% TFA in 30 min, detection at 218 nm): t_{Ret} = 12.5 min (>99%). – **MS** (m/z, MALDI-TOF): 471 [M+H]$^+$, 493 [M+Na]$^+$, 509 [M+K]$^+$.

***cyclo*-(L-Leu-D-Pro-L-Nle-L-Trp) (7f)**

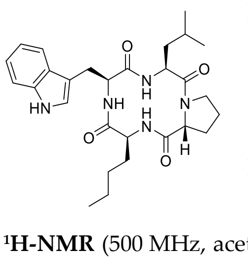

Fmoc-L-tryptophan (341 mg, 800 µmol, 4.00 equiv.) was immobilized in accordance with **GP1**. Subsequently, Fmoc-L-norleucine (283 mg, 800 µmol, 4.00 equiv.), Fmoc-D-proline (270 mg, 800 µmol, 4.00 equiv.) and Fmoc-L-leucine (283 mg, 800 µmol, 4.00 equiv.) were coupled following **GP3**. Cleavage was performed according to **GP5**. The crude linear precursor (52.8 mg, 100 µmol, 1.00 equiv.) was cyclized following **GP6**. After purification *via* preparative reversed-phase HPLC (5–95% acetonitrile in water with 0.1% TFA in 45 min), the macrocycle was isolated as colorless solid (36.7 mg, 72.0 µmol, 36% over 10 steps).

^{1}H-NMR (500 MHz, acetonitrile-d_3 / D_2O, ppm): δ = 7.58–7.47 (m, 2H, leu-N$_\alpha$*H*, trp-C*H*), 7.38–7.31 (m, 2H, nle-N$_\alpha$*H*, trp-C*H*), 7.16–6.96 (m, 5H, 4 × trp-C*H*, trp-N$_\alpha$*H*, trp-N*H*), 4.64–4.51 (m, 2H, pro-C$_\alpha$*H*, trp-C$_\alpha$*H*), 4.45–4.32 (m, 1H, nle-C$_\alpha$*H*), 3.81–3.67 (m, 1H, leu-C$_\alpha$*H*), 3.58–3.26 (m, 2H, pro-C$_\delta$*H*$_2$), 3.15–3.05 (m, 1H, trp-C*H*H), 3.03–2.91 (m, 1H, trp-CH*H*), 2.23–2.09 (m, 1H, pro-C$_\beta$*H*H), 2.09–2.00 (m, 1H, pro-C$_\beta$H*H*), 1.84–1.74 (m, 1H, pro-C$_\gamma$*H*H), 1.66–1.32 (m, 6H, leu-C*H*, leu-C*H*$_2$, nle-C$_\beta$*H*$_2$, pro-C$_\gamma$H*H*), 1.25–1.12 (m, 3H, nle-C$_\gamma$*H*$_2$, nle-C$_\delta$*H*H), 1.04–0.93 (m, 1H, nle-C$_\delta$H*H*), 0.93–0.65 (m, 9H, 2 × leu-C*H*$_3$, nle-C*H*$_3$). – **^{13}C-NMR** (125 MHz, acetonitrile-d_3 / D_2O, ppm): δ = 175.9 (C$_q$, *C*ON), 173.3 (C$_q$, *C*ON), 172.9 (C$_q$, *C*ON), 171.5 (C$_q$, *C*ON), 136.0 (C$_q$, trp-C$_{ar}$), 127.2 (C$_q$, trp-C$_{ar}$), 123.5 (+, trp-CH), 124.3 (+, trp-CH), 121.4 (+, trp-CH), 118.8 (+, trp-CH), 111.3 (+, trp-CH), 109.5 (C$_q$, trp-C$_{ar}$), 59.0 (+, pro-C$_\alpha$H), 58.3 (+, leu-C$_\alpha$H), 56.7 (+, trp-C$_\alpha$H), 54.4 (+, nle-C$_\alpha$H), 48.2 (–, pro-C$_\delta$H$_2$), 38.9 (–, leu-CH$_2$), 32.1 (–, pro-C$_\beta$H$_2$), 28.9 (–, nle-C$_\beta$H$_2$), 27.4 (–, trp-CH$_2$), 27.2 (–, nle-C$_\delta$H$_2$), 24.3 (+, leu-CH), 22.0 (–, nle-C$_\gamma$H$_2$), 21.7 (+, leu-CH$_3$), 20.2 (+, leu-CH$_3$), 20.2 (–, pro-C$_\gamma$H$_2$), 13.0 (+, nle-CH$_3$). – **Analytical HPLC** (5–95% acetonitrile + 0.1% TFA in 30 min, detection at 218 nm): t_{Ret} = 12.3 min (>99%). – **MS** (m/z, MALDI-TOF): 510 [M+H]$^+$, 532 [M+Na]$^+$, 548 [M+K]$^+$.

***cyclo*-(L-Leu-D-Pro-L-Ser-L-Trp) (7g)**

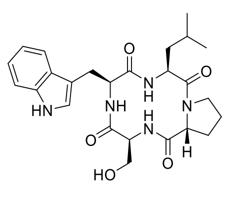

Fmoc-L-tryptophan (341 mg, 800 µmol, 4.00 equiv.) was immobilized in accordance with **GP1**. Subsequently, *O*-^{tert}Butyl-Fmoc-L-serine (307 mg, 800 µmol, 4.00 equiv.), Fmoc-D-proline (270 mg, 800 µmol, 4.00 equiv.) and Fmoc-L-leucine (283 mg, 800 µmol, 4.00 equiv.) were coupled following **GP3**. Cleavage was performed according to **GP5**. The crude linear precursor (55.8 mg, 100 µmol, 1.00 equiv.) was cyclized following **GP6** and deprotected in accordance with **GP7**. After purification *via* preparative reversed phase HPLC (5–95% acetonitrile in water with 0.1% TFA in 45 min), the macrocycle was isolated as colorless solid (21.6 mg, 44.7 µmol, 22% over 11 steps).

The NMR spectra contain multiple signal sets. Chemical shifts of the most prominent isomer are given. **^{1}H-NMR** (500 MHz, DMSO-d_6, ppm): δ = 10.9 (bs, 1H, trp-N*H*), 8.14 (d, *J* = 9.9 Hz, 1H, ser-N$_\alpha$*H*), 7.33 (d, *J* = 7.8 Hz, 1H, trp-N$_\alpha$*H*), 6.99–6.95 (m, 2H, 2 × trp-C*H*), 6.93 (tt, *J* = 7.6, 1.0 Hz, 1H, trp-C*H*), 6.67 (d, *J* = 4.2 Hz, 1H, leu-N$_\alpha$*H*), 6.56 (td, *J* = 7.4, 1.0 Hz, 1H, trp-C*H*), 6.49 (d, *J* = 7.7 Hz, 1H, trp-C*H*), 4.68 (dt, *J* = 9.8, 6.2 Hz, 1H, ser-C$_\alpha$*H*), 4.44 (d, *J* = 8.6 Hz, 1H, trp-C$_\alpha$*H*), 4.26 (d, *J* = 8.3 Hz, 1H, pro-C$_\alpha$*H*), 3.69 (dd, *J* = 11.1, 6.0 Hz, 1H, ser-C*H*H), 3.44 (dd, *J* = 11.1, 6.5 Hz, 1H, ser-CH*H*), 3.34–3.19 (m, 3H, leu-C$_\alpha$*H*, pro-C$_\delta$*H*$_2$), 2.65–2.57 (m, 1H,trp-C*H*H), 2.46–2.39 (m, 1H,trp-CH*H*), 2.04–1.93 (m, 1H, pro-C$_\beta$*HH*), 1.91–1.84 (m, 1H, pro-C$_\beta$*HH*), 1.84–1.75 (m, 1H, pro-C$_\gamma$*H*H), 1.70–1.62 (m, 1H, pro-C$_\gamma$H*H*), 1.42–1.25 (m, 2H, leu-C*H*, leu-C*H*H), 1.13–1.07 (m, 1H, leu-CH*H*), 0.78 (d, *J* = 6.4 Hz, 3H, leu-C*H*$_3$), 0.69 (d, *J* = 6.4 Hz, 3H, leu-C*H*$_3$). – **^{13}C-NMR** (125 MHz, DMSO-d_6, ppm): δ = 171.8 (C$_q$, *C*O), 170.8 (C$_q$, *C*O), 170.1 (C$_q$, *C*O), 169.2 (C$_q$, *C*O), 136.5 (C$_q$, trp-C$_{ar}$), 129.7 (C$_q$, trp-C$_{ar}$), 128.8 (+, trp-C$_{ar}$H), 128.0 (+, trp-C$_{ar}$H), 124.4 (+, trp-C$_{ar}$H), 118.2 (+, trp-C$_{ar}$H), 111.4 (C$_q$, trp-C$_{ar}$), 108.9 (+, trp-C$_{ar}$H), 61.2 (+, trp-C$_\alpha$H), 60.8 (–, ser-CH$_2$), 57.7 (+, pro-C$_\alpha$H), 55.6 (+ leu-C$_\alpha$H), 50.2 (+, ser-C$_\alpha$H), 47.8 (–, pro-C$_\delta$H$_2$), 37.6 (–, leu-CH$_2$), 34.8 (–, trp-CH$_2$), 31.8 (–, pro-C$_\beta$H$_2$), 23.9 (+, leu-CH), 22.9 (+, leu-CH$_3$), 21.9 (+, leu-CH$_3$), 20.9 (–, pro-C$_\gamma$H$_2$). – **Analytical HPLC** (5–95% acetonitrile + 0.1% TFA in 30 min, detection at 218 nm): t_{Ret} = 12.6 min (98%). – **MS** (m/z, MALDI-TOF): 484 [M+H]$^+$, 506 [M+Na]$^+$, 522 [M+K]$^+$.

***cyclo*-(L-Phe-D-Pro-L-Nle-L-Trp) (7h)**

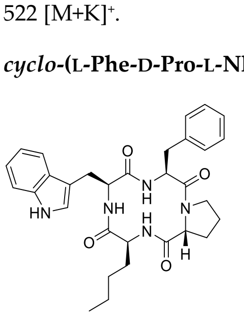

Fmoc-L-tryptophan (341 mg, 800 µmol, 4.00 equiv.) was immobilized in accordance with **GP1**. Subsequently, Fmoc-L-norleucine (283 mg, 800 µmol, 4.00 equiv.), Fmoc-D-proline (270 mg, 800 µmol, 4.00 equiv.) and Fmoc-L-phenylalanine (310 mg, 800 µmol, 4.00 equiv.) were coupled following **GP3**. Cleavage was performed according to **GP5**. The crude linear precursor (56.1 mg, 100 µmol, 1.00 equiv.) was cyclized following **GP6**. After purification *via* preparative reversed phase HPLC (5–95% acetonitrile in water with 0.1% TFA in 45 min), the macrocycle was isolated as colorless solid (25.6 mg, 47.1 µmol, 38% over 10 steps).

The NMR spectra contain multiple signal sets. Chemical shifts of the most prominent isomers are given. Isomer "(1)" is represented in about twice the amount of isomer "(2)". **^{1}H-NMR** (500 MHz, DMSO-d_6, ppm): δ = 10.84–10.76 (m, 2H, trp(1)-N*H*, trp(2)-N*H*), 8.22 (d, *J* = 5.9 Hz, 1H, nle-N$_\alpha$*H*), 8.14 (d, *J* = 9.8 Hz, 1H, nle(2)-N$_\alpha$*H*), 7.62–7.49 (m, 3H, phe(1)-N$_\alpha$*H*, trp(1)-N$_\alpha$*H*, trp(2)-C*H*), 7.44–7.37 (m, 2H, trp(1)-C*H*, phe(2)-N$_\alpha$*H*), 7.36–7.15 (m, 13H, 5 × phe(1)-C*H*, 5 × phe(2)-C*H*, trp(1)-C*H*, trp(2)-C*H*, trp(2)-N$_\alpha$*H*), 7.08–6.88 (m, 5H, 2 × trp(1)-C*H*, 3 × trp(2)-C*H*), 6.81 (d, *J* = 2.1 Hz, 1H, trp(1)-C*H*), 4.85 (d, *J* = 7.8 Hz, 1H, pro(1)-C$_\alpha$*H*), 4.69–4.61 (m, 1H, phe(1)-C$_\alpha$*H*), 4.54 (d, *J* = 8.4 Hz, 1H, pro(2)-C$_\alpha$*H*), 4.51–4.46 (m, 1H, phe(2)-C$_\alpha$*H*), 4.38 (t, *J* = 10.0 Hz, 1H, trp(2)-C$_\alpha$*H*), 4.32–4.25 (m, 1H, trp(1)-C$_\alpha$*H*), 4.22–4.08 (m, 1H, nle(2)-C$_\alpha$*H*), 3.71 (q, *J* = 7.4 Hz, 1H, nle(1)-

C$_{\alpha}$*H*), 3.49–3.26 (m, 5H, pro(1)-C$_{\delta}$*H*$_2$, pro(2)-C$_{\delta}$*H*$_2$, trp(2)-C*H*H), 3.25–3.18 (m, 1H, phe(2)-C*H*H), 3.05–2.96 (m, 2H, phe(1)-C*H*H, phe(2)-CH*H*), 2.93–2.85 (m, 1H, phe(1)-CH*H*), 2.80 (dd, *J* = 14.5, 10.7 Hz, 1H, trp(2)-CH*H*), 2.75–2.66 (m, 1H, trp(1)-C*H*H), 2.65–2.59 (m, 1H, trp(1)-C*H*H), 2.29–2.18 (m, 1H, pro(1)-C$_{\beta}$*H*H), 2.17–2.09 (m, 2H, pro(1)-C$_{\beta}$H*H*, pro(2)-C$_{\beta}$*H*H), 1.91–1.74 (m, 4H, pro(2)-C$_{\beta}$H*H*, pro(1)-C$_{\gamma}$*H*H, pro(2)-C$_{\gamma}$*H*$_2$), 1.66–1.54 (m, 2H, nle(1)-C$_{\beta}$*H*H, pro(1)-C$_{\gamma}$H*H*), 1.53–1.44 (m, 1H, nle(1)-C$_{\beta}$H*H)*, 1.42–1.33 (m, 1H, nle(2)-C$_{\beta}$*H*H), 1.28–1.16 (m, 3H, nle(1)-C$_{\gamma}$*H*$_2$, nle(1)-C$_{\delta}$*H*H), 1.12–1.03 (m, 2H, nle(2)-C$_{\beta}$H*H*, nle(1)-C$_{\delta}$H*H*), 0.98–0.84 (m, 2H, nle(2)-C$_{\gamma}$*H*$_2$), 0.79 (t, *J* = 7.0 Hz, 3H, nle(1)-C*H*$_3$), 0.73–0.67 (m, 1H, nle(2)-C$_{\delta}$*H*H), 0.64 (t, *J* = 7.3 Hz, 3H, nle(2)-C*H*$_3$), 0.60–0.54 (m, 1H, nle(2)-C$_{\delta}$H*H*). – ^{13}**C-NMR** (125 MHz, DMSO-*d*$_6$, ppm): δ = 173.7 (C$_q$, (1)-CON), 173.2 (C$_q$, (2)-CON), 172.5 (C$_q$, (2)-CON), 172.3 (C$_q$, (1)-CON, (2)-CON), 171.9 (C$_q$, (1)-CON), 169.3 (C$_q$, (2)-CON), 168.9 (C$_q$, (1)-CON), 137.6 (C$_q$, phe(2)-C$_{ar}$), 137.2 (C$_q$, phe(1)-C$_{ar}$), 136.4 (C$_q$, trp(2)-C$_{ar}$), 136.0 (C$_q$, trp(1)-C$_{ar}$), 129.1 (C$_q$, trp(1)-C$_{ar}$), 128.9 (+, 2 × phe(1)-CH), 128.5 (+, 2 × phe(2)-CH), 128.4 (+, 2 × phe(2)-CH), 128.3 (C$_q$, trp(2)-C$_{ar}$), 128.2 (+, 2 × phe(1)-CH), 127.2 (+,phe(2)-CH), 126.6 (+, phe(1)-CH), 124.0 (+, trp(2)-CH), 122.8 (+, trp(1)-CH), 120.9 (+, trp(1)-CH, trp(2)-CH), 118.4 (+, trp(2)-CH), 118.3 (+, trp(2)-CH), 118.2 (+, trp(1)-CH), 118.0 (+, trp(1)-CH), 111.5 (+, trp(2)-CH), 111.4 (+, trp(1)-CH), 110.2 (C$_q$, trp(1)-C$_{ar}$, trp(2)-C$_{ar}$), 58.6 (+, pro(1)-C$_{\alpha}$H), 58.3 (+, phe(2)-C$_{\alpha}$H), 58.0 (+, pro(2)-C$_{\alpha}$H), 57.9 (+, nle(1)-C$_{\alpha}$H), 56.8 (+, phe(1)-C$_{\alpha}$H), 56.7 (+, nle(2)-C$_{\alpha}$H), 56.2 (+, trp(1)-C$_{\alpha}$H), 48.1 (–, pro(2)-C$_{\delta}$H$_2$), 47.7 (–, pro(1)-C$_{\delta}$H$_2$), 46.4 (+, trp(2)-C$_{\alpha}$H), 37.2 (–, phe(1)-CH$_2$), 34.2 (–, phe(2)-CH$_2$), 32.3 (–, pro(1)-C$_{\beta}$H$_2$), 32.2 (–, pro(2)-C$_{\beta}$H$_2$), 29.9 (–, nle(2)-C$_{\beta}$H$_2$), 29.3 (–, nle(1)-C$_{\beta}$H$_2$), 27.5 (–, nle(1)-C$_{\delta}$H$_2$), 27.3 (–, trp(1)-CH$_2$), 26.9 (–, nle(2)-C$_{\delta}$H$_2$), 26.6 (–, trp(2)-CH$_2$), 21.8 (–, nle(1)-C$_{\gamma}$H$_2$), 21.5 (–, nle(2)-C$_{\gamma}$H$_2$), 21.0 (–, pro(2)-C$_{\gamma}$H$_2$), 20.2 (–, pro(1)-C$_{\gamma}$H$_2$), 13.8 (+, nle(2)-CH$_3$), 13.6 (+, nle(1)-CH$_3$). – **Analytical HPLC** (5–95% acetonitrile + 0.1% TFA in 30 min, detection at 218 nm): t$_{Ret}$ = 13.5 min (>99%). – **MS** (m/z, MALDI-TOF): 544 [M+H]$^+$, 566 [M+Na]$^+$, 582 [M+K]$^+$.

***cyclo*-(L-Trp-D-Pro-L-Leu-L-Trp) (7i)**

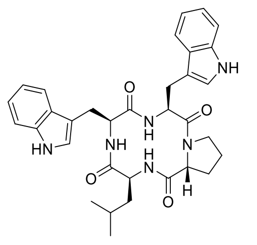

Fmoc-L-tryptophan (341 mg, 800 µmol, 4.00 equiv.) was immobilized in accordance with **GP1**. Subsequently, Fmoc-L-leucine (283 mg, 800 µmol, 4.00 equiv.), Fmoc-D-proline (270 mg, 800 µmol, 4.00 equiv.) and Fmoc-L-tryptophan (341 mg, 800 µmol, 4.00 equiv.) were coupled following **GP3**. Cleavage was performed according to **GP5**. The crude linear precursor (60.1 mg, 100 µmol, 1.00 equiv.) was cyclized following **GP6**. After purification *via* preparative reversed phase HPLC (5–95% acetonitrile in water with 0.1% TFA in 45 min), the macrocycle was isolated as colorless solid (32.2 mg, 55.3 µmol, 54% over 10 steps).

The NMR spectra contain multiple signal sets. Chemical shifts of the most prominent isomers are given. Isomers "(1)" and "(2)" are represented in a similar amount. Isomer "(3)" represents a minor conformation. ^{1}**H-NMR** (500 MHz, DMSO-*d*$_6$, ppm): δ = 10.98–10.75 (m, 6H, 2 × trp(1)-N*H*, 2 × trp(2)-N*H*, 2 × trp(3)-N*H*), 8.55 (dd, *J* = 6.7, 1.7 Hz, 1H, leu(3)-N$_{\alpha}$*H*), 8.18 (d, *J* = 9.9 Hz, 1H, leu(2)-N$_{\alpha}$*H*), 7.90 (d, *J* = 10.3 Hz, 1H, trp(3)-N$_{\alpha}$*H*), 7.86 (d, *J* = 5.7 Hz, 1H, leu(1)-N$_{\alpha}$*H*), 7.80 (d, *J* = 9.0 Hz, 1H, trp(3)-N$_{\alpha}$*H*), 7.59 (d, *J* = 7.9 Hz, 1H, trp(3)-C*H*), 7.56–7.29 (m, 15H, 2 × trp(1)-N$_{\alpha}$*H*, 2 × trp(2)-N$_{\alpha}$*H*, 4 × trp(1)-C*H*, 4 × trp(2)-C*H*, 3 × trp(3)-C*H*), 7.20 (dd, *J* = 5.8, 2.4 Hz, 1H, trp(3)-C*H*), 7.14–6.92 (m, 17H, 6 × trp(1)-C*H*, 6 × trp(2)-C*H*, 5 × trp(3)-C*H*), 5.00 (q, *J* = 8.5 Hz, 1H, trp(3)-C$_{\alpha}$*H*), 4.76–4.72 (m, 2H, pro(2)-C$_{\alpha}$*H*, trp(1)-C$_{\alpha}$*H*), 4.61–4.54 (m, 2H, pro(1)-C$_{\alpha}$*H*, trp(2)-C$_{\alpha}$*H*), 4.48–4.40 (m, 1H, trp(1)-C$_{\alpha}$*H*), 4.36 (td, *J* = 10.5, 4.6 Hz, 1H, trp(2)-C$_{\alpha}$*H*), 4.31–4.25 (m, 1H, pro(3)-C$_{\alpha}$*H*), 4.24–4.16 (m, 2H, leu(2)-C$_{\alpha}$*H*, trp(3)-C$_{\alpha}$*H*), 4.09 (dd, *J* = 8.4, 3.0 Hz, 1H, leu(3)-C$_{\alpha}$*H*), 3.80 (q, *J* = 7.3 Hz, 1H, leu(1)-C$_{\alpha}$*H*), 3.55–3.44 (m, 2H, trp(2)-C*H*H, pro(3)-C$_{\delta}$*H*H), 3.43–3.35 (m, 4H, pro(1)-C$_{\delta}$*H*$_2$, pro(2)-C$_{\delta}$*H*$_2$), 3.34–3.27 (m, 1H, trp(2)-C*H*H), 3.19–3.12 (m, 3H, pro(3)-C$_{\delta}$H*H*, trp(2)-CH*H*, trp(3)-C*H*H), 3.10 (d, *J* = 7.0 Hz, 2H, trp(1)-C*H*$_2$), 3.06–2.99 (m, 1H, trp(3)-CH*H*), 2.86–2.71 (m, 5H, trp(1)-C*H*$_2$, trp(2)-CH*H*, trp(3)-C*H*$_2$), 2.23–2.09 (m, 5H, pro(1)-C$_{\beta}$*H*H, pro(2)-C$_{\beta}$*H*$_2$, pro(3)-C$_{\beta}$*H*$_2$), 1.94–1.85 (m, 1H, pro(2)-C$_{\gamma}$*H*H), 1.85–1.76 (m, 3H, pro(1)-C$_{\beta}$H*H*, pro(1)-C$_{\gamma}$*H*H, pro(2)-C$_{\gamma}$H*H*), 1.74–1.67 (m, 2H, pro(3)-C$_{\gamma}$*H*$_2$), 1.65–1.56 (m, 1H, pro(1)-C$_{\gamma}$H*H*), 1.52–1.44 (m, 3H, leu(1)-C*H*, leu(1)-C*H*H, leu(3)-C*H*), 1.43–1.36 (m, 3H, leu(1)-CH*H*, leu(3)-C*H*$_2$), 1.26–1.17 (m, 1H, leu(2)-C*H*H), 1.13–1.01 (m, 2H, leu(2)-C*H*, leu(2)-CH*H*), 0.86 (d, *J* = 5.7 Hz, 3H, leu(1)-C*H*$_3$), 0.83–0.80 (m, 6H, 2 × leu(3)-C*H*$_3$), 0.78 (dd, *J* = 5.6 Hz, 3H, leu(1)-C*H*$_3$), 0.52 (d, *J* = 6.2 Hz, 3H, leu(2)-C*H*$_3$), 0.39 (d, *J* = 6.3 Hz, 3H, leu(2)-C*H*$_3$). – ^{13}**C-NMR** (125 MHz, DMSO-*d*$_6$, ppm): δ = 173.7 (C$_q$, (1)-CON), 173.3 (C$_q$, (2)-CON), 173.1 (C$_q$, (3)-CON), 172.3 (C$_q$, (1)-CON), 172.0 (C$_q$, (2)-CON), 171.8 (C$_q$, (3)-CON), 171.6 (C$_q$, (2)-CON), 171.1 (C$_q$, (3)-CON), 170.8 (C$_q$, (3)-CON), 170.6 (C$_q$, (2)-CON), 169.9 (C$_q$, (1)-CON), 169.3 (C$_q$, (1)-CON), 136.4 (C$_q$, trp(2)-C$_{ar}$), 136.2 (C$_q$, trp(1)-C$_{ar}$, trp(2)-C$_{ar}$), 136.1 (C$_q$, trp(3)-C$_{ar}$), 136.0 (C$_q$, trp(1)-C$_{ar}$, trp(3)-C$_{ar}$), 127.2 (C$_q$, trp(2)-C$_{ar}$), 127.1 (C$_q$, 2 × trp(3)-C$_{ar}$), 127.0 (C$_q$, 2 × trp(1)-C$_{ar}$), 126.5 (C$_q$, trp(2)-C$_{ar}$), 124.0 (+, trp(3)-CH), 123.9 (+, trp(1)-CH), 123.6 (+, trp(3)-CH), 123.3 (+, trp(2)-CH), 122.9 (+, trp(2)-CH), 122.8 (+, trp(1)-CH), 121.2 (+, trp(1)-CH, trp(2)-CH), 121.0 (2 × trp(3)-CH), 120.9 (+, trp(1)-CH, trp(2)-CH), 118.5–117.9 (+, 4 × trp(1)-CH, 4 × trp(2)-CH, 4 × trp(3)-CH), 111.5–111.4 (+, 2 × trp(1)-CH, 2 × trp(2)-CH, 2 × trp(3)-CH), 110.4 (C$_q$, trp(2)-C$_{ar}$,), 110.3 (C$_q$, trp(1)-C$_{ar}$), 110.1 (C$_q$, trp(2)-C$_{ar}$), 109.9 (C$_q$, trp(3)-C$_{ar}$), 109.6 (C$_q$, trp(1)-C$_{ar}$), 109.1 (C$_q$, trp(3)-C$_{ar}$), 60.0 (+,-leu(3)-C$_{\alpha}$H), 58.7 (+, trp(1)-C$_{\alpha}$H), 58.1 (+, trp(2)-C$_{\alpha}$H), 58.0 (+, trp(3)-C$_{\alpha}$H), 57.9 (+, pro(1)-C$_{\alpha}$H), 56.8 (+, trp(1)-C$_{\alpha}$H), 56.2 (+, trp(2)-C$_{\alpha}$H), 56.1 (+, leu(1)-C$_{\alpha}$H), 55.8 (+, pro(2)-C$_{\alpha}$H), 53.8 (+, pro(3)-C$_{\alpha}$H), 51.9 (+, trp(3)-C$_{\alpha}$H), 48.1 (–, pro(2)-C$_{\delta}$H$_2$), 47.7 (–, pro(1)-C$_{\delta}$H$_2$), 45.7 (–, pro(3)-C$_{\delta}$H$_2$), 44.9 (+, leu(2)-C$_{\alpha}$H), 38.8 (–, leu(3)-CH$_2$), 38.6 (–, leu(1)-CH$_2$), 38.2 (–, leu(2)-CH$_2$), 32.4 (–, pro(1)-C$_{\beta}$H$_2$), 32.3 (–, pro(3)-C$_{\beta}$H$_2$), 32.2 (–, pro(2)-C$_{\beta}$H$_2$), 27.6 (–, trp(1)-CH$_2$), 27.4 (–, trp(1)-CH$_2$), 26.6 (–, trp(2)-CH$_2$), 25.9 (–, trp(3)-CH$_2$), 25.4 (–, trp(3)-CH$_2$), 25.0 (–, trp(2)-CH$_2$), 24.4 (+, leu(3)-CH), 24.3 (+, leu(1)-CH), 24.0 (+, leu(2)-CH), 22.4 (+, leu(3)-CH$_3$), 22.1 (+, leu(1)-CH$_3$), 22.1 (+, leu(1)-CH$_3$, leu(2)-CH$_3$, leu(3)-CH$_3$), 22.0 (+, leu(2)-CH$_3$), 21.2 (–, pro(3)-C$_{\gamma}$H$_2$), 21.0 (–, pro(2)-C$_{\gamma}$H$_2$), 20.2 (–, pro(1)-C$_{\gamma}$H$_2$). – **Analytical HPLC** (5–95% acetonitrile + 0.1% TFA in 30 min, detection at 218 nm): t$_{Ret}$ = 12.6 min (>99%). – **MS** (m/z, MALDI-TOF): 583 [M+H]$^+$, 605 [M+Na]$^+$, 621 [M+K]$^+$.

***cyclo*-(*N*1ip-D-Pro-L-Nle-L-Phe) (8a)**

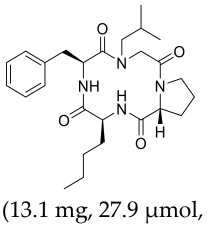

Fmoc-L-phenylalanine (310 mg, 800 μmol, 4.00 equiv.) was immobilized in accordance with **GP1**. Subsequently, Fmoc-L-norleucine (283 mg, 800 μmol, 4.00 equiv.) and Fmoc-D-proline (270 mg, 800 μmol, 4.00 equiv.) were coupled following **GP3**. The peptoid monomer *N*1ip was incorporated following **GP4** using isobutylamine (159 μL, 117 mg, 1.60 mmol, 8.00 equiv.) as submonomer. Cleavage was performed according to **GP5**. The crude linear precursor (48.8 mg, 100 μmol, 1.00 equiv.) was cyclized following **GP6**. After purification *via* preparative reversed phase HPLC (5–95% acetonitrile in water with 0.1% TFA in 45 min), the macrocycle was isolated as colorless solid (13.1 mg, 27.9 μmol, 24% over 10 steps).

The NMR spectra contain multiple signal sets. Chemical shifts of the most prominent isomer are given. **^{1}H-NMR** (500 MHz, acetonitrile-d_3, ppm): δ = 7.37–7.07 (m, 5H, 5 × phe-C*H*), 6.87 (d, *J* = 10.5 Hz, 1H, phe-N$_\alpha$*H*), 5.70 (d, *J* = 7.6 Hz, 1H, nle-N$_\alpha$*H*), 5.05 (td, *J* = 10.6, 4.7 Hz, 1H, phe-C$_\alpha$*H*), 4.21 (dd, *J* = 10.8, 8.0 Hz, 1H, pro-C$_\alpha$*H*), 3.99 (d, *J* = 17.9 Hz, 1H, pro-C$_\delta$*H*H), 3.96–3.89 (m, 1H, COC*H*HN), 3.74 (d, *J* = 17.9 Hz, 1H, pro-C$_\delta$H*H*), 3.55–3.43 (m, 1H, nle-C$_\alpha$*H*), 3.37 (dd, *J* = 10.2, 7.8 Hz, 1H, *N*1ip-C$_1$*H*H), 3.28–3.13 (m, 2H, phe-C*H*H, *N*1ip-C$_1$H*H*), 2.88–2.71 (m, 1H, phe-CH*H*), 2.49 (dd, *J* = 13.4, 6.7 Hz, 1H, COCH*H*N), 2.27–2.17 (m, 1H, pro-C$_\beta$*H*H), 2.08–1.99 (m, 1H, pro-C$_\gamma$*H*H), 1.91–1.70 (m, 3H, pro-C$_\beta$H*H*, pro-C$_\gamma$H*H*, *N*1ip-C$_2$*H*), 1.35–1.05 (m, 6H, 3 × nle-C*H*$_2$), 0.92–0.89 (m, 3H, nle-C*H*$_3$), 0.84–0.79 (m, 6H, 2 × *N*1ip-C*H*$_3$). – **^{13}C-NMR** (125 MHz, acetonitrile-d_3, ppm): δ = 174.5 (C$_q$, *C*O), 171.2 (C$_q$, *C*O), 170.7 (C$_q$, *C*O), 170.3 (C$_q$, *C*O), 139.3 (C$_q$, phe-*C*$_{ar}$), 130.8 (+, 2 × phe-*C*H), 128.6 (+, 2 × phe-CH), 126.9 (+, phe-CH), 63.7 (+, pro-C$_\alpha$H), 58.8 (+, nle-C$_\alpha$H), 57.0 (–, COCH$_2$N), 51.7 (+, phe-*C*$_\alpha$H), 50.2 (–, pro-C$_\delta$H$_2$), 46.5 (–, *N*1ip-C$_1$H$_2$), 38.5 (–, phe-CH$_2$), 34.5 (–, nle-*C*$_\beta$H$_2$), 28.9 (–, nle-*C*$_\gamma$H$_2$), 27.6 (+, *N*1ip-C$_2$H), 26.1 (–, pro-*C*$_\beta$H$_2$), 22.9 (–, pro-*C*$_\gamma$H$_2$), 22.7 (–, nle-*C*$_\delta$H$_2$), 20.5 (+, *N*1ip-CH$_3$), 20.2 (+, *N*1ip-CH$_3$), 14.0 (+, nle-CH$_3$). – **Analytical HPLC** (5–95% acetonitrile + 0.1% TFA in 30 min, detection at 218 nm): t_{Ret} = 14.1 min (>99%). – **MS** (m/z, MALDI-TOF): 471 [M+H]$^+$, 493 [M+Na]$^+$, 509 [M+K]$^+$.

***cyclo*-(*N*1ph-D-Pro-L-Nle-L-Phe) (8b)**

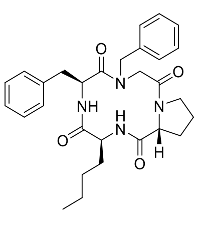

Fmoc-L-phenylalanine (310 mg, 800 μmol, 4.00 equiv.) was immobilized in accordance with **GP1**. Subsequently, Fmoc-L-norleucine (283 mg, 800 μmol, 4.00 equiv.) and Fmoc-D-proline (270 mg, 800 μmol, 4.00 equiv.) were coupled following **GP3**. The peptoid monomer *N*1ph was incorporated following **GP4** using benzylamine (174 μL, 171 mg, 1.60 mmol, 8.00 equiv.) as submonomer. Cleavage was performed according to **GP5**. The crude linear precursor (52.1 mg, 100 μmol, 1.00 equiv.) was cyclized following **GP6**. After purification *via* preparative reversed phase HPLC (5–95% acetonitrile in water with 0.1% TFA in 45 min), the macrocycle was isolated as colorless solid (6.00 mg, 11.9 μmol, 8.4% over 10 steps).

The NMR spectra contain multiple signal sets. Chemical shifts of the most prominent isomer are given. **^{1}H-NMR** (500 MHz, acetonitrile-d_3, ppm): δ = 7.39–7.13 (m, 10H, 5 × phe-C*H*, 5 × *N*1ph-C*H*), 6.89 (dd, *J* = 10.7, 4.2 Hz, 1H, phe-N$_\alpha$*H*), 5.64 (d, *J* = 6.8 Hz, 1H, nle-N$_\alpha$*H*), 5.49 (dd, *J* = 15.4, 4.2 Hz, 1H, COC*H*HN), 5.16 (tt, *J* = 10.1, 4.7 Hz, 1H, phe-C$_\alpha$*H*), 4.28–4.17 (m, 1H, pro-C$_\alpha$*H*), 3.99 (dd, *J* = 18.0, 4.3 Hz, 1H, *N*1ph-C*H*H), 3.82 (dd, *J* = 15.6, 4.4 Hz, 1H, COCH*H*N), 3.65 (dd, *J* = 17.9, 4.4 Hz, 1H, *N*1ph-CH*H*), 3.56–3.46 (m, 1H, nle-C$_\alpha$*H*), 3.32–3.17 (m, 3H, phe-C*H*H, pro-C$_\delta$*H*$_2$), 2.91–2.79 (m, 1H, phe-CH*H*), 2.28–2.19 (m, 1H, pro-C$_\beta$*H*H), 2.07–1.97 (m, 1H, pro-C$_\gamma$*H*H), 1.84–1.72 (m, 2H, pro-C$_\beta$H*H*, pro-C$_\gamma$H*H*), 1.40–1.26 (m, 1H, nle-C$_\gamma$*H*H), 1.23–1.14 (m, 2H, nle-C$_\gamma$H*H*, nle-C$_\delta$*H*H), 1.14–1.02 (m, 2H, nle-C$_\beta$*H*$_2$), 1.00–0.93 (m, 1H, nle-C$_\delta$H*H*), 0.86–0.79 (m, 3H, nle-C*H*$_3$). – **^{13}C-NMR** (125 MHz, acetonitrile-d_3, ppm): δ = 174.5 (C$_q$, *C*ON), 171.5 (C$_q$, *C*ON), 170.8 (C$_q$, *C*ON), 170.0 (C$_q$, *C*ON), 139.2 (C$_q$, *C*$_{ar}$), 138.8 (C$_q$, *C*$_{ar}$), 130.9 (+, 2 × C$_{ar}$H), 129.4 (+, 2 × C$_{ar}$H), 128.7 (+, 2 × C$_{ar}$H), 128.6 (+, C$_{ar}$H), 128.0 (+, 2 × C$_{ar}$H), 127.0 (+, C$_{ar}$H), 63.7 (+, pro-C$_\alpha$H), 58.9 (+, nle-C$_\alpha$H), 52.6 (–, COCH$_2$N), 51.7 (+, phe-C$_\alpha$H), 49.3 (–, *N*1ph-CH$_2$), 46.5 (–, pro-C$_\delta$H$_2$), 38.4 (–, phe-CH$_2$), 34.6 (–, nle-C$_\beta$H$_2$), 28.9 (–, nle-C$_\gamma$H$_2$), 27.7 (–, pro-C$_\beta$H$_2$), 26.1 (–, pro-C$_\gamma$H$_2$), 22.9 (–, nle-C$_\delta$H$_2$), 14.0 (+, nle-CH$_3$). – **Analytical HPLC** (5–95% acetonitrile + 0.1% TFA in 30 min, detection at 218 nm): t_{Ret} = 15.3 min (>99%). – **MS** (m/z, MALDI-TOF): 505 [M+H]$^+$, 527 [M+Na]$^+$, 543 [M+K]$^+$.

***cyclo*-(*N*1ph-D-Pro-L-Nle-L-Trp) (8c)**

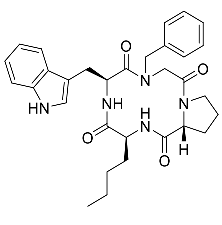

Fmoc-L-tryptophan (341 mg, 800 μmol, 4.00 equiv.) was immobilized in accordance with **GP1**. Subsequently, Fmoc-L-norleucine (283 mg, 800 μmol, 4.00 equiv.) and Fmoc-D-proline (270 mg, 800 μmol, 4.00 equiv.) were coupled following **GP3**. The peptoid monomer *N*1ph was incorporated following **GP4** using benzylamine (174 μL, 171 mg, 1.60 mmol, 8.00 equiv.) as submonomer. Cleavage was performed according to **GP5**. The crude linear precursor (56.0 mg, 100 μmol, 1.00 equiv.) was cyclized following **GP6**. After purification *via* preparative reversed phase HPLC (5–95% acetonitrile in water with 0.1% TFA in 45 min), the macrocycle was isolated as colorless solid (11.7 mg, 21.5 μmol, 21% over 10 steps).

The NMR spectra contain multiple signal sets. Chemical shifts of the most prominent isomers are given. Isomer "(1)" is represented in about four times above the amounts of isomers "(2)" and "(3)". **^{1}H-NMR** (500 MHz, acetonitrile-d_3, ppm): δ = 9.17–9.10 (m, 2H, trp(2)-N*H*, trp(3)-N*H*), 9.06 (s, 1H, trp(1)-N*H*), 7.70 (d, *J* = 7.9 Hz, 1H, trp(2)-C*H*), 7.62 (d, *J* = 7.7 Hz, 2H, trp(1)-C*H*, trp(3)-C*H*), 7.46–7.21 (m, 16H, nle(2)-N$_\alpha$*H*, trp(1)-C*H*, trp(2)-C*H*, trp(3)-C*H*, 4 × *N*1ph(1)-C*H*, 4 × *N*1ph(2)-C*H*, 4 × *N*1ph(3)-C*H*), 7.19–6.96 (m, 11H, nle(3)-N$_\alpha$*H*, 3 × trp(1)-C*H*, 2 × trp(3)-C*H*, 2 × trp(3)-C*H*, *N*1ph(1)-C*H*, *N*1ph(2)-

C*H*, *N*1ph(3)-C*H*), 6.91 (d, *J* = 10.4 Hz, 3H, trp(1)-N$_\alpha$*H*, trp(2)-C*H*, trp(3)-C*H*), 6.61 (d, *J* = 10.7 Hz, 1H, trp(2)-N$_\alpha$*H*), 6.41 (d, *J* = 7.1 Hz, 1H, trp(3)-N$_\alpha$*H*), 5.69 (d, *J* = 7.5 Hz, 1H, nle(1)-N$_\alpha$*H*), 5.52 (d, *J* = 15.4 Hz, 1H, (1)-COC*H*HN), 5.41 (d, *J* = 14.9 Hz, 1H, (2)-COC*H*HN), 5.20 (dt, *J* = 10.2, 5.0 Hz, 1H, trp(1)-C$_\alpha$*H*), 4.97 (d, *J* = 18.0 Hz, 1H, (3)-COC*H*HN), 4.79 (d, *J* = 7.7 Hz, 1H, pro(2)-C$_\alpha$*H*), 4.73–4.61 (m, 3H, trp(3)-C$_\alpha$*H*, trp(2)-C$_\alpha$*H*, *N*1ph(2)-C*H*H), 4.53–4.45 (m, 1H, nle(3)-C$_\alpha$*H*), 4.35 (d, *J* = 18.3 Hz, 1H, (3)-COCH*H*N), 4.24 (dd, *J* = 10.4, 7.9 Hz, 1H, pro(1)-C$_\alpha$*H*), 4.20–4.08 (m, 3H, pro(3)-C$_\alpha$*H*, *N*1ph(2)-CH*H*, *N*1ph(3)-C*H*H), 4.00 (d, *J* = 17.7 Hz, 1H, *N*1ph(1)-C*H*H), 3.90 (t, *J* = 7.5 Hz, 1H, nle(2)-C$_\alpha$*H*), 3.83 (d, *J* = 15.4 Hz, 1H, (1)-COCH*H*N), 3.73 (d, *J* = 13.9 Hz, 1H, *N*1ph(3)-CH*H*), 3.65 (d, *J* = 17.8 Hz, 1H, *N*1ph(1)-CH*H*), 3.51 (q, *J* = 7.2 Hz, 1H, nle(1)-C$_\alpha$*H*), 3.47–3.38 (m, 3H, pro(3)-C$_\delta$*H*$_2$, (2)-COCH*H*N), 3.35 (dd, *J* = 14.8, 5.1 Hz, 1H, trp(1)-C*H*H), 3.29–3.18 (m, 6H, pro(1)-C$_\delta$*H*$_2$, pro(2)-C$_\delta$*H*$_2$, trp(2)-C*H*$_2$), 3.01 (dd, *J* = 14.9, 9.9 Hz, 3H, trp(1)-CH*H*, trp(3)-C*H*$_2$), 2.26–2.19 (m, 3H, pro(1)-C$_\beta$*H*H, pro(3)-C$_\gamma$*H*$_2$), 2.17–2.06 (m, 2H, nle(2)-C$_\beta$*H*H, pro(2)-C$_\gamma$*H*H), 2.04–1.97 (m, 1H, pro(1)-C$_\gamma$*H*H), 1.92–1.87 (m, 2H, pro(2)-C$_\gamma$H*H*, nle(2)-C$_\delta$*H*H), 1.85–1.73 (m, 7H, nle(2)-C$_\beta$H*H*, nle(3)-C$_\delta$*H*H, pro(1)-C$_\beta$H*H*, pro(2)-C$_\beta$*H*$_2$, pro(3)-C$_\beta$*H*$_2$, pro(1)-C$_\gamma$H*H*), 1.71–1.54 (m, 1H, nle(3)-C$_\beta$*H*H), 1.53–1.45 (m, 1H, nle(3)-C$_\delta$H*H*), 1.43–1.16 (m, 4H, nle(3)-C$_\beta$H*H*, nle(1)-C$_\delta$*H*H, nle(2)-C$_\gamma$*H*$_2$), 1.17–1.08 (m, 2H, nle(1)-C$_\gamma$*H*H, nle(3)-C$_\gamma$*H*H, nle(1)-C$_\delta$H*H*, nle(2)-C$_\delta$H*H*), 1.03–0.97 (m, 3H, nle(1)-C$_\beta$*H*$_2$, nle(3)-C$_\gamma$H*H*), 0.93–0.85 (m, 1H, nle(1)-C$_\gamma$H*H*), 0.81 (t, *J* = 7.3 Hz, 6H, nle(2)-C*H*$_3$, nle(3)-C*H*$_3$), 0.79 (t, *J* = 7.3 Hz, 3H, nle(1)-C*H*$_3$). – **^{13}C-NMR** (125 MHz, acetonitrile-*d*$_3$, ppm): δ = 175.2 (C$_q$, (2)-CON), 174.8 (C$_q$, (3)-CON), 174.7 (C$_q$, (1)-CON), 173.4 (C$_q$, (2)-CON), 173.3 (C$_q$, (3)-*C*ON), 172.2 (C$_q$, (2)-*C*ON), 172.1 (C$_q$, (3)-*C*ON), 171.8 (C$_q$, (1)-*C*ON), 170.8 (C$_q$, (1)-*C*ON, (2)-*C*ON, (3)-*C*ON), 170.1 (C$_q$, (1)-*C*ON), 138.8 (C$_q$, *N*1ph(1)-C$_{ar}$, *N*1ph(2)-C$_{ar}$), 138.4 (C$_q$, *N*1ph(3)-C$_{ar}$), 137.3 (C$_q$, trp(2)-C$_{ar}$), 137.2 (C$_q$, trp(1)-C$_{ar}$), 137.1 (C$_q$, trp(3)-C$_{ar}$), 130.0 (C$_q$, trp(2)-*C*$_{ar}$, trp(3)-*C*$_{ar}$), 129.4 (+, 2 × *N*1ph(2)-CH), 129.3 (+, 2 × *N*1ph(1)-CH), 129.2 (+, 2 × *N*1ph(3)-CH), 128.9 (C$_q$, trp(1)-C$_{ar}$), 128.7 (+, 2 × *N*1ph(2)-CH), 128.6 (+, 2 × *N*1ph(1)-CH), 128.5 (+, 2 × *N*1ph(3)-CH), 128.1 (+, *N*1ph(2)-CH), 128.0 (+, *N*1ph(1)-*C*H), 127.5 (+, *N*1ph(3)-CH), 125.0 (+, trp(2)-CH), 124.9 (+, trp(1)-CH), 124.8 (+, trp(3)-CH), 122.7 (+, trp(2)-CH), 122.2 (+, trp(3)-CH), 122.0 (+, trp(1)-CH), 120.2 (+, trp(2)-CH), 119.7 (+, trp(1)-CH, trp(3)-CH), 119.5 (+, trp(1)-CH, trp(2)-CH), 119.2 (+, trp(3)-CH), 112.5 (+, trp(2)-CH), 112.1 (C$_q$, trp(2)-C$_{ar}$), 112.0 (+, trp(1)-CH, trp(3)-CH), 111.8 (C$_q$, trp(1)-*C*$_{ar}$), 110.1 (C$_q$, trp(3)-C$_{ar}$), 63.7 (+, pro(1)-*C*$_\alpha$H), 60.4 (+, pro(2)-*C*$_\alpha$H), 60.1 (+, nle(2)-*C*$_\alpha$H), 58.8 (+, nle(1)-C$_\alpha$H), 54.4 (+, pro(3)-*C*$_\alpha$H), 53.7 (+, trp(2)-*C*$_\alpha$H), 53.1 (+, trp(3)-*C*$_\alpha$H), 52.5 (–, (1)-COCH$_2$N), 51.7 (–, (3)-COCH$_2$N), 51.0 (+, trp(1)-*C*$_\alpha$H), 50.7 (–, *N*1ph(2)-CH$_2$), 50.0 (–, *N*1ph(3)-*C*H$_2$), 49.6 (–, (2)-COCH$_2$N), 49.5 (+, nle(3)-*C*$_\alpha$H), 49.3 (–, *N*1ph(1)-CH$_2$), 48.2 (–, pro(3)-*C*$_\delta$H$_2$), 46.5 (–, pro(1)-*C*$_\delta$H$_2$), 45.5 (–, pro(2)-*C*$_\delta$H$_2$), 34.1 (–, nle(1)-*C*$_\beta$H$_2$), 33.8 (–, nle(2)-*C*$_\beta$H$_2$), 31.1 (–, nle(3)-*C*$_\beta$H$_2$), 30.6 (–, trp(3)-CH$_2$), 30.1 (–, nle(2)-C$_\gamma$H$_2$), 28.9 (–, nle(1)-C$_\gamma$H$_2$), 28.7 (–, nle(3)-C$_\gamma$H$_2$), 28.1 (–, trp(2)-CH$_2$), 28.0 (–, trp(1)-CH$_2$), 27.6 (–, pro(1)-C$_\beta$H$_2$), 26.0 (–, pro(1)-C$_\gamma$H$_2$), 25.3 (–, pro(2)-C$_\beta$H$_2$), 24.6 (–, pro(3)-C$_\beta$H$_2$), 23.2 (–, pro(2)-C$_\gamma$H$_2$), 23.1 (–, pro(3)-C$_\gamma$H$_2$), 23.0 (–, nle(3)-C$_\delta$H$_2$), 22.9 (–, nle(1)-C$_\delta$H$_2$), 22.8 (–, nle(2)-C$_\delta$H$_2$), 14.2 (+, nle(2)-CH$_3$), 14.1 (+, nle(3)-CH$_3$), 14.0 (+, nle(1)-CH$_3$). – **Analytical HPLC** (5–95% acetonitrile + 0.1% TFA in 30 min, detection at 218 nm): t$_{Ret}$ = 10.6 min (>99%). – **MS** (m/z, MALDI-TOF): 544 [M+H]$^+$, 566 [M+Na]$^+$, 582 [M+K]$^+$.

***cyclo*-(L-Ala-D-Pro-*N*3m-L-Phe) (8d)**

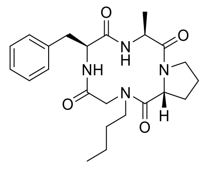

Fmoc-L-phenylalanine (310 mg, 800 µmol, 4.00 equiv.) was immobilized in accordance with **GP1**. The peptoid monomer *N*3m was incorporated following **GP4** using *n*-butylamine (158 µL, 117 mg, 1.60 mmol, 8.00 equiv.) as submonomer. Subsequently, Fmoc-D-proline (270 mg, 800 µmol, 4.00 equiv.) and Fmoc-L-alanine (249 mg, 800 µmol, 4.00 equiv.) were coupled following **GP3**. Cleavage was performed according to **GP5**. The crude linear precursor (44.7 mg, 100 µmol, 1.00 equiv.) was cyclized following **GP6**. After purification *via* preparative reversed phase HPLC (5–95% acetonitrile in water with 0.1% TFA in 45 min), the macrocycle was isolated as colorless solid (15.8 mg, 36.9 µmol, 28% over 10 steps).

The NMR spectra contain multiple signal sets. Chemical shifts of the most prominent isomers are given. Isomer "(1)" is represented in about six times above the amount of isomer "(2)". **^{1}H-NMR** (500 MHz, acetonitrile-*d*$_3$, ppm): δ = 7.56 (d, *J* = 8.9 Hz, 1H, phe-(2)-N$_\alpha$*H*), 7.41–7.16 (m, 13H, ala(1)-N$_\alpha$*H*, ala(2)-N$_\alpha$*H*, 5 × phe(1)-C*H*, 5 × phe(2)-C*H*, phe(1)-N$_\alpha$*H*), 5.07–4.88 (m, 1H, phe(1)-C$_\alpha$*H*), 4.76–4.70 (m, 1H, ala(2)-C$_\alpha$*H*), 4.70–4.62 (m, 2H, phe(2)-C$_\alpha$*H*, pro(2)-C$_\alpha$*H*), 4.51–4.37 (m, 1H, pro(1)-C$_\alpha$*H*), 4.34 (d, *J* = 16.9 Hz, 1H, *N*3m(2)-C$_1$*H*H), 4.00–3.86 (m, 3H, ala(1)-C$_\alpha$*H*, (2)-COC*H*$_2$N), 3.82–3.69 (m, 2H, (1)-COC*H*HN, *N*3m(1)-C$_1$*H*H), 3.59–3.36 (m, 6H, pro(1)-C$_\delta$*H*$_2$, pro(2)-C$_\delta$*H*$_2$, *N*3m(1)-C$_1$H*H*, (1)-COCH*H*N), 3.23 (dd, *J* = 13.5, 7.5 Hz, 3H, phe(1)-C*H*H, phe(2)-C*H*$_2$), 2.79–2.72 (m, 1H, phe(1)-CH*H*), 2.68–2.58 (m, 1H, *N*3m(1)-C$_1$H*H*), 2.36–2.23 (m, 1H, pro(1)-C$_\beta$*H*H), 2.24–2.13 (m, 1H, pro(2)-C$_\beta$*H*H), 2.15–2.04 (m, 1H, pro(1)-C$_\gamma$*H*H), 1.94–1.83 (m, 5H, pro(1)-C$_\beta$H*H*, pro(2)-C$_\beta$H*H*, pro(1)-C$_\gamma$H*H*, pro(1)-C$_\gamma$*H*$_2$), 1.54–1.41 (m, 4H, *N*3m(1)-C$_2$*H*$_2$, *N*3m(2)-C$_2$*H*$_2$), 1.35–1.26 (m, 7H, ala(2)-C*H*$_3$, *N*3m(1)-C$_3$*H*$_2$, *N*3m(2)-C$_3$*H*$_2$), 1.25 (d, *J* = 6.9 Hz, 3H, ala(1)-C*H*$_3$), 0.93 (t, *J* = 7.3 Hz, 3H, *N*3m(1)-C*H*$_3$), 0.87 (t, *J* = 7.5, 1.7 Hz, 3H, *N*3m(2)-C*H*$_3$). – **^{13}C-NMR** (125 MHz, acetonitrile-*d*$_3$, ppm): δ = 174.0 (C$_q$, (1)-CON, (2)-CON), 172.9 (C$_q$, (1)-CON), 171.9 (C$_q$, (1)-CON, (2)-CON), 171.4 (C$_q$, (2)-CON), 167.7 (C$_q$, (1)-CON, (2)-CON), 139.3 (C$_q$, phe(1)-C$_{ar}$), 138.2 (C$_q$, phe(2)-C$_{ar}$), 130.4 (+, 2 × phe(1)-CH), 130.0 (+, phe(2)-CH), 129.9 (+, phe(2)-CH), 128.9 (+, phe(2)-CH), 128.7 (+, phe(2)-CH), 128.5 (+, 2 × phe(1)-CH), 130.0 (+, phe(2)-CH), 127.2 (+, phe(2)-CH), 126.7 (+, phe(1)-CH), 64.4 (+, pro(1)-*C*$_\alpha$H), 57.5 (+, pro(2)-*C*$_\alpha$H), 57.2 (+, phe(2)-C$_\alpha$H), 51.5 (+, phe(1)-C$_\alpha$H), 51.1 (–, (1)-COCH$_2$N), 51.0 (–, *N*3m(2)-*C*$_1$H$_2$), 50.2 (+, ala(1)-*C*$_\alpha$H), 49.9 (–, *N*3m(1)-*C*$_1$H$_2$), 48.2 (–, (2)-COCH$_2$N), 46.8 (–, pro(1)-*C*$_\delta$H$_2$), 46.2 (–, pro(2)-*C*$_\delta$H$_2$), 37.8 (–, phe(1)-CH$_2$), 36.9 (–, phe(2)-CH$_2$), 31.3 (–, pro(2)-C$_\beta$H$_2$), 29.5 (–, *N*3m(2)-C$_2$H$_2$), 28.9 (–, *N*3m(1)-C$_2$H$_2$), 26.9 (–, pro(1)-C$_\beta$H$_2$), 25.7 (–, pro(1)-C$_\gamma$H$_2$), 22.1 (–, pro(2)-C$_\gamma$H$_2$), 20.5 (–, *N*3m(1)-C$_3$H$_2$), 20.2 (–, *N*3m(2)-C$_3$H$_2$), 20.0 (+, ala(2)-CH$_3$), 17.4 (+, ala(1)-CH$_3$), 13.8 (+, *N*3m(1)-CH$_3$), 13.7 (+, *N*3m(2)-CH$_3$). – **Analytical HPLC** (5–95% acetonitrile + 0.1% TFA in 30 min, detection at 218 nm): t$_{Ret}$ = 11.3 min (92%). – **MS** (m/z, MALDI-TOF): 429 [M+H]$^+$, 451 [M+Na]$^+$, 467 [M+K]$^+$.

***cyclo*-(L-Ile-D-Pro-*N*3m-L-Phe) (8e)**

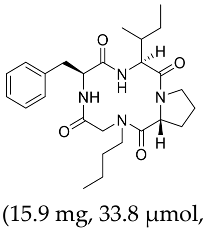

Fmoc-L-phenylalanine (310 mg, 800 μmol, 4.00 equiv.) was immobilized in accordance with **GP1**. The peptoid monomer *N*3m was incorporated following **GP4** using *n*-butylamine (158 μL, 117 mg, 1.60 mmol, 8.00 equiv.) as submonomer. Subsequently, Fmoc-D-proline (270 mg, 800 μmol, 4.00 equiv.) and Fmoc-L-isoleucine (283 mg, 800 μmol, 4.00 equiv.) were coupled following **GP3**. Cleavage was performed according to **GP5**. The crude linear precursor (48.8 mg, 100 μmol, 1.00 equiv.) was cyclized following **GP6**. After purification *via* preparative reversed phase HPLC (5–95% acetonitrile in water with 0.1% TFA in 45 min), the macrocycle was isolated as colorless solid (15.9 mg, 33.8 μmol, 29% over 10 steps).

The NMR spectra contain multiple signal sets. Chemical shifts of the most prominent isomers are given. Isomer "(1)" is represented in about twice the amount of isomer "(2)". **^{1}H-NMR** (500 MHz, DMSO-d_6, ppm): δ = 9.49 (d, *J* = 9.3 Hz, 1H, phe(2)-N$_{\alpha}$*H*), 7.56 (d, *J* = 7.8 Hz, 1H, ile(1)-N$_{\alpha}$*H*), 7.31–7.16 (m, 11H, ile(2)-N$_{\alpha}$*H*, 5 × phe(1)-C*H*, 5 × phe(2)-C*H*), 7.12 (d, *J* = 11.2 Hz, 1H, phe(1)-N$_{\alpha}$*H*), 4.98 (d, *J* = 8.5 Hz, 1H, pro(2)-C$_{\alpha}$*H*), 4.92 (d, *J* = 8.0 Hz, 1H, pro(1)-C$_{\alpha}$*H*), 4.90–4.84 (m, 1H, phe(2)-C$_{\alpha}$*H*), 4.44 (td, *J* = 10.4. 6.0 Hz, 1H, phe(1)-C$_{\alpha}$*H*), 4.09–4.03 (m, 2H, ile(1)-C$_{\alpha}$*H*, (2)-COC*H*HN), 3.81–3.37 (m, 2H, (2)-COCH*H*N, *N*3m(2)-C$_1$*H*H), 3.72–3.62 (m, 1H, *N*3m(1)-C$_1$*H*H), 3.61–3.52 (m, 2H, (1)-COC*H*$_2$N), 3.50–3.38 (m, 3H, pro(1)-C$_{\delta}$*H*H, pro(2)-C$_{\delta}$*H*$_2$), 3.36–3.16 (m, 4H, ile(2)-C$_{\alpha}$*H*, phe(2)-C*H*H, pro(1)-C$_{\delta}$H*H*, *N*3m(1)-C$_1$H*H*), 3.14–3.02 (m, 1H, *N*3m(2)-C$_1$H*H*), 2.93–2.82 (m, 2H, phe(1)-C*H*$_2$), 2.65–2.57 (m, 1H, phe(2)-CH*H*), 2.31–2.12 (m, 2H, pro(1)-C$_{\beta}$*H*H, pro(2)-C$_{\beta}$*H*H), 1.96–1.87 (m, 2H, ile(1)-C*H*, pro(1)-C$_{\beta}$H*H*), 1.86–1.79 (m, 2H, pro(2)-C$_{\gamma}$*H*$_2$), 1.78–1.72 (m, 2H, pro(2)-C$_{\beta}$H*H*, pro(1)-C$_{\gamma}$*H*H), 1.66–1.50 (m, 4H, ile(2)-C*H*, ile(1)-C*H*H, pro(1)-C$_{\gamma}$H*H*, *N*3m(1)-C$_2$*H*H), 1.48–1.40 (m, 2H, *N*3m(2)-C$_2$*H*$_2$), 1.36–1.28 (m, 3H, *N*3m(1)-C$_2$H*H*, *N*3m(1)-C$_3$*H*H, *N*3m(2)-C$_3$*H*H), 1.25–1.09 (m, 4H, ile(1)-CH*H*, ile(2)-C*H*H, *N*3m(1)-C$_3$H*H*, *N*3m(2)-C$_3$H*H*), 0.93 (t, *J* = 7.1 Hz, 3H, *N*3m(1)-C*H*$_3$), 0.89–0.84 (m, 6H, ile(1)-C*H*$_3$, *N*3m(2)-C*H*$_3$), 0.82 (d, *J* = 6.8 Hz, 3H, ile(1)-C*H*$_3$), 0.79–0.74 (m, 1H, ile(2)-CH*H*), 0.71 (t, *J* = 7.2 Hz, 3H, ile(2)-C*H*$_3$), 0.56 (d, *J* = 6.4 Hz, 3H, ile(2)-C*H*$_3$). – **^{13}C-NMR** (125 MHz, DMSO-d_6, ppm): δ = 172.3 (C$_q$, (2)-CON), 171.3 (C$_q$, (1)-CON), 170.9 (C$_q$, (1)-CON), 170.5 (C$_q$, (1)-CON), 170.3 (C$_q$, (2)-CON), 168.8 (C$_q$, (2)-CON), 168.3 (C$_q$, (1)-CON), 167.8 (C$_q$, (2)-CON),137.8 (C$_q$, phe(2)-C$_{ar}$), 137.7 (C$_q$, phe(1)-C$_{ar}$), 129.4 (+, 2 × phe(2)-CH), 128.7 (+, 2 × phe(1)-CH), 128.2 (+, 2 × phe(1)-CH), 128.0 (+, 2 × phe(2)-CH), 126.3 (+, phe(1)-CH), 126.1 (+, phe(2)-CH), 60.3 (+, ile(1)-C$_{\alpha}$H), 58.2 (+, ile(2)-C$_{\alpha}$H), 57.2 (+, pro(1)-C$_{\alpha}$H), 56.3 (+, phe(1)-C$_{\alpha}$H), 55.8 (+, pro(2)-C$_{\alpha}$H), 52.7 (–, (1)-COCH$_2$N), 50.6 (–, (2)-COCH$_2$N), 49.7 (*N*3m(1)-C$_1$H$_2$), 48.0 (*N*3m(2)-C$_1$H$_2$), 47.5 (–, pro(1)-C$_{\delta}$H$_2$), 47.1 (–, pro(2)-C$_{\delta}$H$_2$), 38.1 (+, ile(2)-CH), 37.3 (–, phe(2)-CH$_2$), 37.2 (–, phe(1)-CH$_2$), 33.5 (+, ile(1)-CH), 31.6 (–, pro(2)-C$_{\beta}$H$_2$), 31.0 (–, pro(1)-C$_{\beta}$H$_2$, *N*3m(1)-C$_2$H$_2$), 29.0 (–, *N*3m(1)-C$_2$H$_2$), 25.8 (–, ile(1)-CH$_2$), 24.4 (–, ile(2)-CH$_2$), 21.6 (–, pro(2)-C$_{\gamma}$H$_2$), 20.2 (–, pro(1)-C$_{\gamma}$H$_2$), 19.6 (–, *N*3m(2)-C$_3$H$_2$), 19.4 (–, *N*3m(1)-C$_3$H$_2$), 15.5 (+, ile(1)-CH$_3$), 15.4 (+, ile(2)-CH$_3$), 13.8 (+, *N*3m(2)-CH$_3$), 13.7 (+, *N*3m(1)-CH$_3$), 11.9 (+, ile(2)-CH$_3$), 9.56 (+, ile(1)-CH$_3$). – **Analytical HPLC** (5–95% acetonitrile + 0.1% TFA in 30 min, detection at 218 nm): t_{Ret} = 12.5 min (98%). – **MS** (m/z, MALDI-TOF): 471 [M+H]$^+$, 493 [M+Na]$^+$, 509 [M+K]$^+$.

***cyclo*-(L-Ile-D-Pro-*N*4m-L-Phe) (8f)**

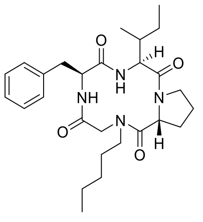

Fmoc-L-phenylalanine (310 mg, 800 μmol, 4.00 equiv.) was immobilized in accordance with **GP1**. The peptoid monomer *N*4m was incorporated following **GP4** using *n*-pentylamine (185 μL, 140 mg, 1.60 mmol, 8.00 equiv.) as submonomer. Subsequently, Fmoc-D-proline (270 mg, 800 μmol, 4.00 equiv.) and Fmoc-L-isoleucine (283 mg, 800 μmol, 4.00 equiv.) were coupled following **GP3**. Cleavage was performed according to **GP5**. The crude linear precursor (50.3 mg, 100 μmol, 1.00 equiv.) was cyclized following **GP6**. After purification *via* preparative reversed phase HPLC (5–95% acetonitrile in water with 0.1% TFA in 45 min), the macrocycle was isolated as colorless solid (25.2 mg, 52.0 μmol, 52% over 10 steps).

The NMR spectra contain multiple signal sets. Chemical shifts of the most prominent isomers are given. Isomers "(1)" and "(2)" are represented in a similar amount. Isomer "(3)" represents a minor conformation. **^{1}H-NMR** (500 MHz, acetonitrile-d_3, ppm): δ = 7.37–7.17 (m, 15H, 5 × phe(1)-C*H*, 5 × phe(2)-C*H*, 5 × phe(3)-C*H*), 6.76–6.66 (m, 1H, ile(2)-N$_{\alpha}$*H*), 6.50–6.40 (m, 2H, phe(2)-N$_{\alpha}$*H*, phe(3)-N$_{\alpha}$*H*), 6.35 (d, *J* = 9.2 Hz, 1H, phe(1)-N$_{\alpha}$*H*), 6.26 (d, *J* = 10.2 Hz, 1H, ile(1)-N$_{\alpha}$*H*), 5.87 (d, *J* = 10.8 Hz, 1H, ile(3)-N$_{\alpha}$*H*), 5.09 (dd, *J* = 7.5, 3.6 Hz, 1H, pro(1)-C$_{\alpha}$*H*), 5.03–4.91 (m, 1H, pro(3)-C$_{\alpha}$*H*), 4.59–4.52 (m, 1H, phe(2)-C$_{\alpha}$*H*), 4.51–4.41 (m, 3H, phe(1)-C$_{\alpha}$*H*, phe(3)-C$_{\alpha}$*H*, *N*4m(1)-C$_1$*H*H), 4.26 (t, *J* = 10.5 Hz, 1H, ile(2)-C$_{\alpha}$*H*), 4.10–4.03 (m, 1H, ile(1)-C$_{\alpha}$*H*), 3.91 (d, *J* = 15.6 Hz, 1H, (1)-COC*H*HN), 3.86–3.56 (m, 8H, pro(2)-C$_{\alpha}$*H*, pro(2)-C$_{\delta}$*H*H, pro(3)-C$_{\delta}$*H*$_2$, (2)-COC*H*$_2$N, (3)-COC*H*$_2$N), 3.55–3.48 (m, 2H, pro(1)-C$_{\delta}$*H*H, pro(2)-C$_{\delta}$H*H*), 3.46–3.35 (m, 5H, ile(3)-C$_{\alpha}$*H*, pro(1)-C$_{\delta}$H*H*, *N*4m(2)-C$_1$*H*H, *N*4m(3)-C$_1$*H*H, (1)-COCH*H*N), 3.30–3.15 (m, 2H, *N*4m(1)-C$_1$H*H*, phe(1)-C*H*H), 3.08–2.99 (m, 2H, phe(2)-C*H*H, phe(3)-C*H*H), 2.97–2.89 (m, 2H, phe(2)-CH*H*, phe(3)-C*H*H), 2.85–2.74 (m, 1H, phe(1)-CH*H*), 2.37–2.22 (m, 3H, pro(1)-C$_{\beta}$*H*H, pro(3)-C$_{\beta}$*H*H, pro(1)-C$_{\gamma}$*H*H), 2.18–1.97 (m, 6H, pro(1)-C$_{\beta}$H*H*, pro(2)-C$_{\beta}$*H*$_2$, pro(3)-C$_{\beta}$H*H*, pro(1)-C$_{\gamma}$H*H*, pro(2)-C$_{\gamma}$*H*H), 1.91–1.77 (m, 7H, ile(1)-C*H*, ile(2)-C*H*, ile(3)-C*H*, pro(2)-C$_{\gamma}$H*H*, pro(3)-C$_{\gamma}$*H*$_2$, *N*4m(2)-C$_4$*H*H), 1.72–1.58 (m, 2H, *N*4m(2)-C$_2$*H*H, *N*4m(2)-C$_4$H*H*), 1.56–1.14 (m, 21H, ile(1)-C*H*$_2$, ile(2)-C*H*$_2$, ile(3)-C*H*$_2$, *N*4m(1)-C$_2$*H*$_2$, *N*4m(2)-C$_2$*H*H, *N*4m(3)-C$_2$*H*$_2$, *N*4m(1)-C$_3$*H*$_2$, *N*4m(2)-C$_3$*H*$_2$, *N*4m(3)-C$_3$*H*$_2$, *N*4m(1)-C$_4$*H*$_2$, *N*4m(3)-C$_4$*H*$_2$), 1.05–0.78 (m, 25H, 2 × ile(1)-C*H*$_3$, 2 × ile(2)-C*H*$_3$, 2 × ile(3)-C*H*$_3$, *N*4m(1)-C*H*$_3$, *N*4m(2)-C*H*$_3$, *N*4m(3)-C*H*$_3$). – **^{13}C-NMR** (125 MHz, acetonitrile-d_3, ppm): δ = 179.5 (C$_q$, (3)-CON), 174.8 (C$_q$, (1)-CON), 174.4 (C$_q$, (1)-CON, (2)-CON), 172.9 (C$_q$, (3)-CON), 172.3 (C$_q$, (1)-CON, 2 × (2)-CON), 169.9 (C$_q$, (2)-CON), 169.7 (C$_q$, (1)-CON), 168.0 (C$_q$, (3)-CON), 167.8 (C$_q$, (3)-CON), 139.6 (C$_q$, phe(2)-C$_{ar}$), 138.6 (C$_q$, phe(1)-C$_{ar}$), 137.9 (C$_q$, phe(3)-C$_{ar}$), 130.7 (+, 2 × phe(2)-CH), 130.0 (+, 2 × phe(1)-CH, 2 × phe(3)-CH), 129.4 (+, 2 × phe(2)-CH, 2 × phe(3)-CH), 128.9 (+, 2 × phe(1)-CH), 127.7 (+, phe(3)-CH), 127.5 (+, phe(2)-CH), 127.0 (+, phe(1)-CH), 65.0 (+, phe(3)-C$_{\alpha}$H), 62.4 (+, ile(1)-

C_αH), 59.8 (+, ile(3)-C_αH), 59.7 (+, pro(2)-C_αH, ile(2)-C_αH), 58.7 (+, phe(1)-C_αH), 58.1 (+, pro(3)-C_αH), 57.6 (+, phe(2)-C_αH), 55.3 (+, pro(1)-C_αH), 53.7 (–, (1)-$COCH_2N$), 51.5 (–, *N*4m(1)-C_1H_2), 50.5 (–, (2)-$COCH_2N$, (3)-$COCH_2N$), 48.7 (–, pro(1)-$C_\delta H_2$), 47.8 (–, pro(2)-$C_\delta H_2$), 46.8 (–, pro(3)-$C_\delta H_2$), 38.3 (–, *N*4m(2)-C_1H_2, *N*4m(3)-C_1H_2), 37.9 (–, phe(3)-CH_2), 37.5 (–, phe(1)-CH_2, phe(2)-CH_2), 35.4 (+, ile(3)-CH), 35.3 (+, ile(1)-CH, ile(2)-CH), 32.0 (–, pro(1)-$C_\beta H_2$, pro(3)-$C_\beta H_2$), 30.7 (–, *N*4m(2)-C_2H_2), 29.8 (–, *N*4m(1)-C_2H_2), 29.3 (–, *N*4m(3)-C_2H_2), 28.1 (–, pro(2)-$C_\beta H_2$), 26.9 (–, *N*4m(1)-C_3H_2), 26.1 (–, pro(3)-$C_\gamma H_2$), 25.8 (–, pro(1)-$C_\gamma H_2$), 25.7 (–, pro(2)-$C_\gamma H_2$), 23.2 (–, ile(1)-CH_2), 23.1 (–, ile(2)-CH_2, ile(3)-CH_2), 23.0 (–, *N*4m(1)-C_4H_2), 21.3 (–, *N*4m(2)-C_4H_2, *N*4m(3)-C_4H_2), 16.8 (+, ile(3)-CH_3), 15.8 (+, ile(1)-CH_3), 15.5 (+, ile(2)-CH_3), 14.3 (+, *N*4m(1)-CH_3), 14.2 (+, *N*4m(2)-CH_3), 12.3 (+, *N*4m(3)-CH_3), 11.5 (+, ile(3)-CH_3), 10.7 (+, ile(2)-CH_3), 10.5 (+, ile(1)-CH_3). – **Analytical HPLC** (5–95% acetonitrile + 0.1% TFA in 30 min, detection at 218 nm): t_{Ret} = 12.7 min (>99%). – **MS** (m/z, MALDI-TOF): 485 $[M+H]^+$, 507 $[M+Na]^+$, 523 $[M+K]^+$.

***cyclo*-(L-Ile-D-Pro-*N*4m-L-Trp) (8g)**

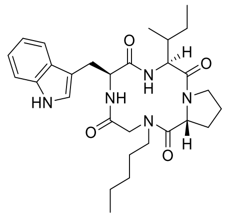

Fmoc-L-tryptophan (341 mg, 800 µmol, 4.00 equiv.) was immobilized in accordance with **GP1**. The peptoid monomer *N*4m was incorporated following **GP4** using *n*-pentylamine (185 µL, 140 mg, 1.60 mmol, 8.00 equiv.) as submonomer. Subsequently, Fmoc-D-proline (270 mg, 800 µmol, 4.00 equiv.) and Fmoc-L-isoleucine (283 mg, 800 µmol, 4.00 equiv.) were coupled following **GP3**. Cleavage was performed according to **GP5**. The crude linear precursor (51.9 mg, 100 µmol, 1.00 equiv.) was cyclized following **GP6**. After purification *via* preparative reversed phase HPLC (5–95% acetonitrile in water with 0.1% TFA in 45 min), the macrocycle was isolated as colorless solid (27.4 mg, 52.3 µmol, 42% over 10 steps).

The NMR spectra contain multiple signal sets. Chemical shifts of the most prominent isomers are given. Isomers "(1)"and "(2)" are represented in a similar amount. **^{1}H-NMR** (500 MHz, acetonitrile-d_3, ppm): δ = 9.21 (s, 1H, trp(1)-N*H*), 9.12 (s, 1H, trp(2)-N*H*), 7.60 (d, *J* = 7.8 Hz, 1H, trp(2)-C*H*), 7.58 (d, *J* = 7.8 Hz, 1H, trp(1)-C*H*), 7.42–7.34 (m, 3H, trp(1)-C*H*, trp(2)-C*H*, trp(2)-N_α*H*), 7.17–6.99 (m, 6H, 3 × trp(1)-C*H*, 3 × trp(2)-C*H*), 6.75–6.63 (m, 1H, ile(2)-N_α*H*), 6.43 (d, *J* = 9.3 Hz, 1H, trp(1)-N_α*H*), 6.25 (d, *J* = 10.1 Hz, 1H, ile(1)-N_α*H*), 5.06 (dd, *J* = 7.5, 3.7 Hz, 1H, pro(1)-C_α*H*), 4.92–4.85 (m, 1H, trp(2)-C_α*H*), 4.76–4.59 (m, 2H, pro(2)-C_α*H*, trp(1)-C_α*H*), 4.49–4.29 (m, 1H, *N*4m(1)-C_1*H*H), 4.23 (t, *J* = 10.5 Hz, 1H, ile(1)-C_α*H*), 4.08–4.01 (m, 1H, ile(2)-C_α*H*), 3.93 (d, *J* = 15.4 Hz, 1H, (1)-COC*H*HN), 3.68–3.51 (m, 3H, pro(1)-C_δ*H*H, pro(2)-$C_\delta H_2$), 3.48–3.26 (m, 6H, pro(1)-C_δH*H*, trp(1)-C*H*H, *N*4m(2)-C_1H_2, (1)-COCH*H*N, (2)-$COCH_2N$), 3.26–3.02 (m, 4H, trp(1)-CH*H*, trp(2)-CH_2, *N*4m(1)-C_1H*H*), 2.37–2.21 (m, 2H, pro(2)-C_β*H*H, pro(2)-C_γ*H*H), 2.03–1.97 (m, 3H, pro(1)-$C_\beta H_2$, pro(2)-C_βH*H*), 1.89–1.78 (m, 4H, ile(1)-C*H*, ile(1)-C*H*, pro(1)-C_γ*H*H, pro(2)-C_γH*H*), 1.73–1.54 (m, 2H, pro(1)-C_γH*H*, *N*4m(2)-C_2*H*H), 1.36–1.06 (m, 13H, ile(1)-C*H*H, ile(2)-C*H*H, *N*4m(1)-C_2H_2, *N*4m(2)-C_2H*H*, *N*4m(1)-C_3H_2, *N*4m(2)-C_3H_2, *N*4m(1)-C_4H_2, *N*4m(2)-C_4H_2), 0.96–0.80 (m, 20H, ile(1)-CH*H*, ile(2)-CH*H*, 2 × ile(1)-CH_3, 2 × ile(2)-CH_3, *N*4m(1)-CH_3, *N*4m(2)-CH_3). – **^{13}C-NMR** (125 MHz, acetonitrile-d_3, ppm): δ = 174.8 (C_q, (1)-CON), 174.5 (C_q, (2)-CON), 174.1 (C_q, (1)-CON), 173.1 (C_q, (2)-CON), 172.0 (C_q, (1)-CON, (2)-CON), 169.7 (C_q, (1)-CON, (2)-CON), 137.0 (C_q, trp(1)-C_{ar}), 136.7 (C_q, trp(2)-C_{ar}), 128.7 (C_q, trp(2)-C_{ar}), 127.9 (C_q, trp(1)-C_{ar}), 124.4 (+, trp(2)-CH), 123.9 (+, trp(1)-CH), 122.0 (+, trp(2)-CH), 121.8 (+, trp(1)-CH), 119.6 (+, trp(1)-CH), 119.1 (+, trp(2)-CH), 119.0 (+, trp(2)-CH), 118.9 (+, trp(1)-CH), 112.0 (C_q, trp(2)-C_{ar}), 111.9 (+, trp(2)-CH), 111.8 (+, trp(1)-CH), 110.2 (C_q, trp(1)-C_{ar}), 59.4 (+, ile(1)-C_αH), 57.3 (+, trp(1)-C_αH), 57.2 (+, ile(2)-C_αH), 55.0 (+, pro(1)-C_αH), 54.7 (+, pro(2)-C_αH), 53.3 (–, (1)-$COCH_2N$), 51.1 (–, *N*4m(1)-C_1H_2), 50.8 (–, *N*4m(2)-C_1H_2), 50.0 (–, (2)-$COCH_2N$), 48.5 (–, pro(1)-$C_\delta H_2$), 47.4 (–, pro(2)-$C_\delta H_2$), 37.4 (+, ile(2)-CH), 35.1 (+, ile(1)-CH), 31.7 (–, pro(2)-$C_\beta H_2$), 30.3 (–, *N*4m(2)-C_2H_2), 29.0 (–, *N*4m(1)-C_2H_2), 28.9 (–, pro(1)-$C_\beta H_2$), 28.1 (–, trp(2)-CH_2), 27.7 (–, trp(1)-CH_2), 27.0 (–, *N*4m(1)-C_3H_2), 26.6 (–, *N*4m(2)-C_3H_2), 25.5 (–, ile(1)-CH_2), 25.4 (–, pro(2)-$C_\gamma H_2$), 22.8 (–, *N*4m(1)-C_4H_2), 22.6 (–, *N*4m(2)-C_4H_2), 22.5 (–, ile(2)-CH_2, pro(1)-$C_\gamma H_2$), 15.3 (+, ile(1)-CH_3), 15.0 (+, ile(2)-CH_3), 13.9 (+, *N*4m(1)-CH_3), 13.8 (+, *N*4m(2)-CH_3), 10.4 (+, ile(2)-CH_3), 10.2 (+, ile(1)-CH_3). – **Analytical HPLC** (5–95% acetonitrile + 0.1% TFA in 30 min, detection at 218 nm): t_{Ret} = 12.9 min (>99%). – **MS** (m/z, MALDI-TOF): 524 $[M+H]^+$, 546 $[M+Na]^+$, 562 $[M+K]^+$.

***cyclo*-(L-Phe-D-Pro-*N*4m-L-Phe) (8h)**

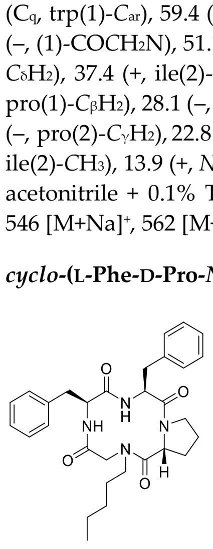

Fmoc-L-phenylalanine (310 mg, 800 µmol, 4.00 equiv.) was immobilized in accordance with **GP1**. The peptoid monomer *N*4m was incorporated following **GP4** using *n*-pentylamine (185 µL, 140 mg, 1.60 mmol, 8.00 equiv.) as submonomer. Subsequently, Fmoc-D-proline (270 mg, 800 µmol, 4.00 equiv.) and Fmoc-L-phenylalanine (310 mg, 800 µmol, 4.00 equiv.) were coupled following **GP3**. Cleavage was performed according to **GP5**. The crude linear precursor (53.7 mg, 100 µmol, 1.00 equiv.) was cyclized following **GP6**. After purification *via* preparative reversed phase HPLC (5–95% acetonitrile in water with 0.1% TFA in 45 min), the macrocycle was isolated as colorless solid (25.2 mg, 48.6 µmol, 39% over 10 steps).

The NMR spectra contain multiple signal sets. Chemical shifts of the most prominent isomer are given. **^{1}H-NMR** (500 MHz, acetonitrile-d_3, ppm): δ = 8.65–8.48 (m, 1H, phe-N_α*H*), 7.92–7.81 (m, 1H, phe-N_α*H*), 7.63–7.29 (m, 10H, 10 × phe-C*H*), 5.00–4.86 (m, 1H, phe-C_α*H*), 4.83–4.69 (m, 1H, pro-C_α*H*), 4.19–4.09 (m, 1H, phe-C_α*H*), 4.02–3.92 (m, 1H, COC*H*HN), 3.91–3.81 (m, 1H, *N*4m-C_1*H*H), 3.79–3.68 (m, 3H, pro-$C_\delta H_2$, COCH*H*N), 3.39–3.31 (m, 1H, phe-C*H*H), 3.26–3.16 (m, 1H, phe-CH*H*), 2.95–2.89 (m, 2H, phe-CH_2), 2.87–2.67 (m, 1H, *N*4m-C_1H*H*), 2.60–2.48 (m, 1H, pro-C_β*H*H), 2.35–2.17 (m, 2H, pro-C_βH*H*, pro-C_γ*H*H), 2.08–2.00 (m, 1H, pro-C_γH*H*), 1.77–1.61 (m, 2H, *N*4m-C_2H_2), 1.56–1.45 (m, 2H, *N*4m-C_4H_2), 1.46–1.38 (m, 2H, *N*4m-C_3H_2), 1.11 (t, *J* = 7.2 Hz, 3H, *N*4m-CH_3). – **^{13}C-NMR** (125 MHz, acetonitrile-d_3, ppm): δ = 173.0 (C_q, CON),

172.8 (C$_q$, CON), 168.2 (C$_q$, 2 × CON), 139.6 (C$_q$, phe-C$_{ar}$), 137.7 (C$_q$, phe-C$_{ar}$), 130.8 (+, 2 × phe-CH), 130.7 (+, 2 × phe-CH), 129.6 (+, 2 × phe-CH), 128.8 (+, 2 × phe-CH), 127.9 (+, phe-CH), 127.0 (+, phe-CH), 65.3 (+, pro-C$_\alpha$H), 57.2 (+, phe-C$_\alpha$H), 51.8 (+, phe-C$_\alpha$H), 50.2 (–, 2C, COCH$_2$N, *N*4m-C$_1$H$_2$), 47.3 (–, pro-C$_\delta$H$_2$), 38.2 (–, phe-CH$_2$), 38.1 (–, phe-CH$_2$), 29.8 (–, *N*4m-C$_2$H$_2$), 27.1 (–, *N*4m-C$_3$H$_2$), 26.7 (–, pro-C$_\beta$H$_2$), 23.1 (–, *N*4m-C$_4$H$_2$), 23.0 (–, pro-C$_\gamma$H$_2$), 14.3 (+, *N*4m-CH$_3$). – **Analytical HPLC** (5–95% acetonitrile + 0.1% TFA in 30 min, detection at 218 nm): t$_{Ret}$ = 13.0 min (>99%). – **MS** (m/z, MALDI-TOF): 519 [M+H]$^+$, 541 [M+Na]$^+$, 557 [M+K]$^+$.

***cyclo*-(L-Phe-D-Pro-*N*4m-L-Trp) (8i)**

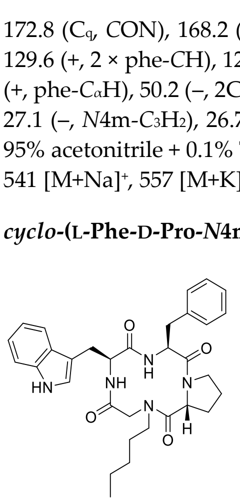

Fmoc-L-tryptophan (341 mg, 800 µmol, 4.00 equiv.) was immobilized in accordance with **GP1**. The peptoid monomer *N*4m was incorporated following **GP4** using *n*-pentylamine (185 µL, 140 mg, 1.60 mmol, 8.00 equiv.) as submonomer. Subsequently, Fmoc-D-proline (270 mg, 800 µmol, 4.00 equiv.) and Fmoc-L-phenylalanine (310 mg, 800 µmol, 4.00 equiv.) were coupled following **GP3**. Cleavage was performed according to **GP5**. The crude linear precursor (57.7 mg, 100 µmol, 1.00 equiv.) was cyclized following **GP6**. After purification *via* preparative reversed phase HPLC (5–95% acetonitrile in water with 0.1% TFA in 45 min), the macrocycle was isolated as colorless solid (20.4 mg, 36.6 µmol, 18% over 10 steps).

The NMR spectra contain multiple signal sets. Chemical shifts of the most prominent isomers are given. Isomer "(1)" is represented in about three times the amount of isomer "(2)". **^{1}H-NMR** (500 MHz, acetonitrile-*d*$_3$, ppm): δ = 9.55 (s, 1H, trp(1)-N*H*), 9.40–9.34 (m, 1H, trp(2)-N*H*), 7.65 (d, *J* = 6.8 Hz, 1H, phe(1)-N$_\alpha$*H*), 7.62–7.48 (m, 3H, trp(1)-C*H*, trp(2)-C*H*, trp(2)-N$_\alpha$*H*), 7.40–7.30 (m, 4H, 2 × trp(1)-C*H*, 2 × trp(2)-C*H*), 7.29–7.17 (m, 8H, 3 × phe(1)-C*H*, 3 × phe(2)-C*H*, trp(1)-N$_\alpha$*H*, phe(2)-N$_\alpha$*H*), 7.17–7.08 (m, 6H, 2 × phe(1)-C*H*, 2 × phe(2)-C*H*, trp(1)-C*H*, trp(2)-C*H*), 7.04 (t, *J* = 7.5 Hz, 2H, trp(1)-C*H*, trp(2)-C*H*), 4.75–4.68 (m, 2H, phe(1)-C$_\alpha$*H*, phe(2)-C$_\alpha$*H*), 4.66 (dd, *J* = 8.7, 3.6 Hz, 1H, pro(1)-C$_\alpha$*H*), 4.48 (dd, *J* = 8.3, 5.7 Hz, 1H, pro(2)-C$_\alpha$*H*), 4.39 (dd, *J* = 8.9, 6.1 Hz, 1H, trp(2)-C$_\alpha$*H*), 4.29–4.20 (m, 1H, trp(1)-C$_\alpha$*H*), 4.19–4.14 (m, 1H, (2)-COC*H*HN), 4.04 (d, *J* = 17.1 Hz, 1H, (1)-COC*H*HN), 3.91 (d, *J* = 17.1 Hz, 1H, (1)-COCH*H*N), 3.53–3.45 (m, 1H, *N*4m(1)-C$_1$*H*H), 3.42–3.16 (m, 10H, pro(1)-C$_\delta$*H*$_2$, pro(2)-C$_\delta$*H*$_2$, trp(1)-C*H*$_2$, trp(2)-C*H*$_2$, *N*4m(2)-C$_1$*H*H, (2)-COCH*H*N), 3.16–3.07 (m, 1H, phe(2)-C*H*H), 3.05–2.99 (m, 1H, phe(2)-CH*H*), 2.97–2.82 (m, 3H, phe(1)-C*H*$_2$, *N*4m(1)-C$_1$H*H*), 2.74–2.65 (m, 1H, *N*4m(2)-C$_1$H*H*), 2.01–1.88 (m, 2H, pro(1)-C$_\beta$*H*H, pro(2)-C$_\beta$*H*H), 1.86–1.73 (m, 2H, pro(1)-C$_\beta$H*H*, pro(2)-C$_\beta$H*H*, *N*4m(1)-C$_2$*H*H), 1.71–1.65 (m, 1H, pro(1)-C$_\gamma$*H*H), 1.62–1.51 (m, 2H, pro(1)-C$_\gamma$H*H*, *N*4m(1)-C$_2$H*H*), 1.49–1.40 (m, 2H, pro(2)-C$_\gamma$*H*$_2$), 1.37–1.26 (m, 4H, *N*4m(2)-C$_2$*H*$_2$, *N*4m(1)-C$_3$*H*H, *N*4m(1)-C$_4$*H*H), 1.26–1.11 (m, 4H, *N*4m(2)-C$_3$*H*H, *N*4m(1)-C$_3$H*H*, *N*4m(1)-C$_4$H*H*, *N*4m(2)-C$_4$*H*H), 0.93–0.89 (m, 5H, *N*4m(2)-C$_3$H*H*, *N*4m(2)-C$_4$H*H*, *N*4m(1)-C*H*$_3$), 0.76 (t, *J* = 7.1 Hz, 3H, *N*4m(2)-C*H*$_3$). – **^{13}C-NMR** (125 MHz, acetonitrile-*d*$_3$, ppm): δ = 173.8 (C$_q$, (2)-CON), 173.7 (C$_q$, (1)-CON), 171.9 (C$_q$, (1)-CON), 169.8 (C$_q$, (1)-CON), 169.2 (C$_q$, (1)-CON), 169.1 (C$_q$, (2)-CON), 168.9 (C$_q$, (2)-CON), 167.7 (C$_q$, (2)-CON), 137.4 (C$_q$, phe(1)-C$_{ar}$), 137.3 (C$_q$, phe(2)-C$_{ar}$), 137.2 (C$_q$, trp(1)-C$_{ar}$), 135.1 (C$_q$, trp(2)-C$_{ar}$), 131.1 (C$_q$, trp(2)-C$_{ar}$), 130.6 (+, 2 × phe(2)-CH), 130.3 (+, 2 × phe(1)-CH), 129.8 (+, 2 × phe(2)-CH), 129.4 (+, 2 × phe(1)-CH), 128.7 (C$_q$, trp(1)-C$_{ar}$), 128.1 (+, phe(1)-CH), 127.9 (+, phe(2)-CH), 126.6 (+, trp(1)-CH), 124.8 (+, trp(2)-CH), 122.6 (+, trp(1)-CH), 122.2 (+, trp(2)-CH), 120.2 (+, trp(1)-CH), 119.7 (+, trp(2)-CH), 119.2 (+, trp(1)-CH), 118.9 (+, trp(2)-CH), 112.5 (+, trp(1)-CH), 112.3 (+, trp(2)-CH), 111.3 (C$_q$, trp(2)-C$_{ar}$), 107.3 (C$_q$, trp(1)-C$_{ar}$), 57.9 (+, pro(1)-C$_\alpha$H), 57.4 (+, pro(2)-C$_\alpha$H), 55.0 (+, phe(2)-C$_\alpha$H), 54.5 (+, trp(1)-C$_\alpha$H), 54.2 (+, phe(1)-C$_\alpha$H), 53.8 (+, trp(2)-C$_\alpha$H), 50.2 (–, (2)-COCH$_2$N), 50.0 (–, pro(1)-C$_\delta$H$_2$), 49.6 (–, pro(2)-C$_\delta$H$_2$), 49.5 (–, (1)-COCH$_2$N), 48.3 (–, *N*4m(2)-C$_1$H$_2$), 48.1 (–, *N*4m(1)-C$_1$H$_2$), 38.5 (–, phe(1)-CH$_2$), 37.8 (–, phe(2)-CH$_2$), 29.8 (–, pro(1)-C$_\beta$H$_2$), 29.7 (–, pro(2)-C$_\beta$H$_2$), 29.3 (–, *N*4m(1)-C$_3$H$_2$), 29.2 (–, *N*4m(2)-C$_3$H$_2$), 29.0 (–, pro(1)-C$_\gamma$H$_2$), 28.1 (–, pro(2)-C$_\gamma$H$_2$), 27.6 (–, trp(1)-CH$_2$), 27.5 (–, trp(2)-CH$_2$), 25.5 (–, *N*4m(2)-C$_2$H$_2$), 25.0 (–, *N*4m(1)-C$_2$H$_2$), 23.1 (–, *N*4m(1)-C$_4$H$_2$), 22.9 (–, *N*4m(2)-C$_4$H$_2$), 14.3 (+, *N*4m(1)-CH$_3$), 14.2 (+, *N*4m(2)-CH$_3$). – **Analytical HPLC** (5–95% acetonitrile + 0.1% TFA in 30 min, detection at 218 nm): t$_{Ret}$ = 11.0 min (>99%). – **MS** (m/z, MALDI-TOF): 558 [M+H]$^+$.

***cyclo*-(*N*ch-D-Pro-*N*1al-L-Phe) (9a)**

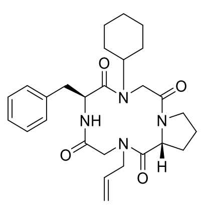

Fmoc-L-phenylalanine (310 mg, 800 µmol, 4.00 equiv.) was immobilized in accordance with **GP1**. The peptoid monomer *N*1al was incorporated following **GP4** using allylamine (120 µL, 91.4 mg, 1.60 mmol, 8.00 equiv.) as submonomer. Subsequently, Fmoc-D-proline (270 mg, 800 µmol, 4.00 equiv.) was coupled following **GP3**. The peptoid monomer *N*ch was incorporated following **GP4** using cyclohexylamine (184 µL, 159 mg, 1.60 mmol, 8.00 equiv.) as submonomer. Cleavage was performed according to **GP5**. The crude linear precursor (49.7 mg, 100 µmol, 1.00 equiv.) was cyclized following **GP6**. After purification *via* preparative reversed phase HPLC (5–95% acetonitrile in water with 0.1% TFA in 45 min), the macrocycle was isolated as colorless solid (24.7 mg, 51.4 µmol, 50% over 10 steps).

^{1}H-NMR (500 MHz, acetonitrile-*d*$_3$, ppm): δ = 7.30–7.07 (m, 5H, 5 × phe-C$_{ar}$*H*), 6.67 (d, *J* = 10.3 Hz, 1H, phe-N$_\alpha$*H*), 5.77–5.60 (m, 1H, *N*1al-C*H*), 5.15 (d, *J* = 10.3 Hz, 1H, *N*1al-C$_3$*H*H), 5.07 (d, *J* = 17.2 Hz, 1H, *N*1al-C$_3$H*H*), 5.02–4.92 (m, 1H, phe-C$_\alpha$*H*), 4.47 (d, *J* = 15.3 Hz, 1H, *N*1al-C$_1$*H*H), 4.42–4.36 (m, 1H, pro-C$_\alpha$*H*), 4.34–4.25 (m, 1H, *N*ch-C*H*), 3.83 (d, *J* = 18.3 Hz, 1H, COC*H*HN), 3.74–3.61 (m, 2H, COC*H*HN, COCH*H*N), 3.52–3.44 (m, 1H, pro-C$_\delta$*H*H), 3.30–3.09 (m, 4H, phe-C$_\beta$*H*H, pro-C$_\delta$H*H*, COCH*H*N, *N*1al-C$_1$H*H*), 2.85–2.80 (m, 1H, phe-C$_\beta$H*H*), 2.32–2.23 (m, 1H, pro-C$_\beta$*H*H), 2.09–2.02 (m, 1H, pro-C$_\gamma$*H*H), 1.91–1.79 (m, 3H, pro-C$_\beta$H*H*, pro-C$_\gamma$H*H*, *N*ch-C*H*HCH), 1.79–1.71 (m, 2H, 2 × *N*ch-C*H*H), 1.65–1.53 (m, 2H, *N*ch-C*H*H, *N*ch-C*H*HCH), 1.36–1.26 (m, 3H, 2 × *N*ch-CH*H*, *N*ch-CH*H*CH), 1.25–1.17 (m, 1H, *N*ch-CH*H*CH), 1.12–1.01 (m, 1H, *N*ch-CH*H*). – **^{13}C-NMR** (125 MHz, acetonitrile-*d*$_3$, ppm): δ = 173.1 (C$_q$, CON), 171.2 (C$_q$, CON), 170.2 (C$_q$, CON), 166.6 (C$_q$, CON), 139.7 (C$_q$, phe-C$_{ar}$), 133.4 (+, *N*1al-CH), 130.8 (+, 2 × phe-CH), 128.8 (+, 2 × phe-CH), 127.0 (+, phe-CH), 118.3 (–, *N*1al-C$_3$H$_2$), 64.6

(+, pro-C$_{\alpha}$H), 55.7 (+, *N*ch-CH), 53.0 (+, phe-C$_{\alpha}$H), 52.6 (–, *N*1al-C$_{1}$H$_{2}$), 50.9 (–, COCH$_{2}$N), 46.4 (–, pro-C$_{\delta}$H$_{2}$), 45.8 (–, COCH$_{2}$N), 38.6 (–, phe-C$_{\beta}$H$_{2}$), 31.2 (–, *N*ch-CH$_{2}$CH), 30.5 (–, *N*ch-CH$_{2}$CH), 27.8 (–, pro-C$_{\beta}$H$_{2}$), 26.7 (–, *N*ch-CH$_{2}$), 26.6 (–, *N*ch-CH$_{2}$), 26.4 (–, *N*ch-CH$_{2}$), 26.0 (–, pro-C$_{\gamma}$H$_{2}$). – **Analytical HPLC** (5–95% acetonitrile + 0.1% TFA in 30 min, detection at 218 nm): t$_{Ret}$ = 13.1 min (98%). – **MS** (m/z, MALDI-TOF): 482 [M+H]$^{+}$, 504 [M+Na]$^{+}$, 520 [M+K]$^{+}$.

***cyclo*-(*N*ch-D-Pro-*N*3mo-L-Trp) (9b)**

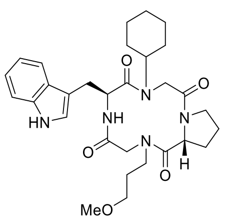

Fmoc-L-tryptophan (341 mg, 800 µmol, 4.00 equiv.) was immobilized following **GP1**. The peptoid monomer *N*3mo was incorporated following **GP4** using 3-methoxypropylamine (164 µL, 143 mg, 1.60 mmol, 8.00 equiv.) as submonomer. Subsequently, Fmoc-D-proline (270 mg, 800 µmol, 4.00 equiv.) was coupled following **GP3**. The peptoid monomer *N*ch was incorporated following **GP4** using cyclohexylamine (184 µL, 159 mg, 1.60 mmol, 8.00 equiv.) as submonomer. Cleavage was performed according to **GP5**. The crude linear precursor (57.0 mg, 100 µmol, 1.00 equiv.) was cyclized following **GP6**. After purification *via* preparative reversed-phase HPLC (5–95% acetonitrile in water with 0.1% TFA in 45 min), the macrocycle was isolated as colorless oil (13.8 mg, 25.0 µmol, 14% over 10 steps).

^{1}H-NMR (500 MHz, acetonitrile-*d*$_{3}$, ppm): δ = 9.04 (s, 1H, trp-N$_{ar}$*H*), 7.60 (d, *J* = 7.9 Hz, 1H, trp-C$_{ar}$*H*), 7.34 (d, *J* = 8.1 Hz, 1H, trp-C$_{ar}$*H*), 7.11–6.98 (m, 3H, 3 × trp-C$_{ar}$*H*), 6.66 (d, *J* = 10.1 Hz, 1H, trp-N$_{\alpha}$*H*), 5.10–4.94 (m, 1H, trp-C$_{\alpha}$*H*), 4.39–4.21 (m, 2H, pro-C$_{\alpha}$*H*, *N*ch-C*H*), 3.80 (d, *J* = 18.2 Hz, 1H, COC*H*HN), 3.73–3.65 (m, 2H, COC*H*HN, COCH*H*N), 3.55–3.49 (m, 1H, *N*3mo-C$_{1}$*H*H), 3.49–3.42 (m, 1H, pro-C$_{\delta}$*H*H), 3.37–3.31 (m, 2H, trp-C$_{\beta}$*H*H, COCH*H*N), 3.30–3.25 (m, 2H, *N*3mo-C$_{3}$*H*$_{2}$), 3.24 (s, 3H, *N*3mo-C*H*$_{3}$), 3.21–3.15 (m, 1H, pro-C$_{\delta}$H*H*), 3.03–2.95 (m, 1H, trp-C$_{\beta}$H*H*), 2.61–2.54 (m, 1H, *N*3mo-C$_{1}$H*H*), 2.27–2.17 (m, 1H, pro-C$_{\beta}$*H*H), 2.08–1.99 (m, 1H, pro-C$_{\gamma}$*H*H), 1.91–1.81 (m, 3H, pro-C$_{\beta}$H*H*, pro-C$_{\gamma}$H*H*, *N*ch-C*H*HCH), 1.78–1.71 (m, 2H, 2 × *N*ch-C*H*H), 1.64–1.51 (m, 4H, *N*ch-C*H*H, *N*ch-C*H*HCH, *N*3mo-C$_{2}$*H*$_{2}$), 1.36–1.26 (m, 3H, 2 × *N*ch-CH*H*, *N*ch-CH*H*CH), 1.22–1.13 (m, 1H, *N*ch-CH*H*CH), 1.10–1.00 (m, 1H, *N*ch-CH*H*). – **^{13}C-NMR** (125 MHz, acetonitrile-*d*$_{3}$, ppm): δ = 173.1 (C$_{q}$, *C*ON), 171.1 (C$_{q}$, *C*ON), 170.4 (C$_{q}$, *C*ON), 167.1 (C$_{q}$, *C*ON), 137.1 (C$_{q}$, trp-*C*$_{ar}$), 129.1 (C$_{q}$, trp-*C*$_{ar}$), 124.8 (+, trp-*C*$_{ar}$H), 122.1 (+, trp-*C*$_{ar}$H), 119.6 (+, trp-*C*$_{ar}$H), 119.5 (+, trp-*C*$_{ar}$H), 112.3 (+, trp-*C*$_{ar}$H), 112.1 (C$_{q}$, trp-*C*$_{ar}$), 70.8 (–, *N*3mo-C$_{3}$H$_{2}$), 64.8 (+, pro-*C*$_{\alpha}$H), 58.6 (+, *N*3mo-CH$_{3}$), 55.6 (+, *N*ch-CH), 52.5 (+, trp-C$_{\alpha}$H), 52.3 (–, COCH$_{2}$N), 48.1 (–, *N*3mo-C$_{1}$H$_{2}$), 46.3 (–, pro-C$_{\delta}$H$_{2}$), 45.9 (–, COCH$_{2}$N), 31.3 (–, *N*ch-CH$_{2}$CH), 30.6 (–, *N*ch-CH$_{2}$CH), 28.0 (–, trp-C$_{\beta}$H$_{2}$), 27.6 (–, pro-C$_{\beta}$H$_{2}$), 26.7 (–, *N*3mo-C$_{2}$H$_{2}$), 26.4 (–, 3C, 3 × *N*ch-CH$_{2}$), 26.0 (–, pro-C$_{\gamma}$H$_{2}$). – **Analytical HPLC** (5–95% acetonitrile + 0.1% TFA in 30 min, detection at 218 nm): t$_{Ret}$ = 12.6 min (92%). – **MS** (m/z, MALDI-TOF): 552 [M+H]$^{+}$, 574 [M+Na]$^{+}$, 590 [M+K]$^{+}$.

***cyclo*-(*N*1ay-D-Pro-*N*1ph-L-Trp) (9c)**

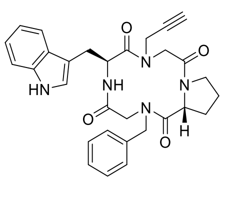

Fmoc-L-tryptophan (341 mg, 800 µmol, 4.00 equiv.) was immobilized in accordance with **GP1**. The peptoid monomer *N*1ph was incorporated following **GP4** using benzylamine (158 µL, 117 mg, 1.60 mmol, 8.00 equiv.) as submonomer. Subsequently, Fmoc-D-proline (270 mg, 800 µmol, 4.00 equiv.) was coupled following **GP3**. The peptoid monomer *N*1ay was incorporated following **GP4** using propargylamine (102 µL, 88.1 mg, 1.60 mmol, 8.00 equiv.) as submonomer. Cleavage was performed according to **GP5**. The crude linear precursor (54.4 mg, 100 µmol, 1.00 equiv.) was cyclized following **GP6**. After purification *via* preparative reversed phase HPLC (5–95% acetonitrile in water with 0.1% TFA in 45 min), the macrocycle was isolated as colorless solid (14.5 mg, 27.6 µmol, 18% over 10 steps).

^{1}H-NMR (500 MHz acetonitrile-*d*$_{3}$, ppm): δ = 9.08 (s, 1H, trp-N*H*), 7.63 (d, *J* = 7.9 Hz, 1H, trp-C*H*), 7.38–7.23 (m, 4H, 4 × *N*1ph-C*H*), 7.16–6.99 (m, 5H, 4 × trp-C*H*, *N*1ph-C*H*), 6.48 (d, *J* = 10.2 Hz, 1H, trp-N$_{\alpha}$*H*), 5.06 (q, *J* = 8.0 Hz, 1H, trp-C$_{\alpha}$*H*), 4.97 (d, *J* = 17.6 Hz, 1H, *N*1ay-C$_{1}$*H*H), 4.87 (d, *J* = 15.2 Hz, 1H, COC*H*HN), 4.47 (t, *J* = 9.2 Hz, 1H, pro-C$_{\alpha}$*H*), 4.02 (d, *J* = 17.9 Hz, 1H, *N*1ph-C*H*H), 3.90 (d, *J* = 17.7 Hz, 1H, *N*1ph-CH*H*), 3.87 (d, *J* = 15.3 Hz 1H, COCH*H*N), 3.75 (d, *J* = 18.8 Hz, 1H, COC*H*HN), 3.65 (dd, *J* = 17.6, 2.6 Hz, 1H, *N*1ay-C$_{1}$H*H*), 3.43 (t, *J* = 8.9 Hz, 1H, pro-C$_{\delta}$*H*H), 3.32–3.20 (m, 3H, pro-C$_{\delta}$H*H*, trp-C*H*H, COCH*H*N), 2.83 (dd, *J* = 14.6, 7.6 Hz, 1H, trp-CH*H*), 2.49 (s, 1H, *N*1ay-C*H*), 2.38–2.29 (m, 1H, pro-C$_{\beta}$*H*H), 2.09–2.03 (m, 1H, pro-C$_{\gamma}$*H*H), 1.90–1.83 (m, 2H, pro-C$_{\beta}$H*H*, pro-C$_{\gamma}$H*H*). – **^{13}C-NMR** (125 MHz, acetonitrile-*d*$_{3}$, ppm): δ = 173.3 (C$_{q}$, *C*ON), 170.6 (C$_{q}$, *C*ON), 170.2 (C$_{q}$, *C*ON), 167.0 (C$_{q}$, *C*ON), 138.3 (C$_{q}$, *C*$_{ar}$), 137.1 (C$_{q}$, *C*$_{ar}$), 129.6 (+, 2 × *N*1ph-CH), 128.9 (C$_{q}$, trp-*C*$_{ar}$), 128.7 (+, 2 × *N*1ph-CH), 128.4 (+, *N*1ph-CH), 124.9 (+, trp-*C*$_{ar}$H), 122.1 (+, trp*C*$_{ar}$H), 119.7 (+, trp-*C*$_{ar}$H), 119.5 (+, trp-*C*$_{ar}$H), 112.2 (+, trp-*C*$_{ar}$H), 111.9 (C$_{q}$, trp-*C*$_{ar}$), 80.0 (C$_{q}$, *N*1ay-CCH), 73.4 (+, *N*1ay-CH), 64.4 (+, pro-*C*$_{\alpha}$H), 53.0 (–, COCH$_{2}$N), 52.0 (–, COCH$_{2}$N), 51.5 (+, trp-*C*$_{\alpha}$H), 48.6 (–, *N*1ph-CH$_{2}$), 46.5 (–, pro-C$_{\delta}$H$_{2}$), 38.0 (–, *N*1ay-CH$_{2}$), 27.7 (–, trp-CH$_{2}$), 27.6 (–, pro-C$_{\beta}$H$_{2}$), 25.9 (–, pro-C$_{\gamma}$H$_{2}$). – **Analytical HPLC** (5–95% acetonitrile + 0.1% TFA in 30 min, detection at 218 nm): t$_{Ret}$ = 14.1 min (96%). – **MS** (m/z, MALDI-TOF): 505 [M+H]$^{+}$, 527 [M+Na]$^{+}$, 543 [M+K]$^{+}$.

***cyclo*-(*N*1ph-D-Pro-*N*1ay-L-Phe) (9d)**

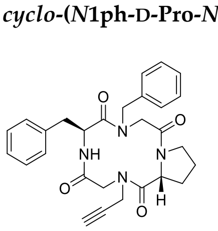

Fmoc-L-phenylalanine (310 mg, 800 µmol, 4.00 equiv.) was immobilized in accordance with **GP1**. The peptoid monomer *N*1ay was incorporated following **GP4** using propargylamine (102 µL, 88.1 mg, 1.60 mmol, 8.00 equiv.) as submonomer. Subsequently, Fmoc-D-proline (270 mg, 800 µmol, 4.00 equiv.) was coupled following **GP3**. The peptoid monomer *N*1ph was incorporated following **GP4** using benzylamine (158 µL, 117 mg, 1.60 mmol, 8.00 equiv.) as submonomer. Cleavage was performed according to **GP5**. The crude linear precursor (50.5 mg, 100 µmol, 1.00 equiv.) was

cyclized following **GP6** and deprotected in accordance with **GP7**. After purification *via* preparative reversed phase HPLC (5–95% acetonitrile in water with 0.1% TFA in 45 min), the macrocycle was isolated as colorless solid (8.20 mg, 16.9 µmol, 15% over 10 steps).

^{1}H-NMR (500 MHz, acetonitrile-d_3, ppm): δ = 7.38–7.14 (m, 10H, 5 × phe-C*H*, 5 × *N*1ph-C*H*), 6.81 (d, *J* = 10.2 Hz, 1H, phe-$N_{\alpha}H$), 5.47 (d, *J* = 15.4 Hz, 1H, COC*H*HN), 5.14 (q, *J* = 8.7 Hz, 1H, phe-$C_{\alpha}H$), 4.51–4.32 (m, 2H, pro-$C_{\alpha}H$, *N*1ay-C_1HH), 3.90–3.74 (m, 4H, COCH*H*N, COC*H*HN, *N*1ay-C_1HH, *N*1ph-C*H*H), 3.69 (d, *J* = 17.9 Hz, 1H, *N*1ph-CH*H*), 3.52 (d, *J* = 18.9 Hz, 1H, COCH*H*N), 3.36–3.25 (m, 2H, phe-C*H*H, pro-$C_{\delta}HH$), 3.22–3.13 (m, 1H, pro-$C_{\delta}HH$), 2.82 (dd, *J* = 13.6, 9.2 Hz, 1H, phe-CH*H*), 2.55 (s, 1H, *N*1ay-C*H*), 2.31–2.24 (m, 1H, pro-$C_{\beta}HH$), 2.06–2.00 (m, 1H, pro-$C_{\gamma}HH$), 1.87–1.79 (m, 2H, pro-$C_{\beta}HH$, pro-$C_{\gamma}HH$). – **^{13}C-NMR** (125 MHz, acetonitrile-d_3, ppm): δ = 173.0 (C_q, *C*ON), 170.9 (C_q, *C*ON), 170.4 (C_q, *C*ON), 166.7 (C_q, *C*ON), 139.5 (C_q, C_{ar}), 138.7 (C_q, C_{ar}), 130.7 (+, 2 × C_{ar}H), 129.4 (+, 2 × C_{ar}H), 128.9 (+, 2 × C_{ar}H), 128.6 (+, 2 × C_{ar}H), 128.1 (+, C_{ar}H), 127.1 (+, C_{ar}H), 80.1 (C_q, *N*1ay-CCH), 73.5 (+, *N*1ay-CCH), 64.0 (+, pro-C_{α}H), 52.6 (–,COCH_2N), 52.4 (–,COCH_2N), 52.0 (+, phe-C_{α}H), 49.6 (–, *N*1ph-CH_2), 46.5 (–, pro-$C_{\delta}H_2$), 39.6 (–, *N*1ay-C_1H_2), 38.5(–, phe-CH_2), 27.5 (–, pro-$C_{\beta}H_2$), 25.9 (–, pro-$C_{\gamma}H_2$). – **Analytical HPLC** (5–95% acetonitrile + 0.1% TFA in 10 min, detection at 218 nm): t_{Ret} = 6.208 min (>99%). – **MS** (m/z, MALDI-TOF): 487 $[M+H]^+$, 499 $[M+Na]^+$, 508 $[M+K]^+$.

***cyclo*-(*N*1py-D-Pro-*N*1ph-L-Phe) (9e)**

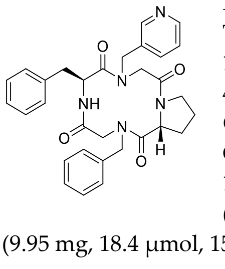

Fmoc-L-phenylalanine (310 mg, 800 µmol, 4.00 equiv.) was immobilized in accordance with **GP1**. The peptoid monomer *N*1ph was incorporated following **GP4** using benzylamine (158 µL, 117 mg, 1.60 mmol, 8.00 equiv.) as submonomer. Subsequently, Fmoc-D-proline (270 mg, 800 µmol, 4.00 equiv.) was coupled following **GP3**. The peptoid monomer *N*1py was incorporated following **GP4** using 3-pyridylmethylamine (168 µL, 173 mg, 1.60 mmol, 8.00 equiv.) as submonomer. Cleavage was performed according to **GP5**. The crude linear precursor (55.7 mg, 100 µmol, 1.00 equiv.) was cyclized following **GP6**. After purification *via* preparative reversed phase HPLC (5–95% acetonitrile in water with 0.1% TFA in 45 min), the macrocycle was isolated as colorless solid (9.95 mg, 18.4 µmol, 15% over 10 steps).

^{1}H-NMR (500 MHz, acetonitrile-d_3, ppm): δ = 8.77 (s, 1H, *N*1py-CC*H*N), 8.66 (d, *J* = 5.5 Hz, 1H, *N*1py-NC*H*), 8.41 (dt, *J* = 8.4, 1.6 Hz, 1H, *N*1py-NCHC*H*), 7.90 (dd, *J* = 8.1, 5.7 Hz, 1H, *N*1py-C*H*C), 7.39–7.15 (m, 10H, 5 × phe-C*H*, 5 × *N*1ph-C*H*), 6.66 (d, *J* = 10.3 Hz, 1H, phe-$N_{\alpha}H$), 5.19–5.05 (m, 3H, phe-$C_{\alpha}H$, 2 × COC*H*HN), 4.53–4.45 (m, 1H, pro-$C_{\alpha}H$), 4.36 (d, *J* = 16.1 Hz, 1H, COCH*H*N), 4.00 (d, *J* = 17.6 Hz, 1H, *N*1ph-C*H*H), 3.95 (d, *J* = 15.2 Hz, 1H, COCH*H*N), 3.84 (d, *J* = 18.0 Hz, 1H, *N*1ph-CH*H*), 3.69 (d, *J* = 18.8 Hz, 1H, *N*1py-C*H*H), 3.39–3.30 (m, 1H, pro-$C_{\delta}HH$), 3.23–3.13 (m, 3H, phe-C*H*H, pro-$C_{\delta}HH$, *N*1py-CH*H*), 2.70 (dd, *J* = 13.9, 9.9 Hz, 1H, phe-CH*H*), 2.39–2.30 (m, 1H, pro-$C_{\beta}HH$), 2.09–2.01 (m, 1H, pro-$C_{\gamma}HH$), 1.89–1.82 (m, 2H, pro-$C_{\beta}HH$, pro-$C_{\gamma}HH$). – **^{13}C-NMR** (125 MHz, acetonitrile-d_3, ppm): δ = 173.3 (C_q, *C*ON), 171.9 (C_q, *C*ON), 170.2 (C_q, *C*ON), 166.8 (C_q, *C*ON), 146.0 (+, *N*1py-CC_{ar}HN), 142.5 (+, *N*1py-NC_{ar}H), 141.6 (+, *N*1py-NCHC_{ar}H), 139.4 (C_q, C_{ar}), 139.1 (C_q, C_{ar}), 138.2 (C_q, C_{ar}), 130.7 (+, 2 × C_{ar}H), 129.8 (+, 2 × C_{ar}H), 128.9 (+, 2 × C_{ar}H), 128.8 (+, 2 × C_{ar}H), 128.6 (+, C_{ar}H), 127.4 (+, C_{ar}H), 127.2 (+, *N*1py-C_{ar}HC), 64.4 (+, pro-C_{α}H), 53.2 (–, COCH_2N), 52.1 (+, phe-C_{α}H), 51.6 (–, *N*1py-CH_2), 51.4 (–, *N*1ph-CH_2), 51.3 (–, COCH_2N), 46.4 (–, pro-$C_{\delta}H_2$), 38.1 (–, phe-CH_2), 27.7 (–, pro-$C_{\beta}H_2$), 26.0 (–, pro-$C_{\gamma}H_2$). – **Analytical HPLC** (5–95% acetonitrile + 0.1% TFA in 30 min, detection at 218 nm): t_{Ret} = 11.4 min (93%). – **MS** (m/z, MALDI-TOF): 540 $[M+H]^+$, 562 $[M+Na]^+$, 578 $[M+K]^+$.

***cyclo*-(*N*1py-D-Pro-*N*3m-L-Trp) (9f)**

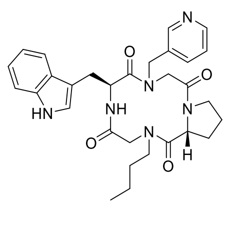

Fmoc-L-tryptophan (341 mg, 800 µmol, 4.00 equiv.) was immobilized in accordance with **GP1**. The peptoid monomer *N*3m was incorporated following **GP4** using *n*-butylamine (158 µL, 117 mg, 1.60 mmol, 8.00 equiv.) as submonomer. Subsequently, Fmoc-D-proline (270 mg, 800 µmol, 4.00 equiv.) was coupled following **GP3**. The peptoid monomer *N*1py was incorporated following **GP4** using 3-pyridylmethylamine (168 µL, 173 mg, 1.60 mmol, 8.00 equiv.) as submonomer. Cleavage was performed according to **GP5**. The crude linear precursor (56.2 mg, 100 µmol, 1.00 equiv.) was cyclized following **GP6**. After purification *via* preparative reversed phase HPLC (5–95% acetonitrile in water with 0.1% TFA in 45 min), the macrocycle was isolated as colorless solid (17.6 mg, 32.2 µmol, 28% over 10 steps).

^{1}H-NMR (500 MHz, acetonitrile-d_3, ppm): δ = 9.14 (s, 1H, tyr-N*H*), 8.73 (s, 1H, *N*1py-CC*H*N), 8.64 (d, *J* = 5.6 Hz, 1H, *N*1py-CHC*H*N), 8.35 (d, *J* = 8.1 Hz, 1H, *N*1py-CC*H*CH), 7.85 (dd, *J* = 8.1, 5.6 Hz, 1H, *N*1py-CHC*H*CH), 7.59 (d, *J* = 8.0 Hz, 1H, trp-C*H*), 7.35 (d, *J* = 8.1 Hz, 1H, trp-C*H*), 7.12–6.99 (m, 3H, 3 × trp-C*H*), 6.61 (d, *J* = 10.2 Hz, 1H, trp-$N_{\alpha}H$), 5.23–5.20 (m, 1H, trp-$C_{\alpha}H$), 5.18 (d, *J* = 15.9 Hz, 1H, COC*H*HN), 4.36–4.24 (m, 2H, pro-$C_{\alpha}H$, COCH*H*N), 4.00 (d, *J* = 18.0 Hz, 1H, *N*1py-C*H*H), 3.80 (d, *J* = 18.0 Hz, 1H, *N*1py-CH*H*), 3.71–3.61 (m, 2H, COC*H*HN, *N*3m-C_1HH), 3.38–3.29 (m, 3H, pro-$C_{\delta}HH$, trp-C*H*H, COCH*H*N), 3.24–3.14 (m, 1H, pro-$C_{\delta}HH$), 3.04 (dd, *J* = 14.6, 7.8 Hz, 1H, trp-CH*H*), 2.40–2.32 (m, 1H, *N*3m-C_1HH), 2.28–2.21 (m, 1H, pro-$C_{\beta}HH$), 2.06–2.01 (m, 1H, pro-$C_{\gamma}HH$), 1.87–1.77 (m, 2H, pro-$C_{\beta}HH$, pro-$C_{\gamma}HH$), 1.34–1.29 (m, 2H, *N*3m-C_2H_2), 1.25–1.15 (m, 2H, *N*3m-C_3H_2), 0.88 (t, *J* = 7.3 Hz, 3H, *N*3m-CH_3). – **^{13}C-NMR** (125 MHz, acetonitrile-d_3, ppm): δ = 172.7 (C_q, *C*ON), 172.2 (C_q, *C*ON), 170.1 (C_q, *C*ON), 167.4 (C_q, *C*ON), 145.5 (+, *N*1py-C_{ar}H), 142.7 (+, *N*1py-C_{ar}H), 141.8 (+, *N*1py-C_{ar}H), 139.2 (C_q, *N*1py-C_{ar}), 137.1 (C_q, trp-C_{ar}), 129.0 (C_q, trp-C_{ar}), 127.3 (+, *N*1py-C_{ar}H), 124.8 (+, trp-C_{ar}H), 122.2 (+, trp-C_{ar}H), 119.6 (+, trp-C_{ar}H), 119.4 (+, trp-C_{ar}H), 112.2 (+, trp-C_{ar}H), 111.9 (C_q, trp-C_{ar}), 64.5 (+, pro-C_{α}H), 51.8 (+, trp-C_{α}H), 51.6 (–,

COCH$_2$N), 51.3 (–, COCH$_2$N), 51.2 (–, *N*1py-CH$_2$), 50.0 (–, *N*3m-C$_1$H$_2$), 46.4 (–, pro-C$_\delta$H$_2$), 29.4 (–, *N*3m-C$_2$H$_2$), 27.6 (–, trp-CH$_2$), 27.5 (–, pro-C$_\beta$H$_2$), 25.9 (–, pro-C$_\gamma$H$_2$), 20.7 (–, *N*3m-C$_3$H$_2$), 14.2 (+, *N*3m-CH$_3$). – **Analytical HPLC** (5–95% acetonitrile + 0.1% TFA in 30 min, detection at 218 nm): t$_{Ret}$ = 10.8 min (97%). – **MS** (m/z, MALDI-TOF): 545 [M+H]$^+$, 567 [M+Na]$^+$, 583 [M+K]$^+$.

***cyclo*-(*N*3m-D-Pro-*N*1al-L-Trp) (9g)**

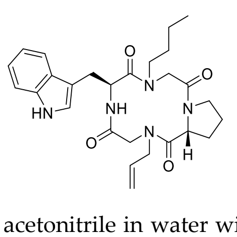

Fmoc-L-tryptophan (341 mg, 800 µmol, 4.00 equiv.) was immobilized in accordance with **GP1**. The peptoid monomer *N*1al was incorporated following **GP4** using allylamine (120 µL, 91.4 mg, 1.60 mmol, 8.00 equiv.) as submonomer. Subsequently, Fmoc-D-proline (270 mg, 800 µmol, 4.00 equiv.) was coupled following **GP3**. The peptoid monomer *N*3m was incorporated following **GP4** using *n*-butylamine (158 µL, 117 mg, 1.60 mmol, 8.00 equiv.) as submonomer. Cleavage was performed according to **GP5**. The crude linear precursor (51.2 mg, 100 µmol, 1.00 equiv.) was cyclized following **GP6**. After purification *via* preparative reversed phase HPLC (5–95% acetonitrile in water with 0.1% TFA in 45 min), the macrocycle was isolated as colorless solid (22.0 mg, 44.6 µmol, 34% over 10 steps).

^{1}H-NMR (500 MHz, acetonitrile-*d*$_3$, ppm): δ = 9.08 (s, 1H,trp-N*H*), 7.61 (d, *J* = 7.9 Hz, 1H, trp-C$_{ar}$*H*), 7.35 (d, *J* = 8.2 Hz, 1H, trp-C$_{ar}$*H*), 7.12–7.05 (m, 2H, 2 × trp-C$_{ar}$*H*), 7.04–6.99 (m, 1H, trp-C$_{ar}$*H*), 6.56 (d, *J* = 10.1 Hz, 1H, trp-N$_\alpha$*H*), 5.67–5.55 (m, 1H, *N*1al-C*H*), 5.10–5.01 (m, 2H, *N*1al-C$_3$*H*H, trp-C$_\alpha$*H*), 4.95 (d, *J* = 17.3 Hz, 1H, *N*1al-C$_3$H*H*), 4.37 (t, *J* = 9.3 Hz, 1H, pro-C$_\alpha$*H*), 4.24 (d, *J* = 15.5 Hz, 1H, *N*1al-C$_1$*H*H), 4.06–3.97 (m, 1H, *N*3me-C$_1$*H*H), 3.84 (d, *J* = 17.9 Hz, 1H, COC*H*HN), 3.76 (d, *J* = 17.9 Hz, 1H, COCH*H*N), 3.68 (d, *J* = 18.7 Hz, 1H, COC*H*HN), 3.43–3.37 (m, 1H, pro-C$_\delta$*H*H), 3.31 (dd, *J* = 14.5, 6.2 Hz, 1H, trp-C$_\beta$*H*H), 3.27–3.17 (m, 2H, pro-C$_\delta$H*H*, COCH*H*N), 3.04–2.93 (m, 2H, trp-C$_\beta$H*H*, *N*1al-C$_1$H*H*), 2.79–2.73 (m, 1H, *N*3me-C$_1$H*H*), 2.32–2.22 (m, 1H, pro-C$_\beta$*H*H), 2.10–2.01 (m, 1H, pro-C$_\gamma$*H*H), 1.90–1.79 (m, 2H, pro-C$_\beta$H*H*, pro-C$_\gamma$H*H*), 1.50–1.40 (m, 2H, *N*3me-C$_2$*H*$_2$), 1.31–1.24 (m, 2H, *N*3me-C$_3$*H*$_2$), 0.90 (t, *J* = 7.4 Hz, 3H, *N*3me-C*H*$_3$). – **^{13}C-NMR** (125 MHz, acetonitrile-*d*$_3$, ppm): δ = 173.0 (C$_q$, *C*ON), 170.6 (C$_q$, *C*ON), 170.5 (C$_q$, *C*ON), 167.0 (C$_q$, *C*ON), 137.1 (C$_q$, trp-*C*$_{ar}$), 133.4 (+, *N*1al-CH), 129.2 (C$_q$, trp-*C*$_{ar}$), 124.9 (+, trp-*C*$_{ar}$H), 122.1 (+, trp-*C*$_{ar}$H), 119.6 (+, trp-*C*$_{ar}$H), 119.4 (+, trp-*C*$_{ar}$H), 117.9 (–, *N*1al-C$_3$H$_2$), 112.2 (C$_q$, trp-*C*$_{ar}$), 112.1 (+, trp-*C*$_{ar}$H), 64.3 (+, pro-C$_\alpha$H), 52.2 (–, *N*1al-C$_1$H$_2$), 51.9 (+, trp-C$_\alpha$H), 51.3 (–, COCH$_2$N), 50.0 (–, COCH$_2$N), 49.5 (–, *N*3me-C$_1$H$_2$), 46.4 (–, pro-C$_\gamma$H$_2$), 30.3 (–, *N*3me-C$_2$H$_2$), 27.7 (–, pro-C$_\beta$H$_2$), 27.6 (–, trp-C$_\beta$H$_2$), 26.0 (–, pro-C$_\gamma$H$_2$), 20.8 (–, *N*3me-C$_3$H$_2$), 14.2 (+, *N*3me-CH$_3$). – **Analytical HPLC** (5–95% acetonitrile + 0.1% TFA in 30 min, detection at 218 nm): t$_{Ret}$ = 12.3 min (93%). – **MS** (m/z, MALDI-TOF): 494 [M+H]$^+$, 516 [M+Na]$^+$, 532 [M+K]$^+$.

***cyclo*-(*N*3m-D-Pro-*N*1ay-L-Trp) (9h)**

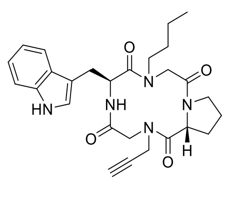

Fmoc-L-tryptophan (341 mg, 800 µmol, 4.00 equiv.) was immobilized in accordance with **GP1**. The peptoid monomer *N*1ay was incorporated following **GP4** using propargylamine (102 µL, 88.1 mg, 1.60 mmol, 8.00 equiv.) as submonomer. Subsequently, Fmoc-D-proline (270 mg, 800 µmol, 4.00 equiv.) was coupled following **GP3**. The peptoid monomer *N*3m was incorporated following **GP4** using *n*-butylamine (158 µL, 117 mg, 1.60 mmol, 8.00 equiv.) as submonomer. Cleavage was performed according to **GP5**. The crude linear precursor (51.1 mg, 100 µmol, 1.00 equiv.) was cyclized following **GP6**. After purification *via* preparative reversed phase HPLC (5–95% acetonitrile in water with 0.1% TFA in 45 min), the macrocycle was isolated as colorless solid (10.6 mg, 21.5 µmol, 20% over 10 steps).

^{1}H-NMR (500 MHz, acetonitrile-*d*$_3$, ppm): δ = 9.05 (s, 1H, trp-N*H*), 7.63 (d, *J* = 7.8 Hz, 1H, trp-C*H*), 7.35 (dt, *J* = 8.1, 1.0 Hz, 1H, trp-C*H*), 7.12–7.06 (m, 2H, 2 × trp-C*H*), 7.03 (ddd, *J* = 7.9, 6.9, 1.0 Hz, 1H, trp-C*H*), 6.66 (d, *J* = 10.1 Hz, 1H, trp-N$_\alpha$*H*), 5.01 (q, *J* = 7.7 Hz, 1H, trp-C$_\alpha$*H*), 4.44–4.36 (m, 1H, pro-C$_\alpha$*H*), 4.21 (d, *J* = 17.4 Hz, 1H, *N*1ay-C$_1$*H*H), 4.05–3.96 (m, 1H, *N*3m-C$_1$*H*H), 3.87–3.70 (m, 3H, COC*H*HN, COC*H*$_2$N), 3.60 (d, *J* = 17.5 Hz, 1H, *N*1ay-C$_1$*H*H), 3.51 (d, *J* = 18.8 Hz, 1H, COCH*H*N), 3.43–3.37 (m, 1H, pro-C$_\delta$*H*H), 3.34 (dd, *J* = 14.6, 6.4 Hz, 1H, trp-C*H*H), 3.23–3.21 (m, 1H, pro-C$_\delta$H*H*), 2.93 (dd, *J* = 14.5, 7.2 Hz, 1H, trp-CH*H*), 2.87–2.69 (m, 1H, *N*3m-C$_1$H*H*), 2.46 (s, 1H, *N*1ay-C*H*), 2.31–2.22 (m, 1H, pro-C$_\beta$*H*H), 2.12–2.02 (m, 1H, pro-C$_\gamma$*H*H), 1.91–1.76 (m, 2H, pro-C$_\beta$H*H*, pro-C$_\gamma$H*H*), 1.49–1.39 (m, 2H, *N*3m-C$_2$*H*$_2$), 1.35–1.20 (m, 2H, *N*3m-C$_3$*H*$_2$), 0.89 (t, *J* = 7.4 Hz, 3H, *N*3m-C*H*$_3$). – **^{13}C-NMR** (125 MHz, acetonitrile-*d*$_3$, ppm): δ = 173.40(C$_q$, *C*ON), 170.7 (C$_q$, *C*ON), 170.4 (C$_q$, *C*ON), 166.8 (C$_q$, *C*ON), 137.1 (C$_q$, trp-*C*$_{ar}$), 129.0 (C$_q$, trp-*C*$_{ar}$), 124.8 (+, trp-*C*$_{ar}$H), 122.1 (+, trp*C*$_{ar}$H), 119.6 (+, trp-*C*$_{ar}$H), 119.4 (+, trp-*C*$_{ar}$H), 112.3 (C$_q$, trp-*C*$_{ar}$), 112.2 (+, trp-*C*$_{ar}$H), 80.1 (C$_q$, *N*1ay-CCH), 73.3 (+, *N*1ay-CH), 64.1 (+, pro-C$_\alpha$H), 52.1 (–, COCH$_2$N), 52.0 (+, trp-C$_\alpha$H), 50.0 (–, COCH$_2$N), 49.4 (–, *N*3m-CH$_2$), 46.4 (–, pro-C$_\delta$H$_2$), 39.3 (–, *N*1ay-CH$_2$), 30.2 (–, *N*3m-C$_2$H$_2$), 27.9 (–, trp-CH$_2$), 27.5 (–, pro-C$_\beta$H$_2$), 25.9 (–, pro-C$_\gamma$H$_2$), 20.7 (–, *N*3m-C$_3$H$_2$), 14.2 (+, *N*3m-CH$_3$). – **Analytical HPLC** (5–95% acetonitrile + 0.1% TFA in 30 min, detection at 218 nm): t$_{Ret}$ = 13.4 min (>99%). – **MS** (m/z, MALDI-TOF): 492 [M+H]$^+$, 514 [M+Na]$^+$, 530 [M+K]$^+$.

***cyclo*-(*N*3m-D-Pro-*N*1ph-L-Phe) (9i)**

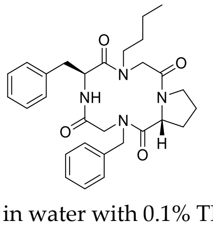

Fmoc-L-phenylalanine (310 mg, 800 µmol, 4.00 equiv.) was immobilized in accordance with **GP1**. The peptoid monomer *N*1ph was incorporated following **GP4** using benzylamine (174 µL, 171 mg, 1.60 mmol, 8.00 equiv.) as submonomer. Subsequently, Fmoc-D-proline (270 mg, 800 µmol, 4.00 equiv.) was coupled following **GP3**. The peptoid monomer *N*3m was incorporated following **GP4** using *n*-butylamine (158 µL, 117 mg, 1.60 mmol, 8.00 equiv.) as submonomer. Cleavage was performed according to **GP5**. The crude linear precursor (52.3 mg, 100 µmol, 1.00 equiv.) was cyclized following **GP6**. After purification *via* preparative reversed phase HPLC (5–95% acetonitrile in water with 0.1% TFA in 45 min), the macrocycle was isolated as colorless solid (12.1 mg, 24.0 µmol, 12% over 10 steps).

^{1}H-NMR (500 MHz, acetonitrile-d_3, ppm): δ = 7.41–7.13 (m, 10H, 5 × phe-C*H*, 5 × *N*1ph-C*H*), 6.57 (d, *J* = 10.3 Hz, 1H, phe-N$_\alpha$*H*), 5.03 (d, *J* = 15.2 Hz, 1H, COC*H*HN), 4.97 (td, *J* = 9.7, 5.6 Hz, 1H, phe-C$_\alpha$*H*), 4.49 (t, *J* = 9.3 Hz, 1H, pro-C$_\alpha$*H*), 4.03–3.97 (m, 1H, *N*3m-C$_1$*H*H), 3.94 (d, *J* = 15.3 Hz, 1H, COCH*H*N), 3.87–3.74 (m, 2H, *N*1ph-C*H*$_2$), 3.70 (d, *J* = 18.7 Hz, 1H, COC*H*HN), 3.41 (t, *J* = 9.0 Hz, 1H, pro-C$_\delta$*H*H), 3.25–3.10 (m, 3H, phe-C*H*H, pro-C$_\delta$H*H*, COCH*H*N), 2.82–2.72 (m, 1H, *N*3m-C$_1$H*H*), 2.67 (dd, *J* = 13.8, 9.2 Hz, 1H, phe-CH*H*), 2.38–2.30 (m, 1H, pro-C$_\beta$*H*H), 2.10–2.01 (m, 1H, pro-C$_\gamma$*H*H), 1.91–1.81 (m, 2H, pro-C$_\beta$H*H*, pro-C$_\gamma$H*H*), 1.46 (dq, *J* = 13.8, 7.8 Hz, 2H, *N*3m-C$_2$*H*$_2$), 1.32–1.23 (m, 2H, *N*3m-C$_3$*H*$_2$), 0.90 (t, *J* = 7.3 Hz, 3H, *N*3m-C*H*$_3$). – **^{13}C-NMR** (125 MHz, acetonitrile-d_3, ppm): δ = 173.4 (C$_q$, *C*ON), 170.7 (C$_q$, *C*ON), 170.3 (C$_q$, *C*ON), 166.6 (C$_q$, *C*ON), 139.6 (C$_q$, *C*$_{ar}$), 138.3 (C$_q$, *C*$_{ar}$), 130.7 (+, 2 × *C*$_{ar}$H), 129.7 (+, 2 × *C*$_{ar}$H), 128.8 (+, 3 × *C*$_{ar}$H), 128.5 (+, *C*$_{ar}$H), 127.0 (+, 2 × *C*$_{ar}$H), 64.4 (+, pro-*C*$_\alpha$H), 53.1 (–, CO*C*H$_2$N), 52.3 (+, phe-*C*$_\alpha$H), 51.6 (–, CO*C*H$_2$N), 50.0 (–, *N*1ph-*C*H$_2$), 49.5 (–, *N*3m-*C*$_1$H$_2$), 46.4 (–, pro-*C*$_\delta$H$_2$), 38.4 (–, phe-*C*H$_2$), 30.3 (–, *N*3m-*C*$_2$H$_2$), 27.7 (–, pro-*C*$_\beta$H$_2$), 26.0 (–, pro-*C*$_\gamma$H$_2$), 20.7 (–, *N*3m-*C*$_3$H$_2$), 14.2 (–, *N*3m-*C*H$_3$). – **Analytical HPLC** (5–95% acetonitrile + 0.1% TFA in 10 min, detection at 218 nm): t_{Ret} = 6.429 min (>99%). – **MS** (m/z, MALDI-TOF): 505 [M+H]$^+$, 527 [M+Na]$^+$, 543 [M+K]$^+$.

.

Structure elucidation of 9a *via* NMR spectroscopy

Determination of internuclear distances from NOESY spectra

Integration of NOESY cross-peaks was performed with TopSpin (version 3.5 pl 7). Internuclear distances r were calculated according to equation 1 using cross-peak intensities I from a NOESY spectrum with a mixing time of 200 ms. The reference values I_{ref} and r_{ref} were acquired by integration of an NOE cross-peak belonging to a pair of protons with a known internuclear distance. Here, a pair of geminal protons in the cyclohexane moiety was used as a reference and r_{ref} was chosen to be 1.76 Å.

$$\frac{I}{I_{ref}} = \frac{r^{-6}}{r_{ref}^{-6}} \tag{1}$$

Molecular modelling

The molecular modelling software Avogadro (Version 1.2.0, using OpenBabel Version 2.3.90)[1),] [11] was used to construct a three-dimensional model of **9a**. Distance and angle constraints were incorporated using the molecular mechanics extension and manipulation tool. Energy minimization was carried out using the MMFF94 force field [12] and steepest descent algorithm.

DFT calculations

DFT calculations were carried out in Turbomole (version 7.5.0)[2)] using the B3-LYP functional [13-15] and the def2-TZVP basis set [16, 17]. A gridsize of 5 was used [18]. Solvation effects were considered through the use of the COSMO module [19], with a dielectric constant ε of 35.95 for acetonitrile [20].

Preparation of the anisotropic sample of 9a

The measurement of RDCs requires the preparation of an anisotropic sample in which the molecule of interest is weakly aligned. In order to prepare such a sample, a covalently cross-linked polyethylene glycol (PEG) gel stick was swollen in the peptide-peptoid solution and uniaxially stretched using a stretching apparatus.

The PEG gel was prepared *via* radical polymerization. The synthesis and casting of the PEG gel was carried out according to the technique described by T. Gloge [21]. 260 mg of polyethylene glycol diacrylate (PEG-DA) monomer (m = 35 kDa) was added to 936 µl of distilled water and 52 µl of an aqueous 1% (v/v) tetramethylethylenediamine (TEMED) solution. The solution was mixed and degassed using a Schlenk line. 52 µl of an aqueous 1% (w/w) ammonium persulfate (APS) solution was added to initiate radical polymerization. Immediately after initiation, the mixture was drawn into a casting apparatus. The apparatus was sealed and placed into a water bath at 40 °C for approximately 24 h. The polymerized gel stick was washed by placing it into deionized water for 1-2 days to swell. The gel was then cut into small pieces and dried in a drying oven at 40 °C for approximately 3 days.

One piece of the dried PEG gel (l = 1 cm, m = 21.2 mg) was then placed into 400 µl of a 45 mM solution of **9a** in deuterated acetonitrile for approximately 2 h to swell. The swollen gel (l = 2.1 cm) was transferred into a KalRez® 8002UP perfluoroelastomer tube with an inner diameter of 3.2 mm. The KalRez® tube with the swollen gel was then transferred into an NMR tube which was open at both ends and manually stretched using a stretching apparatus. The final length of the stretched swollen gel was 7.6 cm.

Extraction and evaluation of RDCs

The residual dipolar couplings (RDCs) were obtained by comparing CLIP-HSQC [1] and P.E.HSQC [2] spectra of **9a** recorded in an isotropic environment (yielding the scalar coupling constants ^1J(CH) and ^2J(HH)) and in an anisotropic environment (yielding the total coupling values ^1T(CH) and ^2T(HH)). These couplings and their errors were extracted by alignment of the respective traces. The residual dipolar couplings ^1D(CH) and ^2D(HH) were then calculated according to equations (2) and (3) and the errors of the dipolar couplings according to Gaussian error propagation (equation (4)). The experimentally determined RDCs and experimental errors are shown in **table S3**.

$$^1D(CH) = {}^1T(CH) - {}^1J(CH) \tag{2}$$

$$^2D(HH) = {}^2T(HH) - {}^2J(HH) \tag{3}$$

$$\Delta D = \sqrt{(\Delta T)^2 + (\Delta J)^2} \tag{4}$$

The agreement between the RDCs and the constructed 3D model for **9a** was tested using the MSpin-RDC[22] software. The experimental RDCs and their standard deviations $\sigma(D)$ were needed as input. $\sigma(D)$ was therefore estimated by dividing the experimental error $|\Delta D|$ by 3 (under the assumption that $|\Delta D| \approx 3\sigma$). The resolution of the processed spectrum from which the RDC was extracted was taken as a minimum physically sound $\sigma(D)$ value: $\sigma(D)_{min} = 0.2$ Hz. An alignment tensor was then fit from experimental data using single value decomposition (SVD).

[1)] Avogadro: an open-source molecular builder and visualization tool. Version 1.2.0. http://avogadro.cc/.
[2)] TURBOMOLE V7.5.0, a development of University of Karlsruhe and Forschungszentrum Karlsruhe GmbH, 1989-2007, TURBOMOLE GmbH, since 2007; available from http://www.turbomole.com.

In order to account for fast rotational movement, averaging was used for methyl- and phenyl groups. Monte-Carlo bootstrapping was used for the calculation of errors. Error calculation was performed with a uniform distribution and a distribution size of 512. Using the calculated alignment tensor, RDCs were then back-calculated.

SVDs with different input data were carried out. For SVD 1, all experimentally determined RDCs were used as input for the fitting of the alignment tensor. However, as the method does not account for conformational mobility, it is probable that the averaging of RDCs in the more mobile sidechains cannot be properly represented. Therefore, a second SVD (SVD 2) was carried out, in which only the RDCs of the more rigid 12-membered backbone were used as input. The back-calculated RDC values for both SVDs can be found in **table S3**.

Internuclear distances for 9a

Table S2: Internuclear distances calculated from NOESY spectra of peptide-peptoid hybrid **9a**. Relevant parts of the molecule are labelled in the figure of **9a** below (N1 – green, proline (Pro) – orange, N2 – purple, phenylalanine (Phe) – black).

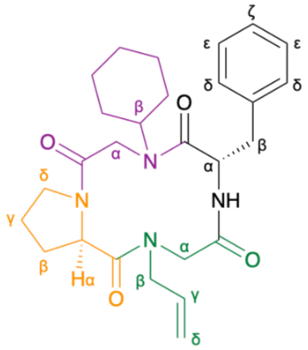

**atom 1**	**atom 2**	**r / Å**
Phe NH	Phe H_{α}	3.25
Phe NH	Phe $H_{\beta 1}$	2.96
Phe NH	N1 $H_{\beta 1}$	2.74
Phe NH	N1 $H_{\beta 2}$	3.68
Phe NH	N1 $H_{\alpha 1}$	2.88
Phe H_{α}	Phe $H_{\beta 1}$	2.93
Phe H_{α}	Phe $H_{\beta 2}$	2.50
Phe H_{α}	N2 $H_{\alpha 1}$	2.15
N1 $H_{\alpha 2}$	Pro $H_{\beta 1}$	2.17
N1 $H_{\alpha 2}$	Pro $H_{\gamma 1}$	3.97
Pro $H_{\delta 2}$	N2 $H_{\alpha 2}$	2.33
N2 $H_{\alpha 2}$	N2 H_{β}	3.45

RDCs for 9a

Table S3: Experimentally determined RDCs for peptide-peptoid hybrid **9a** *D*, the respective experimental errors |Δ*D*|, and standard deviations σ(*D*). The back-calculated RDCs D_{cal} and their standard deviations σ(D_{cal}) are given for SVDs 1 and 2. For SVD 1, 14 out of 26 RDCs were reproduced within the experimental error. For SVD 2, 7 out of 8 RDCs were reproduced within the experimental error. Relevant parts of the molecule are labelled in the figure of 9a below (N1 – green, proline (Pro) – orange, N2 – purple, phenylalanine (Phe) – black).

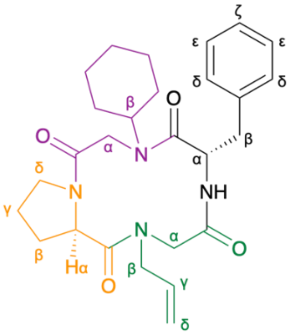

atoms	*D* / Hz	|Δ *D*| / Hz	σ (*D*) / Hz	D_{cal}(1) / Hz	σ (D_{cal}(1)) / Hz	D_{cal}(2) / Hz	σ (D_{cal}(2)) / Hz
Pro C_{α}-H_{α}	-7.8	0.4	0.2	-7.6	0.3	-7.6	0.3
Pro C_{β}-$H_{\beta 1}$	-16.6	3.2	1.1	-12.1	0.3	-	-
Pro C_{β}-$H_{\beta 2}$	-1.1	1.1	0.4	-0.8	0.3	-	-
Pro $H_{\beta 1}$-$H_{\beta 2}$	-2.5	9.0	3.0	-6.0	0.5	-	-
Pro $H_{\gamma 1}$-$H_{\gamma 2}$	-2.5	5.5	1.8	-6.4	0.3	-	-
Pro C_{δ}-$H_{\delta 1}$	-8.9	2.3	0.8	-8.0	0.4	-	-
Pro C_{δ}-$H_{\delta 2}$	-8.3	1.3	0.5	-7.8	0.3	-	-
Pro $H_{\delta 1}$-$H_{\delta 2}$	-9.8	0.8	0.3	-11.5	0.2	-	-
N2 C_{α}-$H_{\alpha 1}$	-5.1	1.1	0.4	-5.0	0.3	-4.2	0.3
N2 C_{α}-$H_{\alpha 2}$	-0.3	0.5	0.2	-1.0	0.3	0.3	0.3
N2 $H_{\alpha 1}$-$H_{\alpha 2}$	-8.8	3.9	1.3	-10.9	0.3	-10.3	0.4
N2 C_{β}-H_{β}	12.6	0.9	0.3	12.0	0.3	-	-
Phe C_{α}-H_{α}	10.3	0.8	0.3	8.7	0.2	9.7	0.3
Phe C_{β}-$H_{\beta 1}$	6.2	0.8	0.3	8.6	0.2	-	-
Phe C_{β}-$H_{\beta 2}$	1.1	0.7	0.2	-3.0	0.3	-	-
Phe $H_{\beta 1}$-$H_{\beta 2}$	1.4	1.3	0.4	0.4	0.2	-	-
Phe C_{δ}-H_{δ}	-1.3	0.2	0.2	-0.9	0.1	-	-
Phe C_{ε}-H_{ε}	-2.2	0.3	0.2	-1.0	0.1	-	-
Phe C_{ζ}-H_{ζ}	-14.0	0.8	0.3	-11.5	0.3	-	-
N1 C_{α}-$H_{\alpha 1}$	-11.3	1.1	0.4	-12.1	0.2	-11.1	0.4
N1 C_{α}-$H_{\alpha 2}$	14.9	0.3	0.2	12.9	0.2	14.7	0.2
N1 $H_{\alpha 1}$-$H_{\alpha 2}$	0.5	2.5	0.8	-0.1	0.3	0.9	0.4
N1 C_{β}-$H_{\beta 1}$	-2.9	1.0	0.3	1.1	0.3	-	-
N1 C_{β}-$H_{\beta 2}$	1.7	0.8	0.3	1.3	0.3	-	-
N1 $H_{\beta 1}$-$H_{\beta 2}$	-2.9	1.2	0.4	-3.6	0.4	-	-
N1 C_{γ}-H_{γ}	2.0	0.2	0.2	1.6	0.3	-	-
N1 C_{δ}-$H_{\delta 1}$	1.5	0.2	0.2	1.5	0.3	-	-
N1 C_{δ}-$H_{\delta 2}$	-7.8	0.3	0.2	-7.5	0.4	-	-

Coordinate file (.xyz) for the 3D model of 9a

71

Energy =

O	-2.6990258	-1.2814759	-1.8846097
C	-1.7692669	-1.1108790	-1.0978477
N	-0.7137020	-1.9676710	-1.0341495
C	-0.7444683	-3.1994527	-1.8625650
C	0.4755852	-3.3556958	-2.7773783
C	0.3509680	-4.6254435	-3.6292123
C	0.1456720	-5.8729554	-2.7657790
C	-1.0613395	-5.7107160	-1.8380452
C	-0.9521434	-4.4421627	-0.9836755
C	0.4555370	-1.7264832	-0.2114288
C	1.4185072	-0.7083714	-0.8408530
N	2.3961630	-0.2307550	-0.0425306
C	2.8458294	-0.7861246	1.2489004
C	4.3103687	-0.3470206	1.3193051
C	4.3254915	0.9995539	0.5840683
C	3.3126662	0.7930549	-0.5611001
H	3.8253533	0.3990332	-1.4397964
C	2.6665083	2.0862842	-1.0857727
O	3.1717885	2.6059062	-2.0773168
O	1.2692972	-0.2962100	-1.9884152
C	-1.8726534	0.0582138	-0.0720982
H	-1.8621796	-0.3907514	0.9230842
C	-3.2092762	0.8049681	-0.2619138
C	-3.4855545	1.8368687	0.8052474
C	-3.2794550	3.1962917	0.5640743
C	-3.5383117	4.1470889	1.5485867
C	-4.0123791	3.7498929	2.7944192
C	-4.2286380	2.3968294	3.0462895
C	-3.9680743	1.4526279	2.0597764
N	-0.7416927	0.9775139	-0.0991133
C	-0.0478409	1.3147522	1.0062886
C	1.1125088	2.3026276	0.8638876
N	1.5820231	2.6459000	-0.4702430
C	1.0224345	3.8823739	-1.0553346
C	1.6639801	5.1239996	-0.5049278
C	1.0027800	6.0884718	0.1249933
O	-0.2906590	0.8677001	2.1237034
H	-1.6219262	-3.0853027	-2.4954564
H	0.5730618	-2.4744280	-3.4124509
H	1.3922037	-3.4212716	-2.1813512
H	-0.4970096	-4.5174439	-4.3154791
H	1.2435809	-4.7354532	-4.2507828
H	0.0170618	-6.7541784	-3.4004032
H	1.0448865	-6.0475751	-2.1630325
H	-1.9754795	-5.6601161	-2.4407854
H	-1.1635418	-6.5834027	-1.1874937
H	-0.1139066	-4.5513842	-0.2864509
H	-1.8541309	-4.3113738	-0.3800276
H	0.1738581	-1.3927804	0.7879075
H	0.9812976	-2.6702425	-0.0676189

H	2.2610737	-0.3684791	2.0733756
H	2.7332317	-1.8686667	1.2656457
H	4.6654036	-0.2701370	2.3460637
H	4.9384265	-1.0686128	0.7932036
H	4.0102857	1.8027947	1.2522952
H	5.3065717	1.2633867	0.1939850
H	-3.2103276	1.2745790	-1.2470137
H	-4.0042521	0.0598039	-0.2677637
H	-2.9231893	3.5166671	-0.4085830
H	-3.3755533	5.1976663	1.3390720
H	-4.2179924	4.4872249	3.5610242
H	-4.6052404	2.0780312	4.0110478
H	-4.1459280	0.4025579	2.2654861
H	-0.4406384	1.3493620	-0.9876875
H	0.7956826	3.2242138	1.3584708
H	1.9155251	1.9104925	1.4803629
H	1.1689841	3.8260428	-2.1328185
H	-0.0511359	3.8920636	-0.8591111
H	2.7367253	5.2064079	-0.6540050
H	1.5066473	6.9740528	0.4932351
H	-0.0690574	6.0327162	0.2856221

HPLC traces

Table S4: Analytical HPLC traces of macrocycles **7a-j**, **8a-j** and **9a-j**.

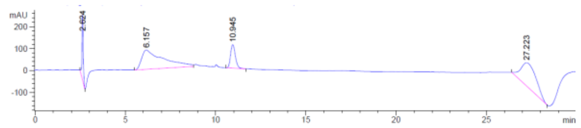

7a

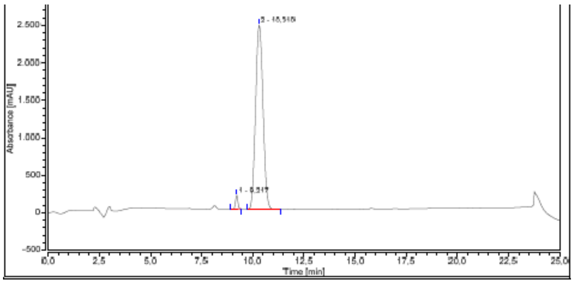

7b

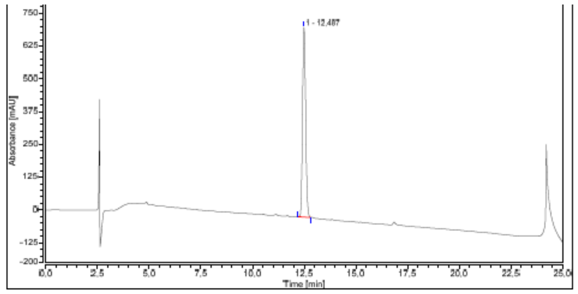

7c

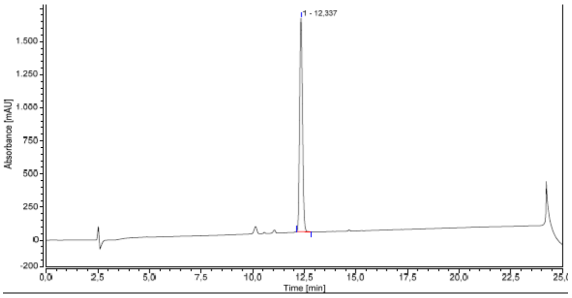

7d

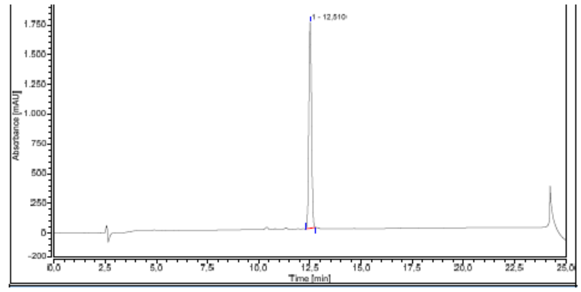

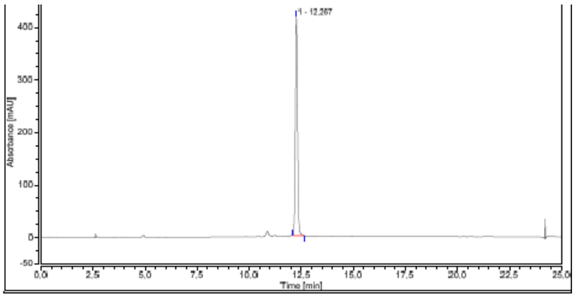

7e	7f

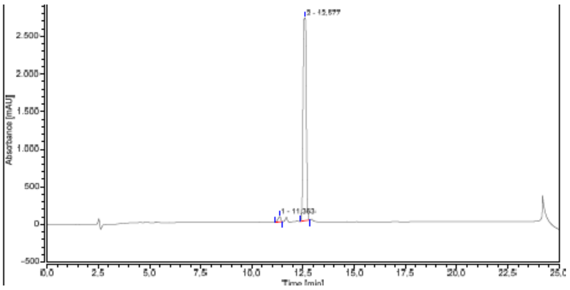

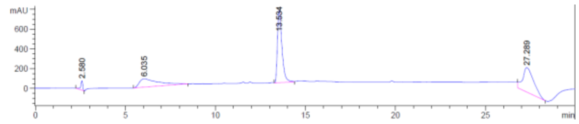

7g

7h

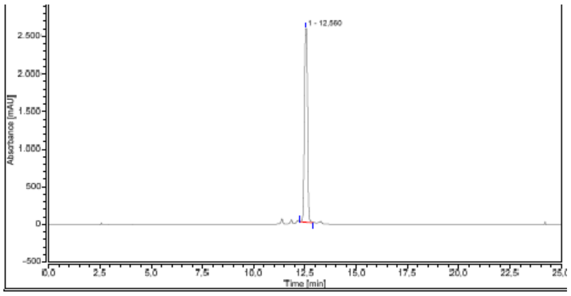

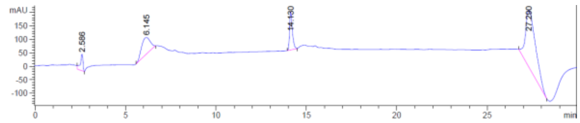

7i

8a

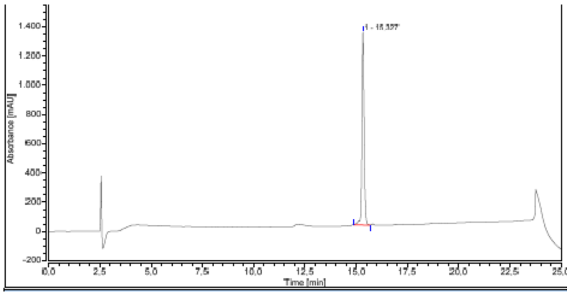

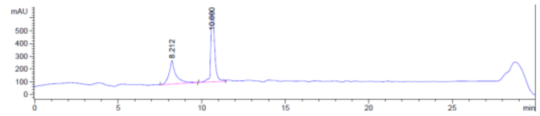

8b

8c

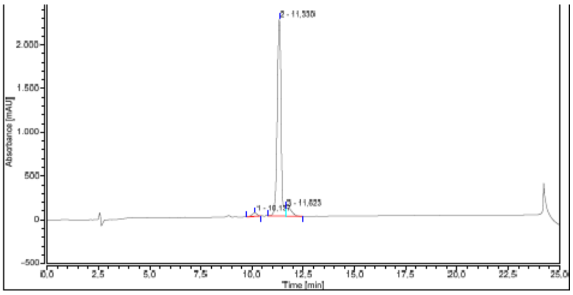

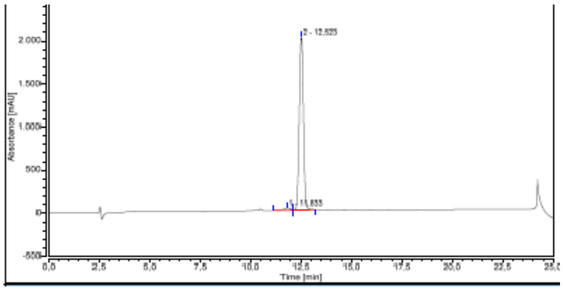

8d

8e

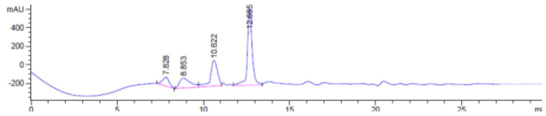

8f

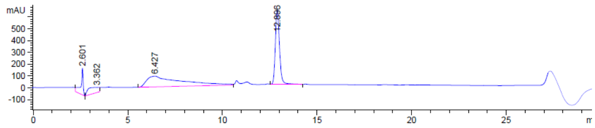

8g

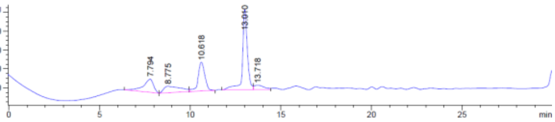

8h

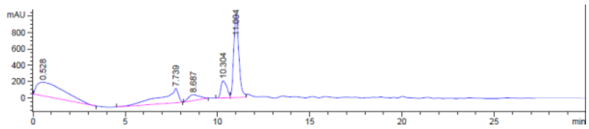

8i

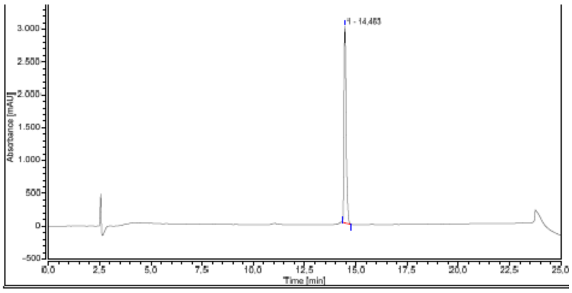

9a

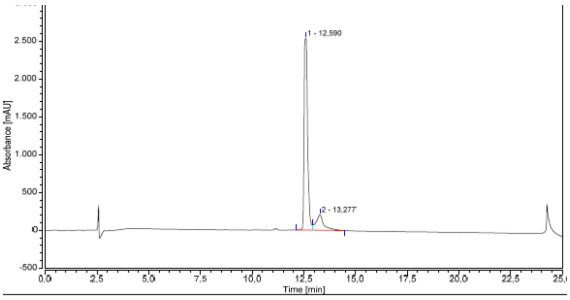

9b

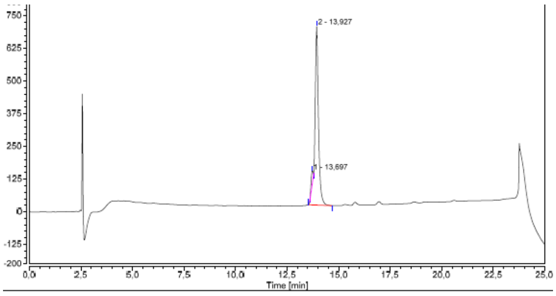

9c

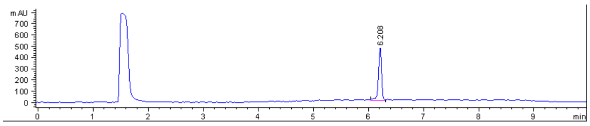

9d

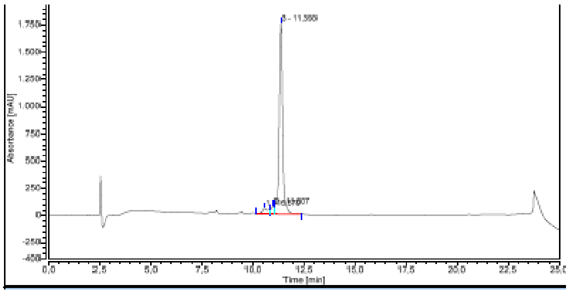

9e

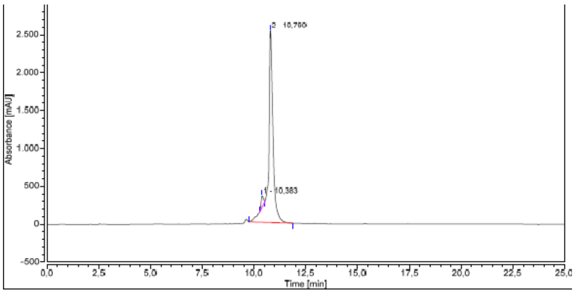

9f

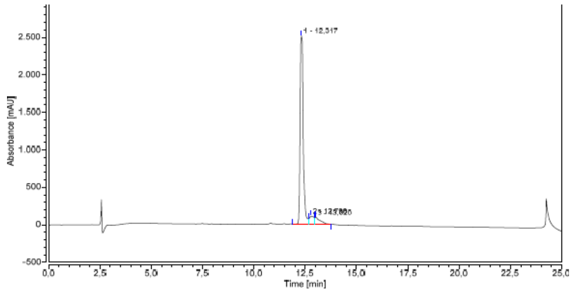

9g

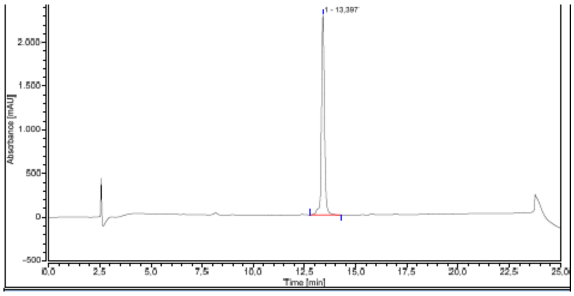

9h

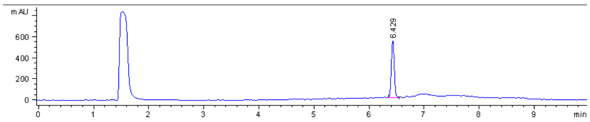

9i

MALDI-spectra

Table S5: MALDI-spectra of macrocycles **7a-j**, **8a-j** and **9a-j**.

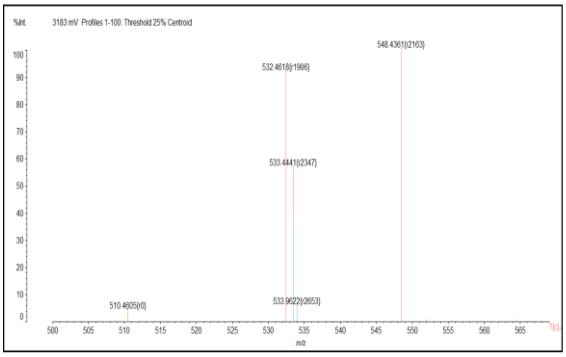<br><br>**7a**	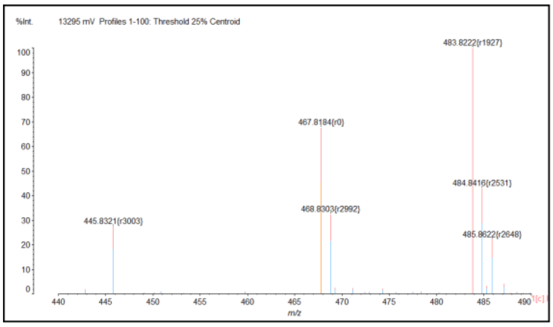<br><br>**7b**
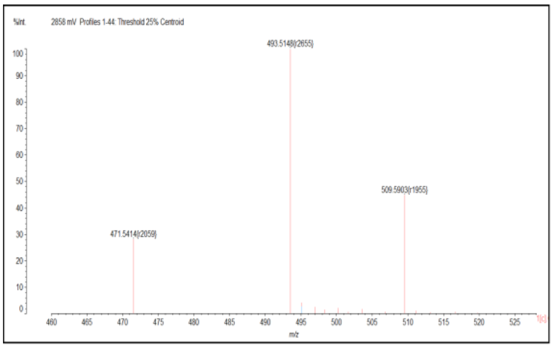<br><br>**7c**	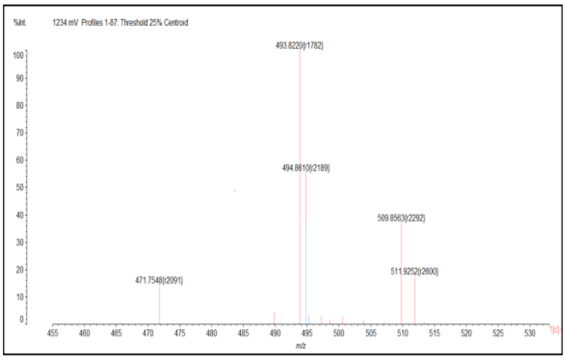<br><br>**7d**
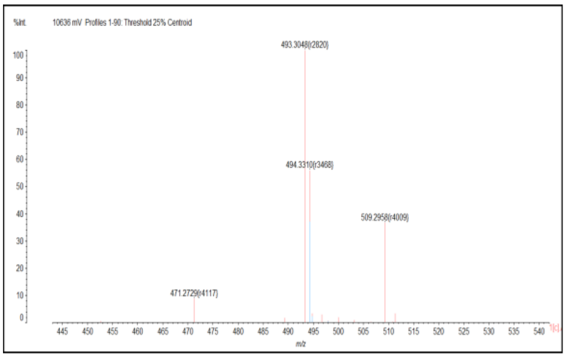<br><br>**7e**	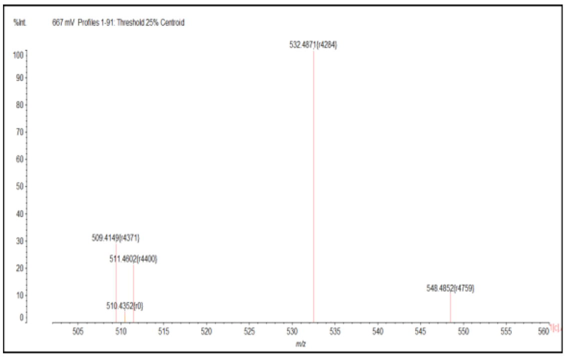<br><br>**7f**
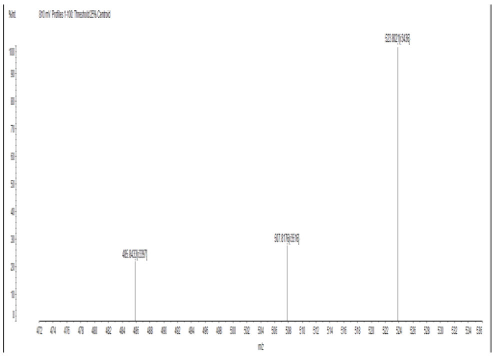<br>**7g**	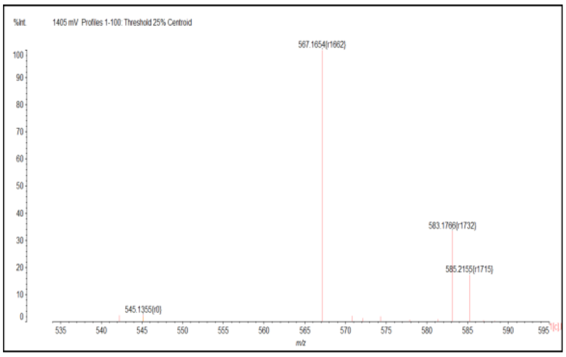<br><br>**7h**

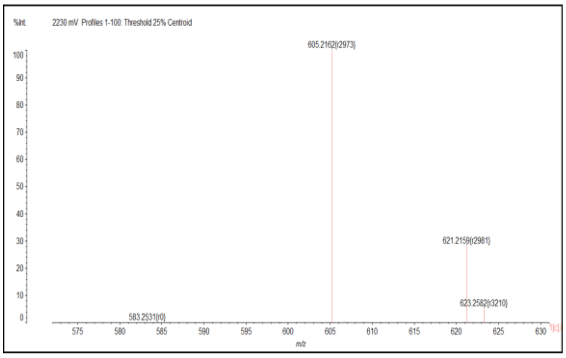

7i

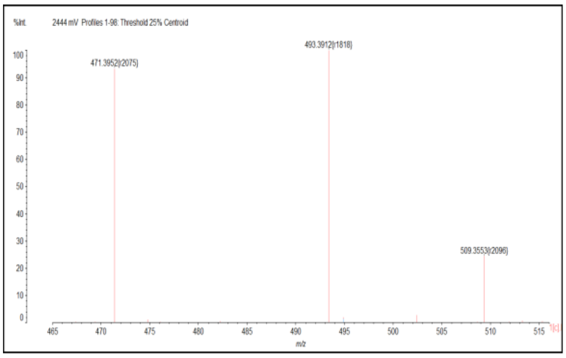

8a

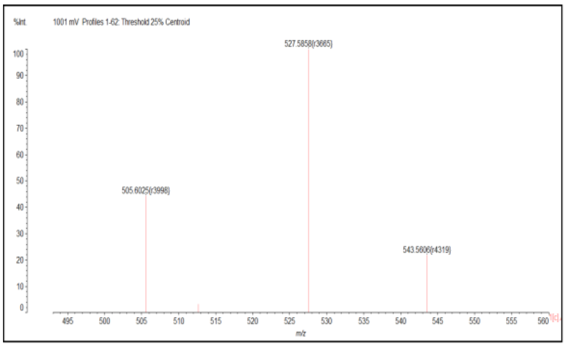

8b

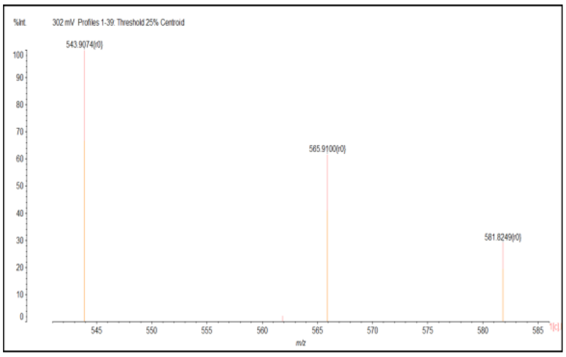

8c

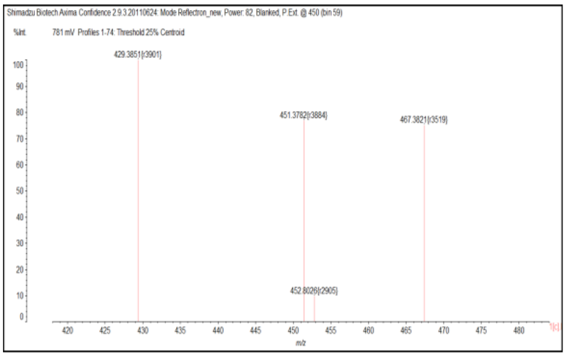

8d

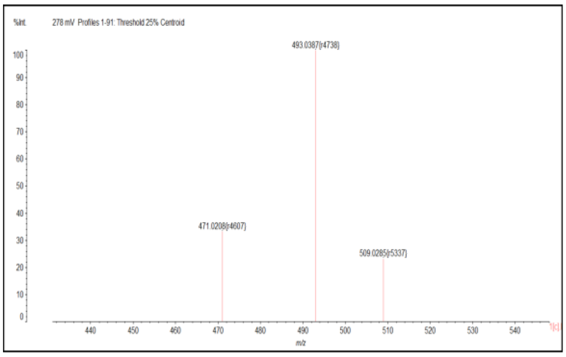

8e

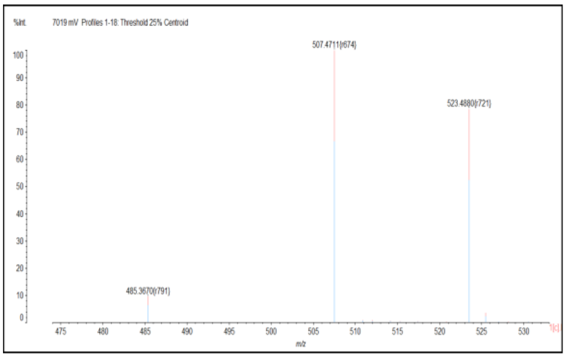

8f

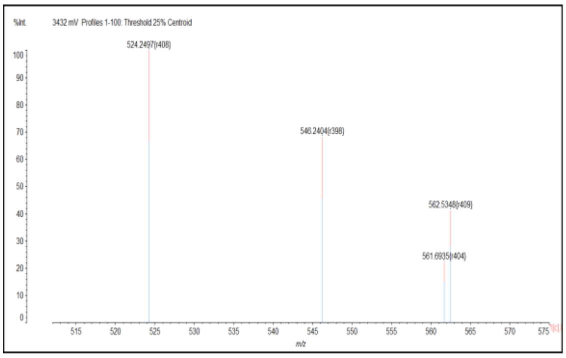

8g

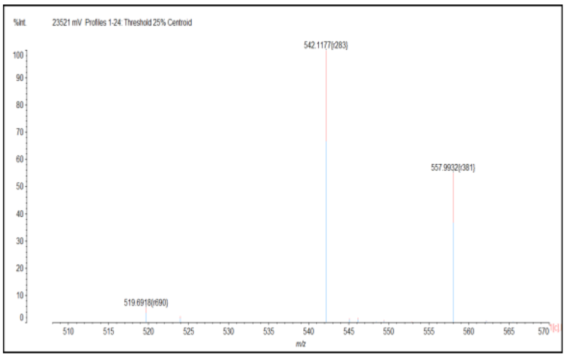

8h

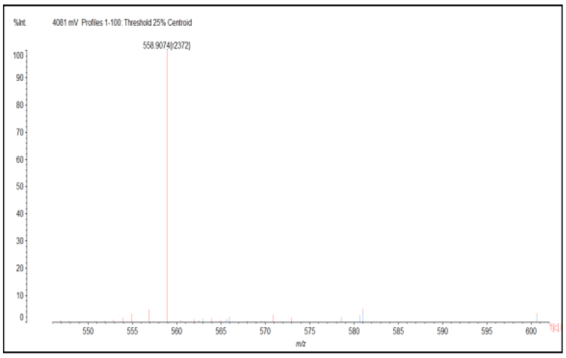

8i

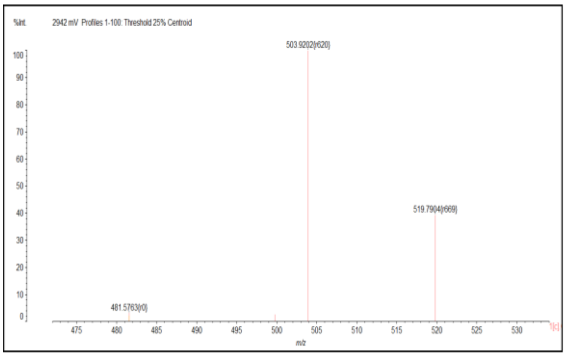

9a

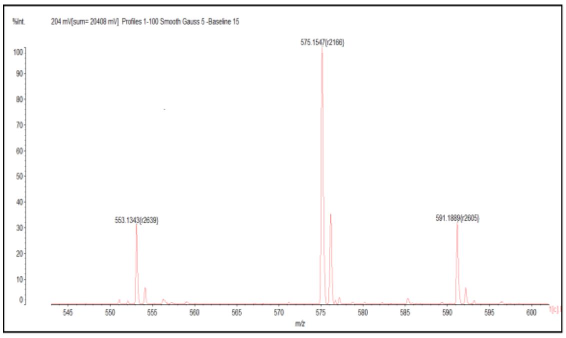

9b

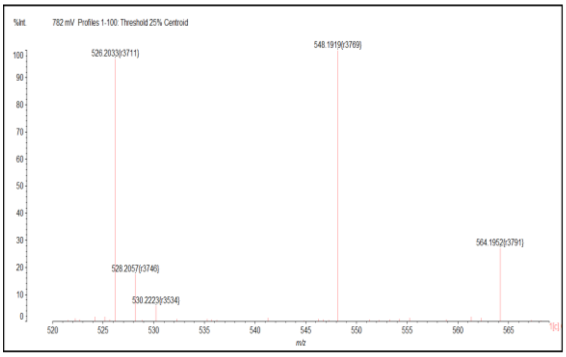

9c

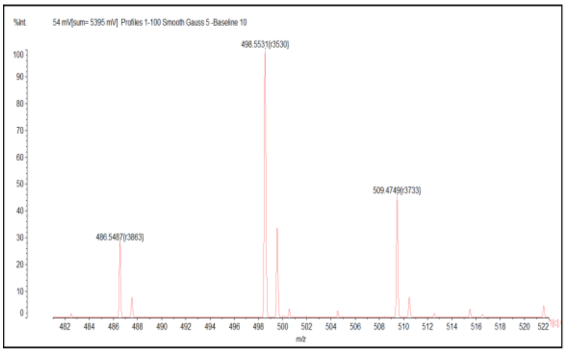

9d

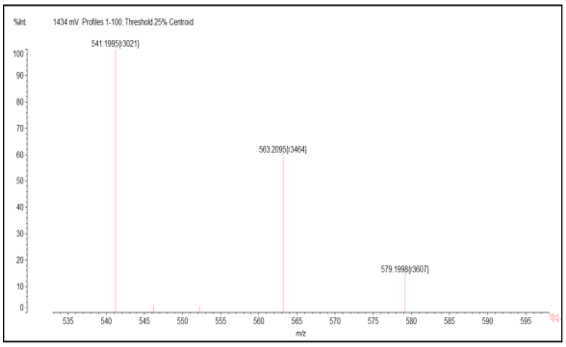

9e

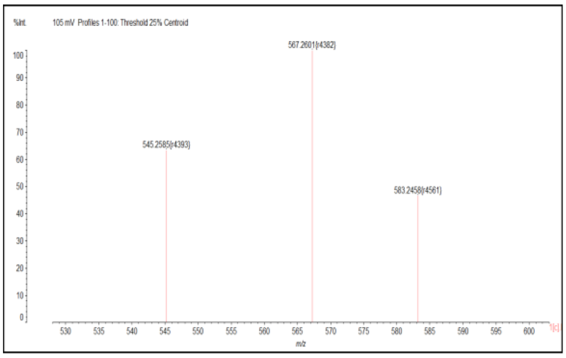

9f

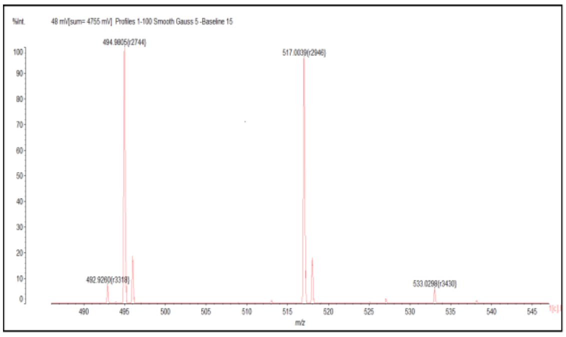

9g

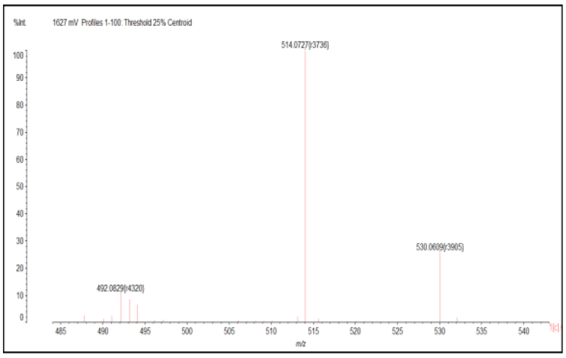

9h

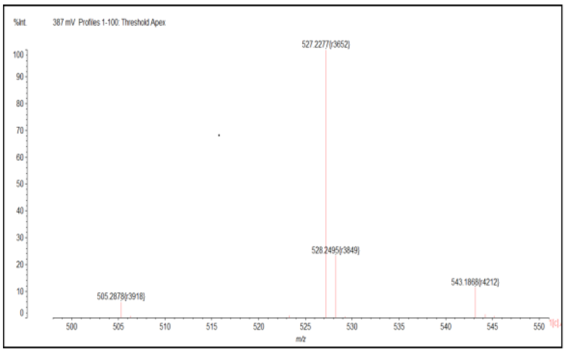

9i

1D-NMR spectra

Table S6: NMR spectra of macrocycles **7a-j**, **8a-j** and **9a-j**.

#	^{1}H-NMR spectrum	^{13}C-NMR spectrum
**7a**	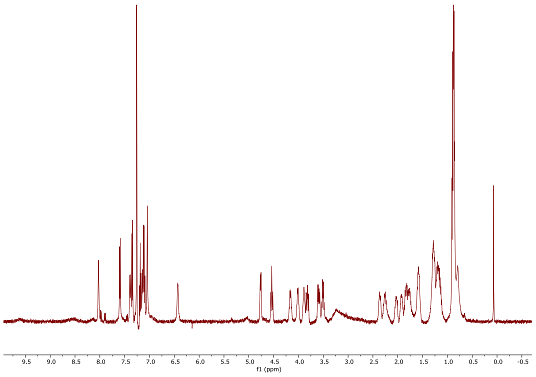	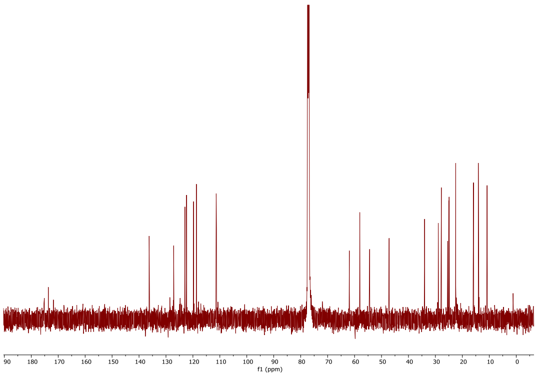
**7b**	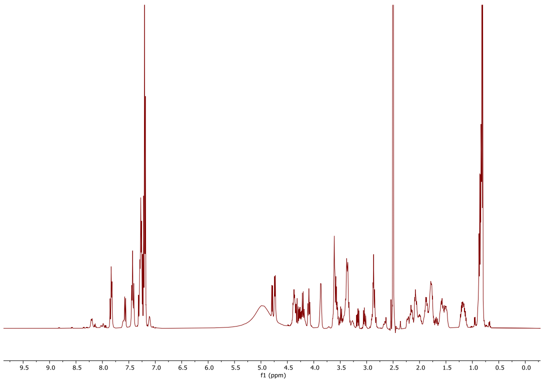	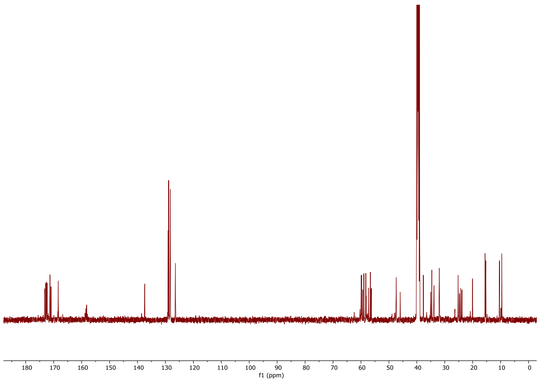

7c

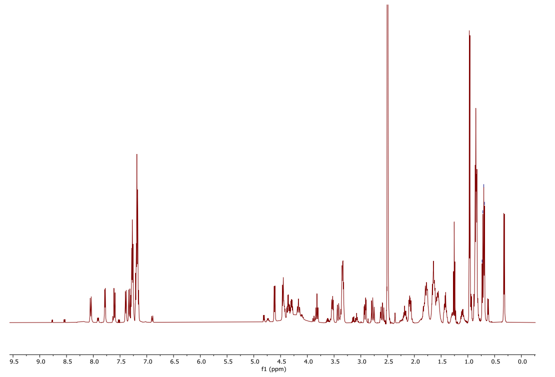

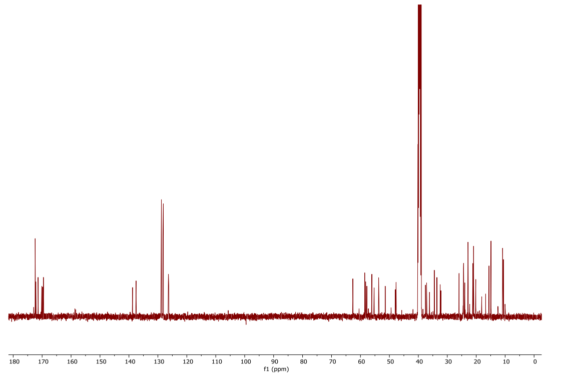

7d

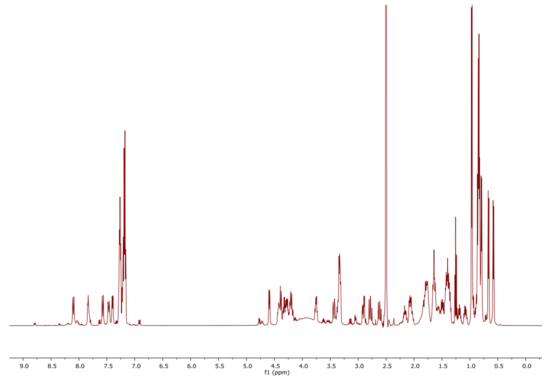

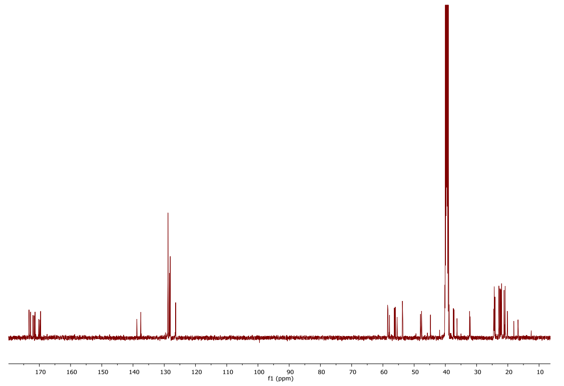

7e

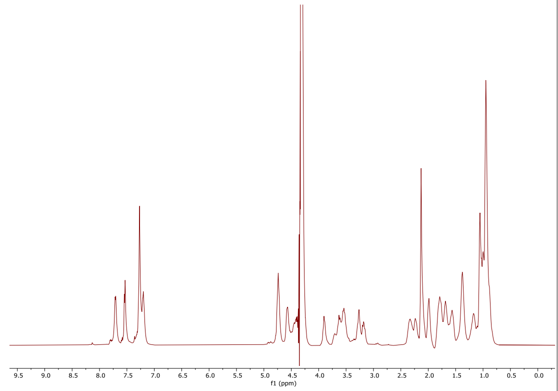

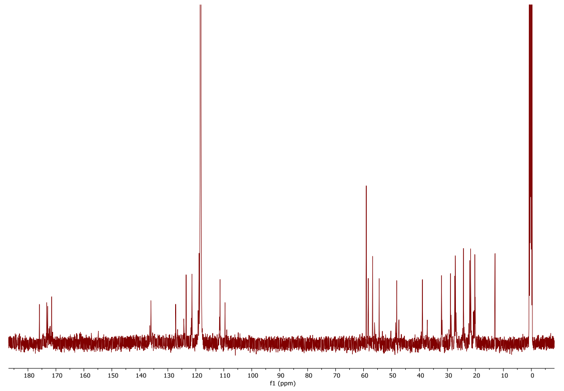

7f

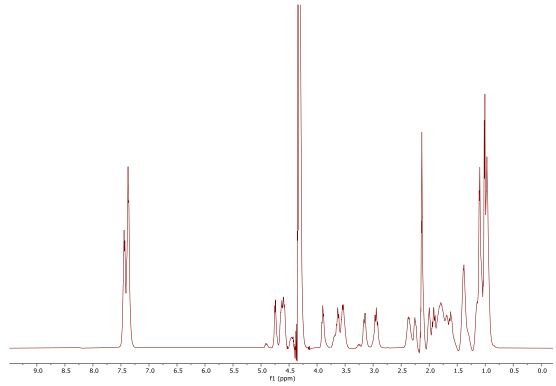

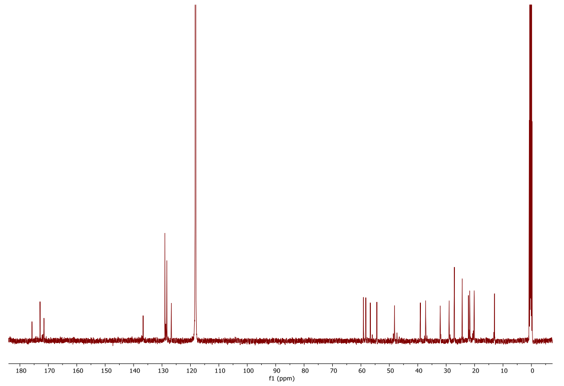

7g

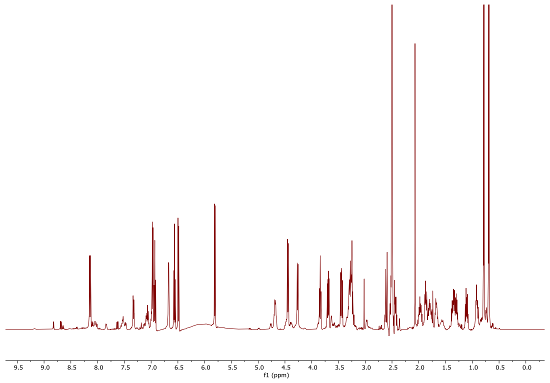

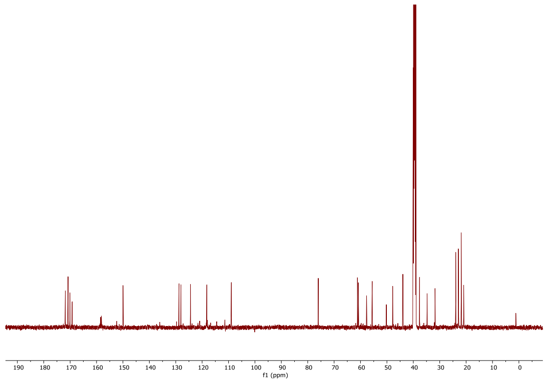

7h

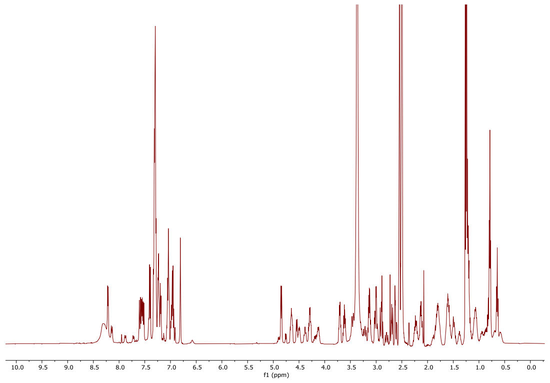

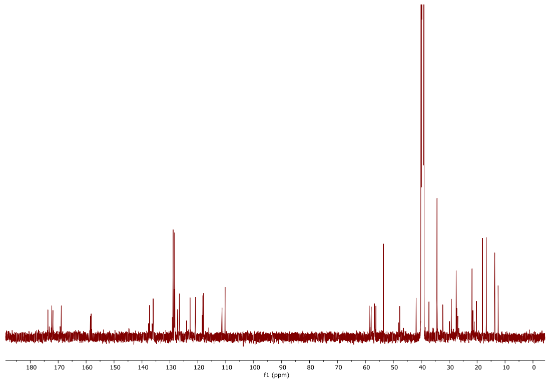

7i

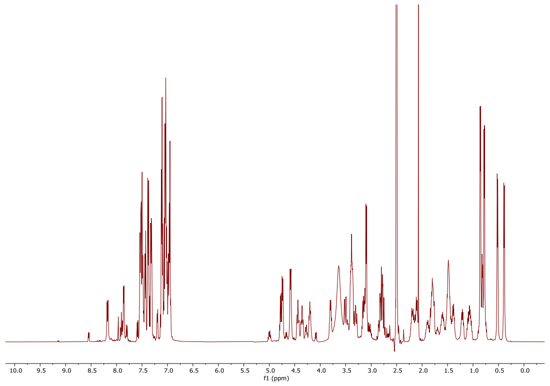

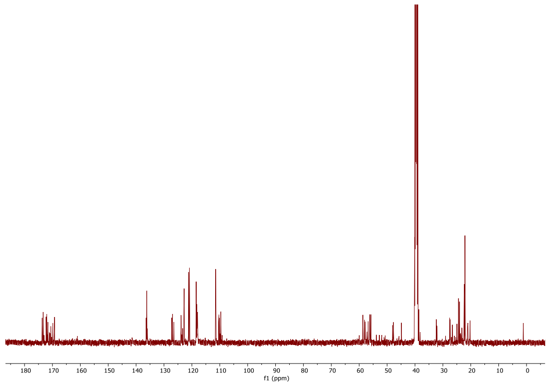

8a

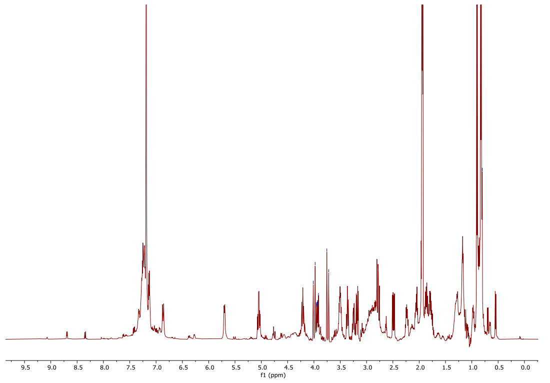

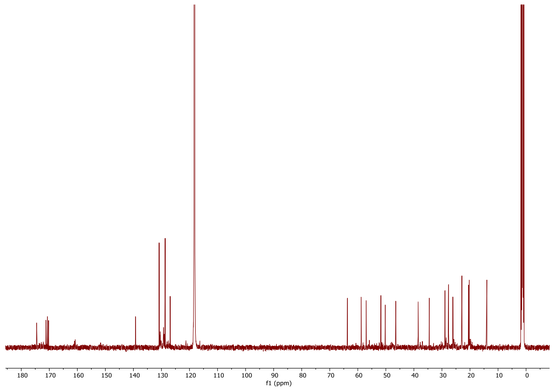

**8b**	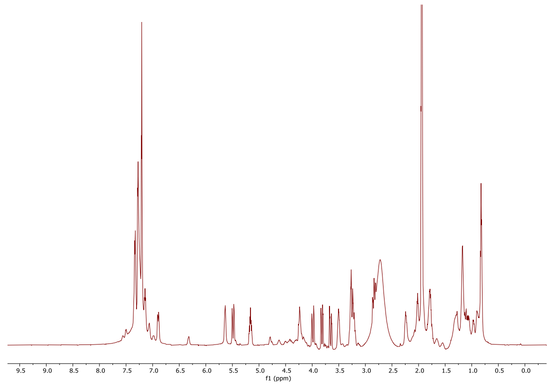	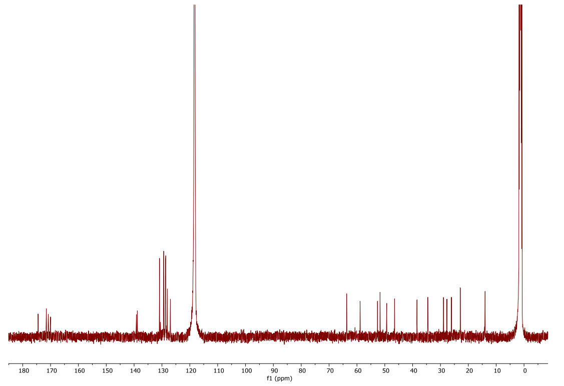
**8c**	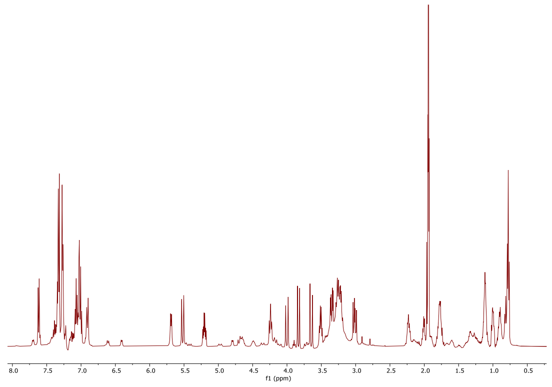	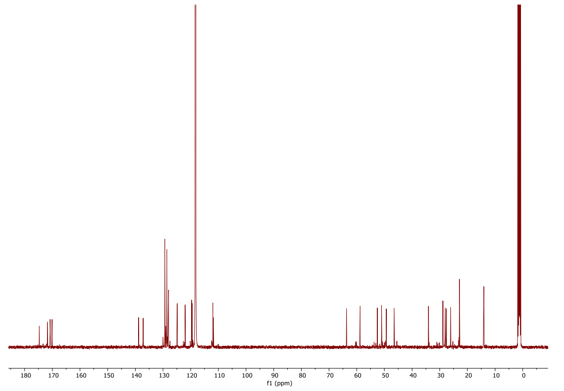
**8d**	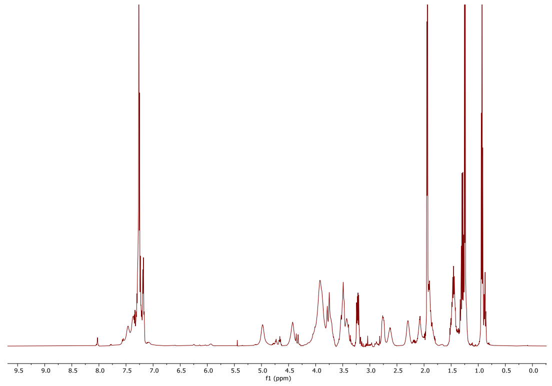	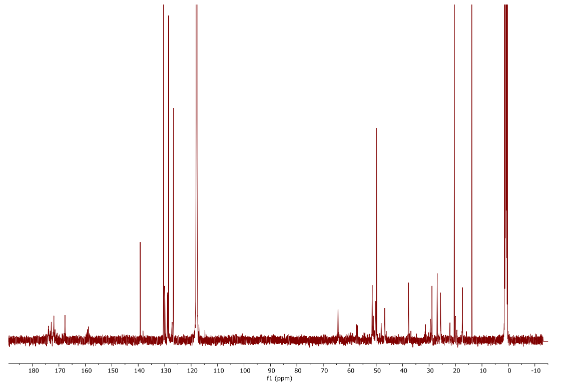
**8e**	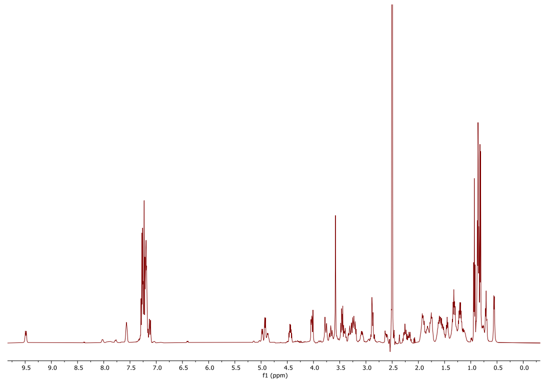	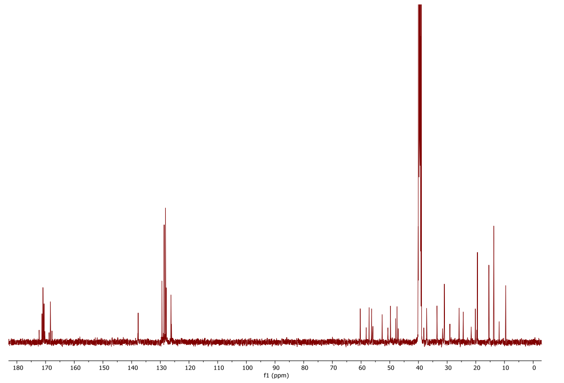

**8f**	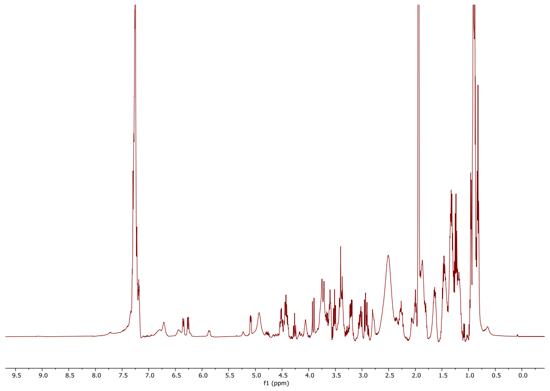	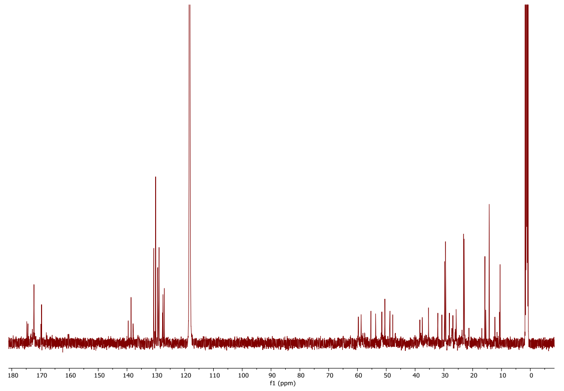
**8g**	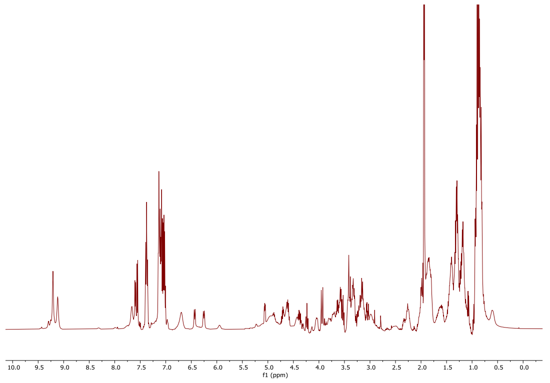	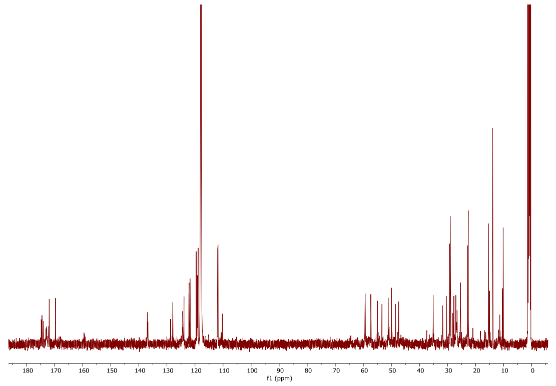
**8h**	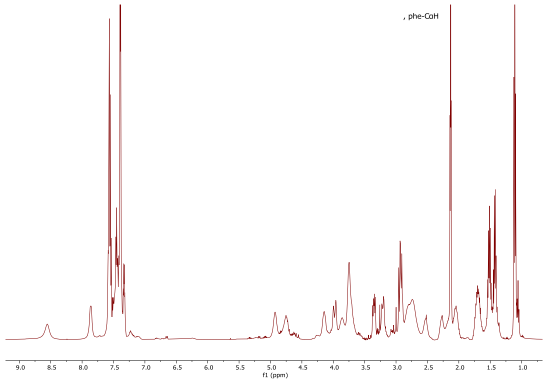	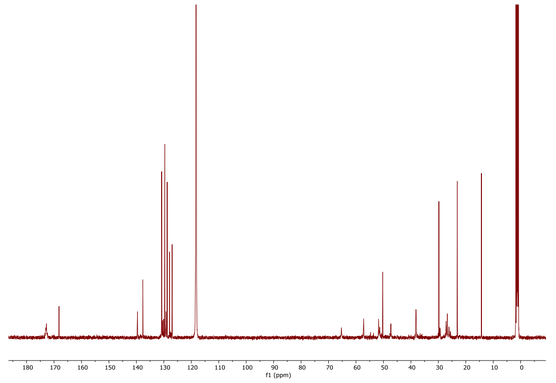
**8i**	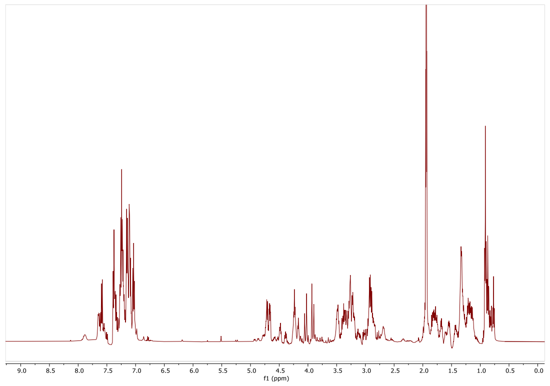	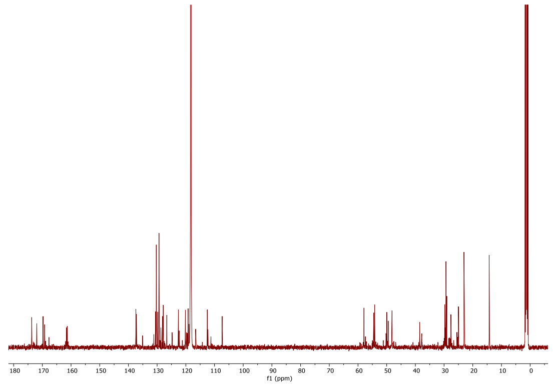

9a

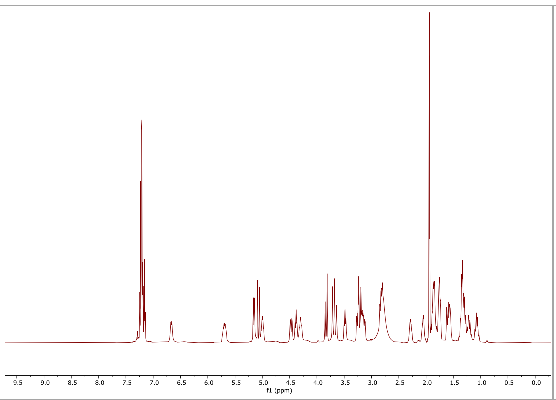

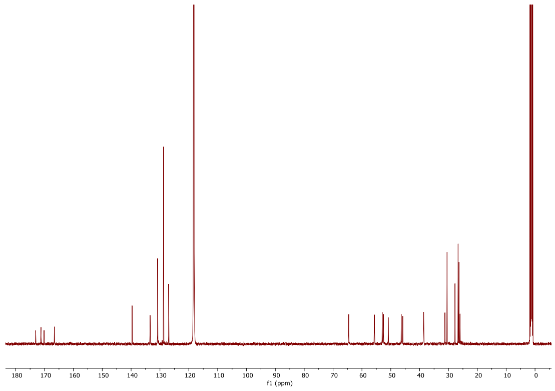

9b

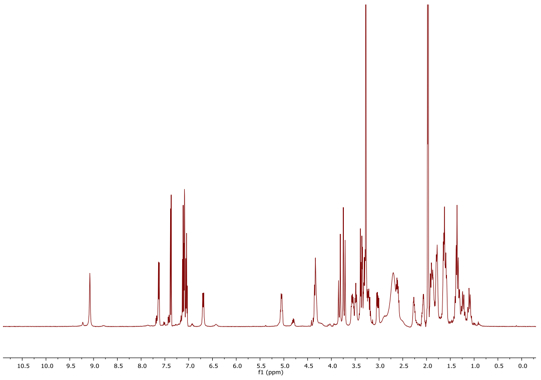

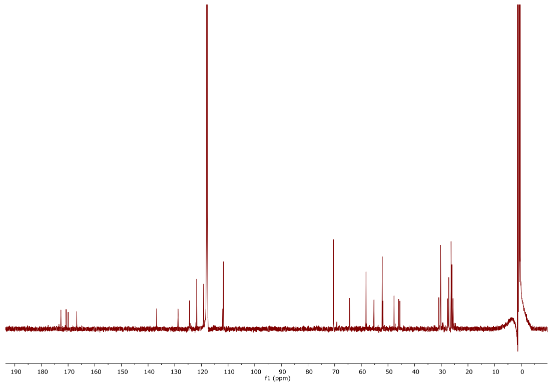

9c

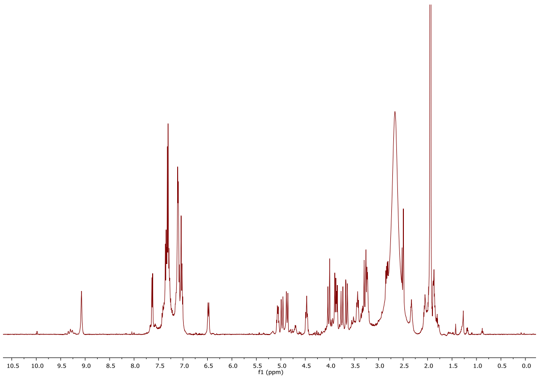

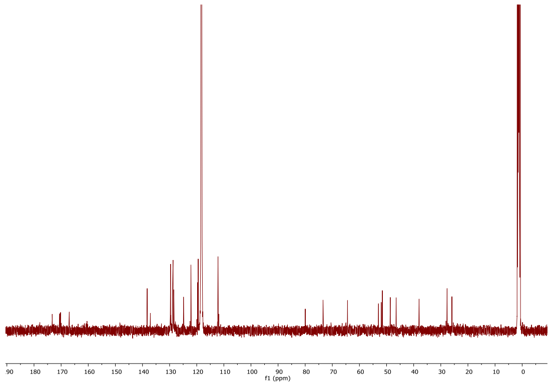

9d

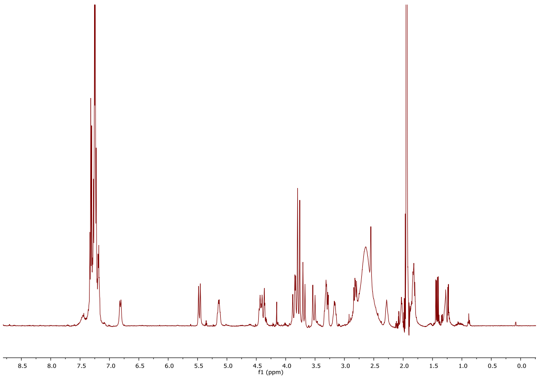

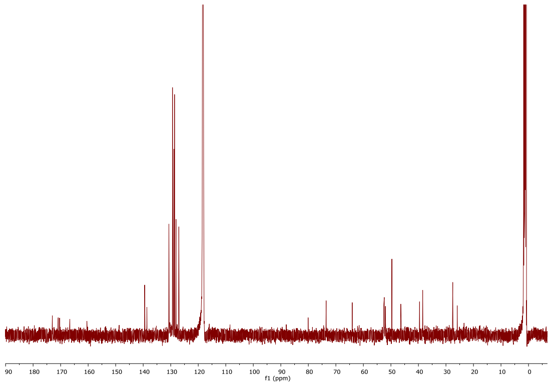

**9e**	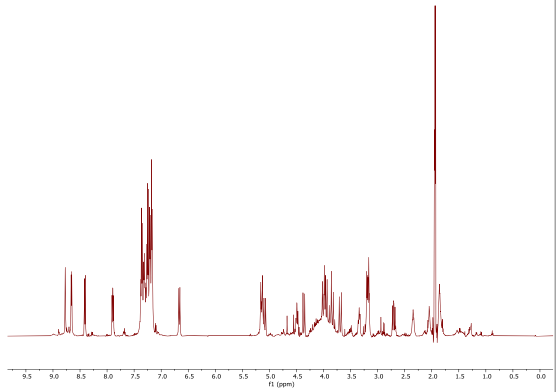	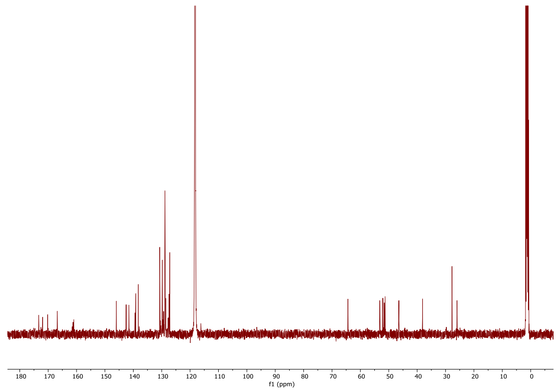
**9f**	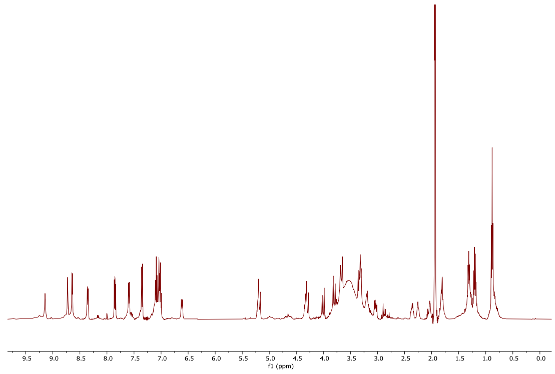	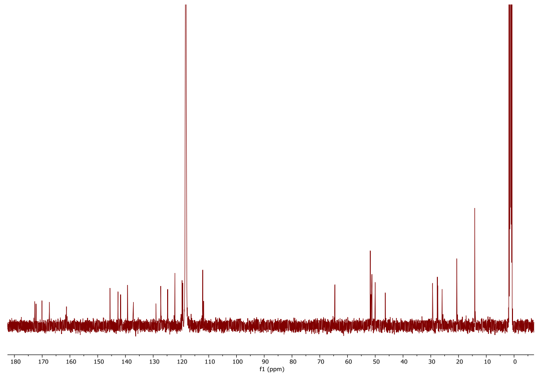
**9g**	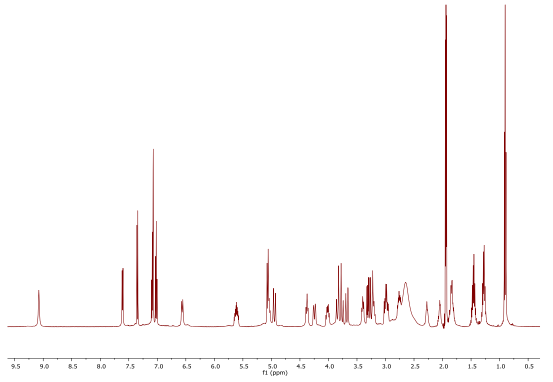	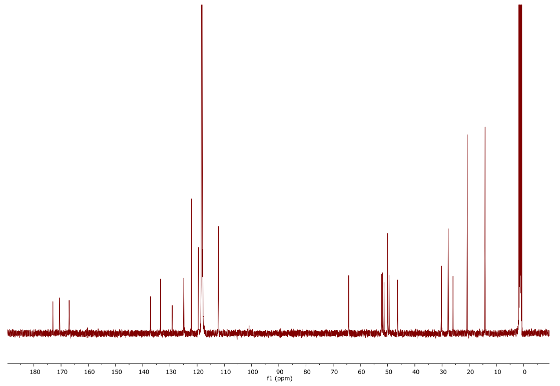
**9h**	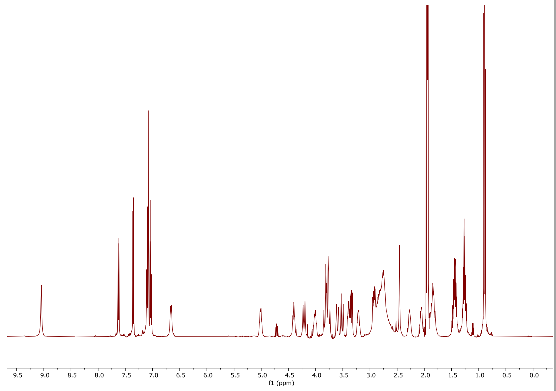	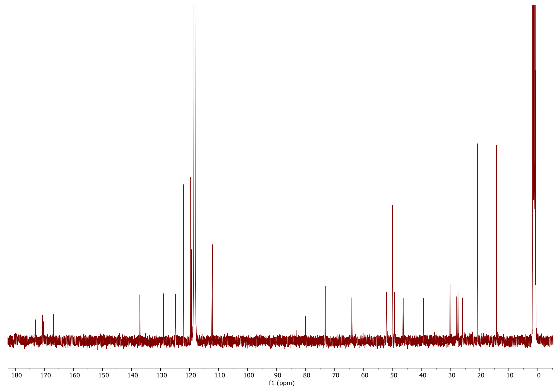

**9i**	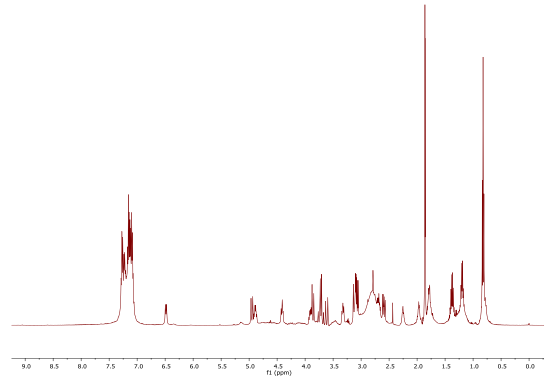	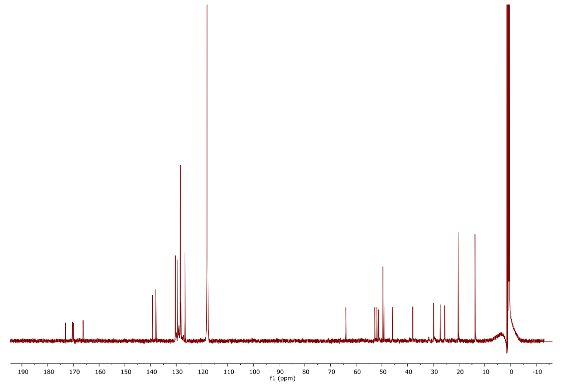

References

1. Enthart, A., Freudenberger, J. C., Furrer, J., Kessler, H.,Luy, B., *J. Magn. Reson.*, 2008, **192**(2), 314-322.
2. Tzvetkova, P., Simova, S.,Luy, B., *J. Magn. Reson.*, 2007, **186**(2), 193-200.
3. Sheldrick, G., *Acta Crystallogr. B*, 2015, **71**(1), 3-8.
4. Sheldrick, G. M., *Acta Crystallogr. C*, 2015, **71**(1), 3-8.
5. Parsons, S., Flack, H. D.,Wagner, T., *Acta Crystallogr. B*, 2013, **69**(3), 249-259.
6. Spek, A., *Acta Crystallogr. D*, 2009, **65**(2), 148-155.
7. Spek, A. L., *Acta Crystallogr. C*, 2015, **71**(1), 9-18.
8. Merrifield, R. B., *J. Am. Chem. Soc.*, 1963, **85**(14), 2149-2154.
9. Zuckermann, R. N., Kerr, J. M., Kent, S. B.,Moos, W. H., *J. Am. Chem. Soc.*, 1992, **114**(26), 10646-10647.
10. Aldrich, J. V., Kulkarni, S. S., Senadheera, S. N., Ross, N. C., Reilley, K. J., Eans, S. O., Ganno, M. L., Murray, T. F.,McLaughlin, J. P., *ChemMedChem*, 2011, **6**(9), 1739-1745.
11. Hanwell, M. D., Curtis, D. E., Lonie, D. C., Vandermeersch, T., Zurek, E.,Hutchison, G. R., *J. Cheminformatics*, 2012, **4**(1), 1-17.
12. Halgren, T. A., *J. Comput. Chem.*, 1996, **17**(5-6), 490-519.
13. Lee, C., Yang, W.,Parr, R. G., *Phys. Rev. B*, 1988, **37**(2), 785.
14. Becke, A. D., *Int. J. Chem. Phys.*, 1993, **98**(2), 1372-1377.
15. Stephens, P. J., Devlin, F. J., Chabalowski, C. F.,Frisch, M. J., *Am. J. Phys. Chem.*, 1994, **98**(45), 11623-11627.
16. Weigend, F.,Ahlrichs, R., *PCCP*, 2005, **7**(18), 3297-3305.
17. Weigend, F., Häser, M., Patzelt, H.,Ahlrichs, R., *Chem. Phys. Lett.*, 1998, **294**(1-3), 143-152.
18. Treutler, O.,Ahlrichs, R., *Int. J. Chem. Phys.*, 1995, **102**(1), 346-354.
19. Klamt, A.,Schüürmann, G., *J. Chem. Soc.*, 1993(5), 799-805.
20. Srinivasan, K.,Kay, R., *J. Solution Chem.*, 1977, **6**(5), 357-367.
21. Gloge, T., *Development of a universal alignment medium for the extraction of RDCs and structure elucidation with tensorial constraints*. 2020, Karlsruhe University of Technology.
22. Navarro-Vázquez, A., *Magn. Reson. Chem.*, 2012, **50**, 73-79.
